# Exon Junction Complexes Show a Distributional Bias toward Alternatively Spliced mRNAs and against mRNAs Coding for Ribosomal Proteins

**DOI:** 10.1016/j.celrep.2016.06.096

**Published:** 2016-07-28

**Authors:** Christian Hauer, Jana Sieber, Thomas Schwarzl, Ina Hollerer, Tomaz Curk, Anne-Marie Alleaume, Matthias W. Hentze, Andreas E. Kulozik

**Affiliations:** 1 Department of Pediatric Oncology, Hematology, and Immunology, University of Heidelberg, Im Neuenheimer Feld 430, 69120 Heidelberg, Germany; 2 Molecular Medicine Partnership Unit (MMPU), Im Neuenheimer Feld 350, 69120 Heidelberg, Germany; 3 European Molecular Biology Laboratory (EMBL) Heidelberg, Meyerhofstrasse 1, 69117 Heidelberg, Germany; 4 Faculty of Computer and Information Science, University of Ljubljana, Vecna Pot 113, 1000 Ljubljana, Slovenia

## Abstract

The exon junction complex (EJC) connects spliced mRNAs to posttranscriptional processes including RNA localization, transport, and regulated degradation. Here, we provide a comprehensive analysis of bona fide EJC binding sites across the transcriptome including all four RNA binding EJC components eIF4A3, BTZ, UPF3B, and RNPS1. Integration of these data sets permits definition of high-confidence EJC deposition sites as well as assessment of whether EJC heterogeneity drives alternative nonsense-mediated mRNA decay pathways. Notably, BTZ (MLN51 or CASC3) emerges as the EJC subunit that is almost exclusively bound to sites 20–24 nucleotides upstream of exon-exon junctions, hence defining EJC positions. By contrast, eIF4A3, UPF3B, and RNPS1 display additional RNA binding sites suggesting accompanying non-EJC functions. Finally, our data show that EJCs are largely distributed across spliced RNAs in an orthodox fashion, with two notable exceptions: an EJC deposition bias in favor of alternatively spliced transcripts and against the mRNAs that encode ribosomal proteins.

## Highlights

• iCLIP analyses of EJC components provide a comprehensive map of bona fide EJCs
• EJC proteins, in particular BTZ, are largely restricted to canonical deposition sites
• EJCs are enriched on alternatively spliced mRNAs
• EJCs are underrepresented on mRNAs encoding ribosomal proteins

## Graphical Abstract

**Figure:**
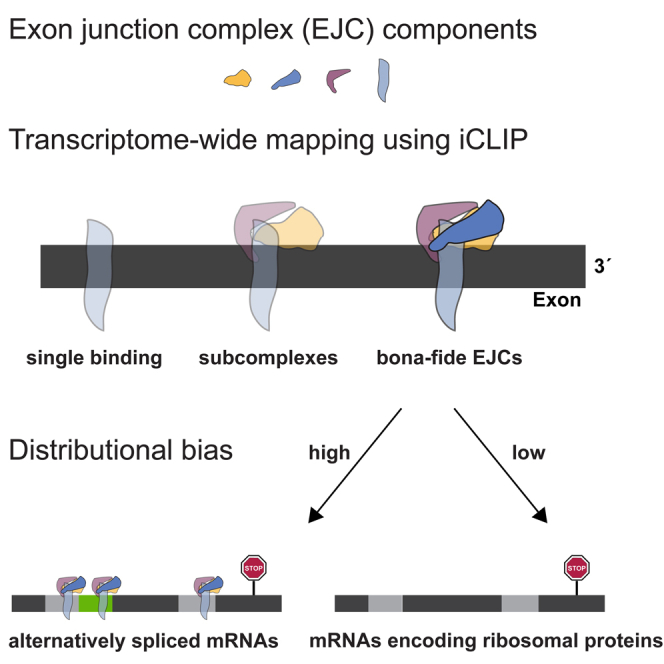

## Teaser

Exon junction complexes govern multiple critical decisions in posttranscriptional gene regulation. Using all four RNA binding subunits of the complex, Hauer et al. provide a comprehensive map of bona fide EJCs across a mammalian transcriptome and show enrichment on alternatively spliced mRNAs and underrepresentation on RNAs encoding ribosomal proteins.

## Introduction

Exon junction complexes (EJCs) are deposited in a splicing-dependent and essentially sequence-independent fashion approximately 20–24 nt upstream of exon-exon boundaries (Le Hir et al., 2000). The mammalian EJC is composed of four core subunits: eukaryotic translation initiation factor 4A3 (eIF4A3), barentsz (BTZ, CASC3, and MLN51), RNA binding protein 8A (RBM8A and Y14), and mago nashi homolog (MAGOH) (Ballut et al., 2005, Degot et al., 2004). In addition, peripheral EJC components bind to the EJC core. These proteins include the up-frameshift protein 3B (UPF3B), the RNA binding protein with serine-rich domain 1 (RNPS1), and the apoptotic chromatin condensation inducer in the nucleus (Acinus) to form a complex of approximately 335 kDa (Kim et al., 2001, Le Hir et al., 2000, Mayeda et al., 1999, Tange et al., 2005). Fully assembled EJCs are dissociated and recycled by the ribosome and the disassembly factor PYM (Gehring et al., 2009b). Because EJC components bind to (pre)-mRNA and remain associated with the mature mRNA until after export, the EJC and its components play important roles in the posttranscriptional fate of the mRNA including (pre)-mRNA splicing (Michelle et al., 2012), export (Le Hir et al., 2001), stability (Gehring et al., 2005, Palacios et al., 2004), translation (Chazal et al., 2013, Nott et al., 2004), and localization (Hachet and Ephrussi, 2004, Palacios et al., 2004).

Considering the broad role of EJCs in posttranscriptional processes, it is important that the abundance of the EJC components varies and is insufficient by orders of magnitude to bind and remain bound to all EJCs of a cell's transcriptome (Gehring et al., 2009b). Previous transcriptome-wide studies used the binding sites of eIF4A3 as a proxy for EJC deposition sites and found that 40%–50% of these sites are located outside the canonical deposition site (Saulière et al., 2012, Singh et al., 2012). By contrast, we provide a comprehensive map of bona fide EJCs across a mammalian transcriptome by using individual nucleotide resolution and immunoprecipitation (iCLIP) analyses for all four RNA binding subunits of the EJC: eIF4A3, BTZ, UPF3B, and RNPS1; the latter two bind to the core EJC and have been directly implicated in alternative nonsense-mediated decay (NMD) pathways (Chan et al., 2007, Chan et al., 2009, Gehring et al., 2005, Huang et al., 2011). In particular, we explored: (1) the distribution of these four EJC subunits across a cell's transcriptome; (2) the subunit composition of EJCs; and (3) quantitative differences for EJC components on functionally defined subsets of mRNAs.

## Results

### Establishment of a Validated Experimental System for Identifying Regions of EJC Enrichment

**Figure 1:**
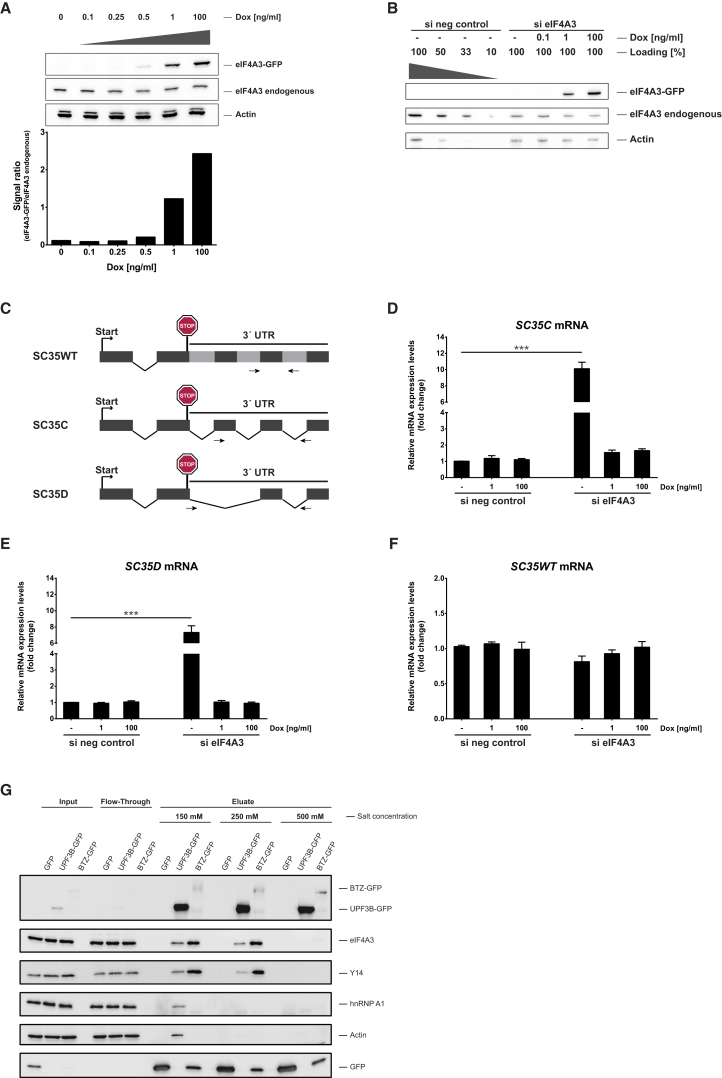
The Experimental HeLa Cell System Expressing Fully Functional EJC-GFP Fusion Proteins Is Suitable for iCLIP Experiments (A) Titration of doxycycline demonstrated a concentration-dependent increase of the eIF4A3-GFP fusion proteins and that a concentration of 1 ng/ml doxycycline was best suited to achieve an expression close to the endogenous level. This image shows a representative immunoblot of three biologically independent experiments. Endogenous and recombinant eIF4A3 were stained concurrently with an α-eIF4A3 antibody. (B) Representative immunoblot after siRNA treatment of three biologically independent experiments is shown. The endogenous and recombinant eIF4A3 were stained concurrently with an α-eIF4A3 antibody. (C) Schematic drawing of three *SC35* mRNA isoforms adapted from Sureau et al. (2001). The grey boxes in *SC35WT* mRNA represent RNA regions that are spliced out in the other isoforms. The arrows show the position of the primers that were used for the amplification of the transcripts (Table S7). (D and E) Upregulation of *SC35C* (D) and *SC35D* (E) transcripts after depletion of endogenous eIF4A3 and rescue of efficient NMD upon induction of the fusion protein with doxycycline. (F) The expression of the NMD-insensitive *SC35WT* isoform did not change under the different conditions. The error bars represent SEM, and p values were calculated by one-way ANOVA with Dunnett's multiple comparison test (^∗∗∗^p value < 0.001 with n = 3–5 independent biological experiments). (G) CoIPs show that the EJC core proteins eIF4A3 and Y14 were stably associated with UPF3B and BTZ under salt concentrations of 150 and 250 mM NaCl and disassembled at 500 mM NaCl. Therefore, 500 mM NaCl was used for the subsequent iCLIP experiments. GFP, BTZ-GFP, and UPF3B-GFP were stained concurrently with an α-GFP antibody. See also Figure S1 and Table S7.

We chose and validated HeLa cells as a system with active NMD (Figure S1A; Boelz et al., 2006) to derive cell lines that stably express inducible genes encoding GFP-tagged eIF4A3, BTZ, UPF3B, and RNPS1, respectively. The induction conditions for the tagged proteins were titrated to correspond to the expression of their endogenous counterparts (Figures 1A and S1B–S1D). The functionality of the GFP-tagged proteins was validated by small interfering (si)RNA complementation and coimmunoprecipitation (coIP) experiments (Figures 1B–1G). The depletion of endogenous eIF4A3 (approx. 30% residual protein) and the induction of eIF4A3-GFP by different doxycycline concentrations is shown in Figure 1B and the effect on endogenous NMD targets was measured by quantitative (q)RT-PCR. These experiments confirmed that the endogenous NMD targets *SC35C* and *SC35D* (Figure 1C; Sureau et al., 2001) were upregulated 8- to 10-fold upon depletion of endogenous eIF4A3 protein (Figures 1D and 1E), whereas the depletion of endogenous eIF4A3 had no effect on the *SC35WT* mRNA that is NMD-insensitive (Figure 1F). Since 1 ng/ml doxycycline induced the expression of eIF4A3-GFP close to that of endogenous eIF4A3 (Figure 1A) and rescued NMD completely (Figures 1D and 1E), we conclude that the eIF4A3-GFP fusion protein is fully active and thus suitable for iCLIP. Successful RNAi and rescue experiments were also performed for HeLa cells expressing either BTZ-GFP or RNPS1-GFP, although the upregulation of the respective NMD targets was less pronounced than for eIF4A3 (Figures S1B and S1C). In case of UPF3B, it has been challenging to test the functionality following RNAi and rescue, which may be related to the function of the highly homologous UPF3A that has been reported both to compensate and to antagonize UPF3B functions (Chan et al., 2009, Shum et al., 2016). We therefore validated UPF3B function biochemically by specific coIP of endogenous eIF4A3 and Y14 upon UPF3B-GFP pull-down (Figure 1G). Taken together, these results validate the GFP fusion proteins for iCLIP experiments.

The iCLIP protocol was optimized as detailed in Experimental Procedures and monitored to guarantee efficient and specific IP, RNA fragmentation, and cDNA library purification. In brief, we adjusted the IP conditions so that only the bait, but not the EJC proteins that do not directly bind the RNA (such as Y14), were detected (500 mM NaCl; Figure 1G). The distribution of the RNA fragments that coimmunoprecipitate with the proteins was adjusted to an optimal size range of 50–300 nt for iCLIP libraries (Figure S2A; Huppertz et al., 2014). After reverse transcription and amplification, the amplicons separated well (Figure S2B) and were purified from primer dimers prior to sequencing (Figure S2C).

### Faithful Mapping of EJCs across the Transcriptome

We achieved highly specific and reproducible iCLIP reads with an excellent signal to noise ratio for all four tested EJC components (eIF4A3, BTZ, UPF3B, and RNPS1) and compared them to the two controls PTB and GFP (Figures S2D–S2F; Table S1). Each RBP was assessed by three biologically independent replicates, and RNA overamplification was avoided (Figure S2F) by including a barcoding system (König et al., 2010). Figure S2F shows that iCLIP experiments with the negative control (GFP) resulted in the expected high overamplification rate. This observation is in line with the low quantity of coimmunoprecipitated RNA fragments (Figure S2A) and validates GFP as a suitable background control. In comparison to GFP, the iCLIP reads of eIF4A3, UPF3B, RNPS1, and in particular BTZ, but not PTB, are highly enriched at splice junctions, demonstrating the specificity and reproducibility of the experiment (Figure S2G).

**Figure 2:**
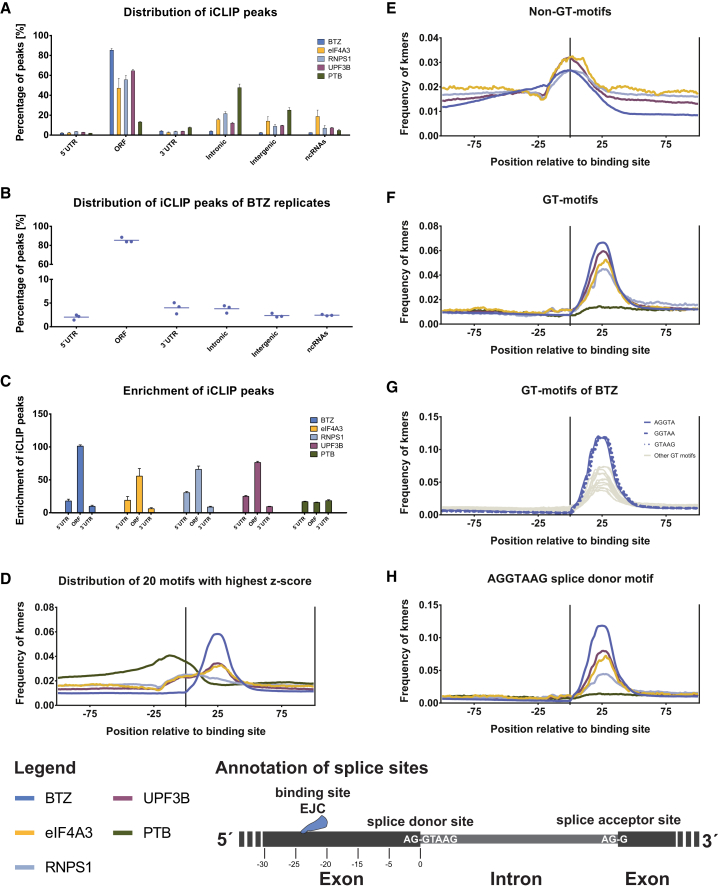
The Distribution of iCLIP Peaks Validates the Predominantly Sequence-Independent Deposition of EJC Proteins at Exonic Region**s** (A) Relative distribution of peaks for all iCLIP data sets confirms that EJC components bind predominantly in the ORF. (B) Distribution of BTZ peaks indicates the high reproducibility of the biological replicates; horizontal lines highlight the mean of three biologically independent BTZ iCLIP replicates. (C) Enrichment of iCLIP peaks at mRNA regions calculated by dividing the number of iCLIP peaks by the cumulative length of indicated regions reveals that the ORF harbors by far the most EJC binding sites, followed by the 5′ UTR, and then the 3′ UTR regions. The error bars represent SEM of n = 3 independent biological experiments. (D–H) The higher enrichment of the splice site signal compared to motifs within the reads confirmed the predominant sequence-independent deposition of the EJC. These graphs display the distribution of 5mer motifs around the binding site of the protein (illustrated as the vertical line in the middle of the plots). (D) Average distribution of the 20 most enriched motifs (see also Table S2) relative to the binding site. (E) Average distribution of 5mers that do not contain GT in their motif. (F) Average distribution of 5mers that do contain GT in their motif. (G) The distribution of GT-containing 5mers in BTZ iCLIP experiments revealed three prominent motifs (AGGTA, GGTAA, and GTAAG). (H) These most enriched GT-containing 5mers concatenate to the canonical splice donor site AGGTAAG, which is enriched in all EJC, but not in the PTB, libraries. See also Figure S2 and Tables S1–S3.

To identify potential differences of EJC binding to different regions of mRNAs, we next analyzed EJC binding to individual sites in a transcript-independent fashion. We used iCount (König et al., 2010, Sugimoto et al., 2012) together with our improved iCLIP analysis tools (Hauer et al., 2015) to detect pronounced binding sites (peaks) in the iCLIP data sets and calculated the percentage of these binding sites in 5′ UTRs, open reading frames (ORFs), 3′ UTRs, intronic or intergenic regions, or non-coding RNAs (ncRNAs), respectively (Figures 2A, S2H, and S2I). The biological specificity of these assignments is highlighted by the PTB iCLIP data set that reflects the known binding preference of this protein for intronic regions (Figure 2A; Han et al., 2014, Spellman and Smith, 2006, Xue et al., 2009). By contrast, EJC proteins display the expected predominant binding to ORFs (Figure 2A), a highly reproducible pattern between individual replicates (exemplified for BTZ in Figure 2B). Correlating the number of EJC subunit binding sites in the ORF and UTRs to the cumulative length of the respective regions demonstrated predominant binding of EJC proteins to coding regions and the preference for the 5′ UTR over the 3′ UTR, as expected (Figure 2C), as 5′ UTRs harbor about three times more introns than 3′ UTRs (Bicknell et al., 2012). The lower signal in the 5′ UTR compared to the ORF may arise from a proportion of EJC-free 5′ UTRs. The 5′ UTR is thought to be EJC-free when the first intron of the pre-mRNA is located more than 25 nt downstream of the start codon (Le Hir et al., 2000). In general, these analyses thus confirmed the expected and specific binding pattern of EJC components to the ORF.

The strong enrichment and high coverage of EJC binding sites in the ORF enabled the exploration of the postulated sequence-independent (Shibuya et al., 2004) as well as the low-stringency conserved sequence-dependent deposition of EJCs (Saulière et al., 2012, Singh et al., 2012). To discover potential sequence motifs, the region around iCLIP peaks was scanned for all possible combinations of five consecutive nucleotides (5mers). The specificity of the motif search was controlled by GFP and PTB data. The analysis of the GFP data set shows predominantly unspecific binding to the immunoprecipitated RNA indicated by low *Z* scores and “enriched motifs” that match recently published GFP background controls for PAR-CLIP (Table S2; Friedersdorf and Keene, 2014). By contrast, the 20 most highly enriched motifs for PTB reach higher *Z* scores and show the expected preference for polypyrimidine tracts (Table S2; Spellman and Smith, 2006). For the four EJC components, the average positional distributions of the 20 most frequent 5mers were slightly increased directly at the binding site, accompanied by a stronger enrichment downstream (Figure 2D). Interestingly, this motif profile for the EJC proteins could be separated into two distinct motif classes: one non-GT-containing (Figure 2E) and one GT-containing (Figure 2F) set of 5mers. The GT-containing 5mers cluster 25 nt downstream of the non-GT-containing ones (Figure 2F), perfectly matching the position of splice junctions. The distribution of all GT-motifs relative to the binding site of BTZ further revealed that three GT-motifs are even more highly enriched at this position (Figure 2G). Remarkably, these motifs concatenate to the canonical splice donor site 5′-AGGTAAG-3′, directly validating the canonical EJC position. The direct search for the canonical splice donor site motif revealed that all four EJC proteins, but not PTB, bind closely upstream of the splice site (Figure 2H).

By contrast, the weakly enriched non-GT-containing 5mers map to the EJC binding site, indicating minor sequence preferences (Figure 2E). This class of motifs includes 5′-GAAGA-3′ as the top motif for RNPS1, UPF3B, and BTZ (Table S2). For eIF4A3, CG-dinucleotides are enriched (Table S2), supporting a previous transcriptome-wide study of eIF4A3 using RNA-IP (RIP) (Singh et al., 2012), while it contrasts with published HITS-CLIP data (Saulière et al., 2012). Thus, EJC components exhibit a weak sequence preference, but the key determinant for EJC deposition across the transcriptome is its position vis-à-vis the splice junction (Figures 2E–2H).

### mRNAs Are Differentially Marked by EJCs

We next tested the binding preferences of the EJC components to different types of RNA. We therefore directly compared EJC-bound RNAs to those bound by PTB using the differential analysis software edgeR (Robinson et al., 2010). We used the comparison to PTB to avoid bias by RNA abundance and because the PTB data set included >80% unique binding sites that are not shared with any of the EJC components (see restricted peaks to one RBP in Table S3).

**Figure 3:**
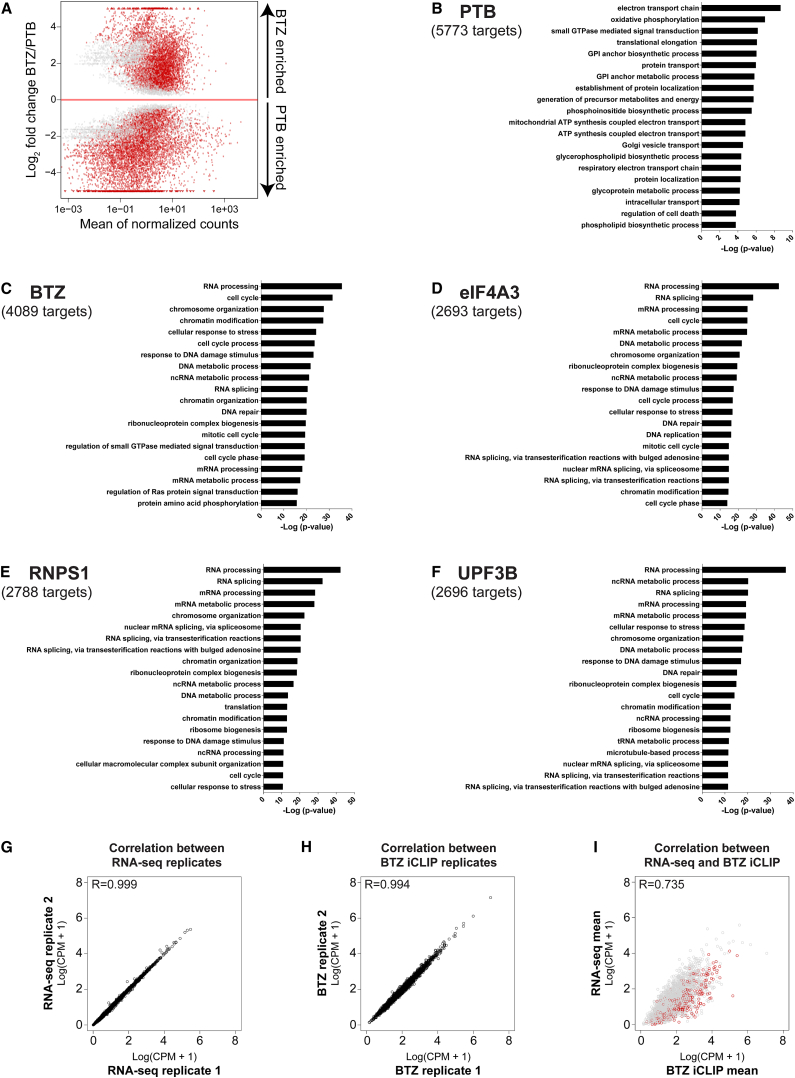
Binding of All EJC Components Is Highly Enriched to Transcripts Coding for RNA Processing Proteins (A–F) The red dots show significant mRNA targets either up- or downregulated after differential analysis using edgeR controlled by the Benjamini-Hochberg procedure with an FDR <0.05. The targets below the red line provided a higher PTB signal, whereas targets above the red line exhibited a higher BTZ signal (A). The targets below the red line were used for GO enrichment analysis of PTB (B). All EJC iCLIP data sets were compared to PTB, and the targets with a log_2_ fold change >0 (above red line) were analyzed for GO enrichment for BTZ (C), eIF4A3 (D), RNPS1 (E), and UPF3B (F). (G–I) Enriched EJC occupancy does not correlate with mRNA abundance. These plots display the correlation of iCLIP and RNA-seq data using the 2,194 common targets that were significantly enriched in BTZ iCLIP in both differential analyses (compared to PTB and GFP). (G and H) The RNA-seq (G) and BTZ iCLIP (H) replicates were highly reproducible. (I) Relationship between the mean count of all three RNA-seq and BTZ iCLIP libraries. The red dots highlight mRNA targets for BTZ with a log_2_ fold change >3 compared to PTB in the differential analysis using edgeR and thus indicate mRNAs that are particularly strongly bound by BTZ. The specificity of these highly occupied mRNAs is demonstrated by the finding that these mRNAs were distributed across transcripts with high and low expression levels as measured by RNA-seq (CPM and R = Pearson's correlation coefficient). See also Figure S3.

This differential analysis separated mRNAs that are either enriched for EJC binding or for PTB binding at a false discovery rate (FDR) of <5% (Figures 3A and S3). We then performed gene ontology (GO) enrichment analysis for these two categories. Interestingly, this analysis revealed that all EJC subunits enrich on mRNAs that encode proteins that are involved in RNA processing, cell-cycle control, or chromosome organization. By contrast, PTB-bound RNAs are enriched for those encoding proteins with other functions such as electron and protein transport (Figures 3B–3F). Similar results were obtained for the EJC subunits when the differential analysis used GFP as a control (Figure S3).

Next, we selected common targets (n = 2,194) that are significantly enriched for BTZ binding sites in both differential analyses (compared to PTB and GFP) to reduce the number of false positive assignments. With this reliable subset, we were able to control the enrichment of EJC proteins on specific targets for mRNA abundance. Both, the RNA-sequencing (seq) and the BTZ iCLIP analyses show a high degree of reproducibility between independent biological replicates (Figures 3G and 3H). In general, BTZ iCLIP counts correlate well with RNA-abundance (Figure 3I). However, the majority of the targets that show the strongest enrichment in binding when compared to PTB (Figure 3A) also exhibit a higher BTZ iCLIP than RNA-seq signal (highlighted as red dots in Figure 3I). Remarkably, these mRNAs are distributed broadly across low and high abundance mRNAs in the cell. Thus, these data demonstrate: (1) a particularly pronounced association of EJCs with the mRNAs enriched in the functional categories outlined above and (2) independence of EJC association from RNA abundance per se (Figure 3I).

### The Majority of Exons Harbor EJCs at the Canonical Deposition Sites

**Figure 4:**
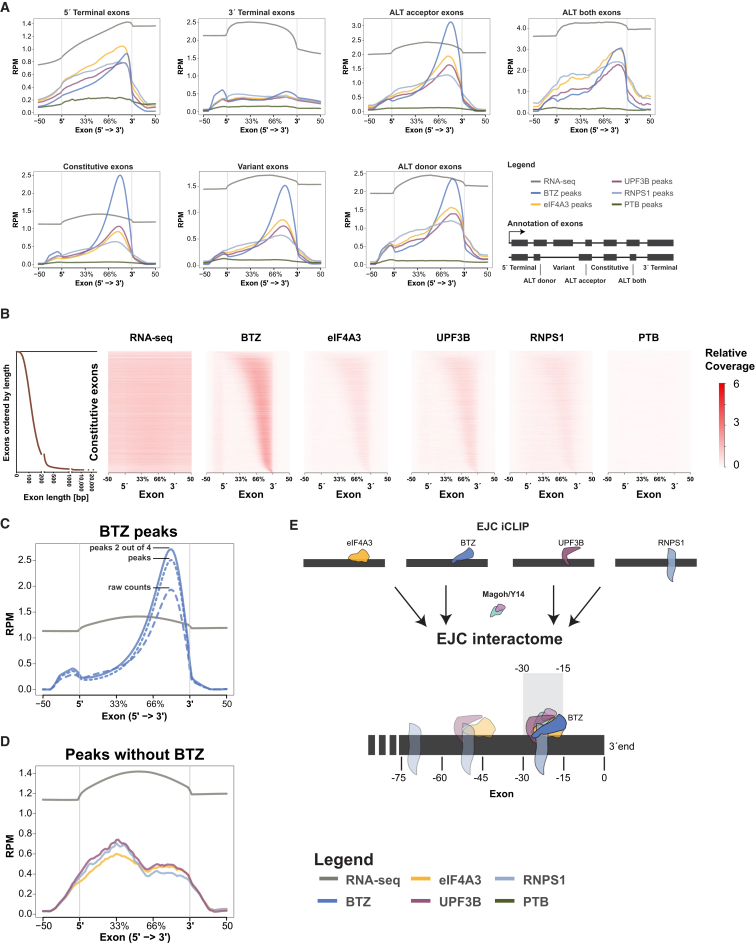
High-Confidence EJC Binding Sites Reveal that BTZ Is an Essential EJC Component at the Canonical Deposition Site (A) Internal exons are predominantly bound by EJC components and constitutive exons display the strongest BTZ signal relative to their abundance. The average profile of reads covering exons are plotted for: (1) 5′ terminal exons; (2) 3′ terminal exons; (3) constitutive exons present in all isoforms; (4) variant exons not present in all isoforms; (5) exons with alternative acceptor sites (ALT acceptor); (6) exons with alternative donor sites (ALT donor); and (7) exons with both alternative donor and acceptor sites (ALT both) using ngsplot software (Shen et al., 2014). (B) Histogram on the left hand side shows the length of the constitutive exons and is aligned to the heatmaps showing RNA-seq and iCLIP coverage across individual exons ordered by their length. The length of the exons can be extracted from the histogram. This image confirms that most exons harbor an EJC at the 3′ end and demonstrate that the EJC signal strength is independent of exon length. The color key represents the signal strengths of the RNA-seq and iCLIP data. (C) Average coverage profiles across constitutive exons for BTZ iCLIP: (1) raw reads; (2) reads in peaks; and (3) reads in peaks that overlap with at least one of the other EJC proteins (eIF4A3, UPF3B, and RNPS1) are restricted to the 3′ end of exons. The peak detection and filtering approaches increased the BTZ signal. (D) By contrast, the average exon profiles for eIF4A3, UPF3B, and RNPS1 iCLIP binding sites that were not determined concurrently by BTZ binding sites were absent of an EJC signal close to the 3′ end of exons suggesting that non-canonical binding sites do not contain the fully assembled EJC. The profiles are plotted as read CPM mapped reads (RPM). (E) The integrated analyses over four distinct components of the EJC revealed that BTZ determines the position of fully assembled EJCs to the canonical deposition sites at 15–30 nt upstream of exon-exon junctions. Non-canonical depositions sites of EJC proteins (alone or in subcomplexes) located in other regions of the exon are less common and do not contain BTZ. See also Figure S4 and Tables S4–S6.

Next, we set out to analyze EJC composition in a systematic manner and thus classified exons in protein-encoding mRNAs into seven categories: (1) 5′ terminal exons; (2) constitutive exons present in all isoforms; (3) variant exons not present in all isoforms; (4) exons with alternative donor sites; (5) exons with alternative acceptor sites; (6) exons with both alternative donor and acceptor sites; and (7) 3′ terminal exons. We first considered all mRNAs and found that both constitutive and alternatively spliced exons are homogenously engaged with all four EJC components with the expected exception of 3′ terminal exons (Figure 4A; see Table S4 for statistics). To obtain a more detailed view for each individual exon, we plotted the binding sites of all four EJC components for each of the 123,585 constitutive exons as a single row in a heatmap (Figure 4B). As in the composite plots, BTZ exhibits the strongest signal, followed by eIF4A3, UPF3B, and RNPS1, whereas the PTB control shows the expected flat profile. In the heatmap, the length of each exon was set to a relative length of 100% and subsequently ordered by its absolute length. While the EJC signal displays the expected downstream shift with increasing length, the strength of the EJC signal does not depend on exon length, indicating that exon length does not determine the degree of EJC occupancy, which is consistent with the expectation that an exon generally binds only one EJC. However, very short exons show a considerably decreased EJC signal, fitting with earlier findings that exons shorter than 20 nt do not harbor an EJC (Gehring et al., 2009a).

These data thus indicate that >80% of fully assembled EJCs (as determined by coincident signals for all four tested subunits) are deposited at the expected sites 20–24 nt upstream of exon-exon junctions, contrasting with previous reports focusing on eIF4A3 binding alone, which suggested that 40%–50% of EJC recruitment occurs outside the 20–24 nt region (Saulière et al., 2012, Singh et al., 2012). Further, we directly show that binding clusters around the −24 position in a metaplot showing the iCLIP reads of BTZ at exon-exon junctions (Figure S4A).

### BTZ Determines the Canonical EJC Deposition Site

To elucidate whether EJC composition is homogeneous or heterogeneous, we further enriched for the presence of bona fide EJC binding sites by defining high-confidence EJC deposition sites as those that bind at least two of the four EJC proteins (Figures 4C and 4D; see the Supplemental Information for filtering details). At least two of the four proteins could be detected on 59,521 sites across the whole transcriptome (Table S3). Remarkably, BTZ binding marks 53,812 of these sites, indicating that BTZ peaks offer the best single EJC protein approximation of bona fide EJC deposition sites (Table S3). Figure 4C illustrates the increasing density of BTZ deposition sites for three subsets of reads: (1) raw reads; (2) reads in peaks; and (3) high-confidence reads in peaks that overlapped with at least one of the other EJC proteins (eIF4A3, UPF3B, and RNPS1).

The interpretation of BTZ representing an essential EJC component and a valid marker for EJCs is reinforced by examination of common peaks between the other three EJC proteins (eIF4A3, UPF3B, and RNPS1) not coinciding with BTZ peaks. The average profiles of such peaks are enriched toward the 5′ rather than 3′ end of exons, thus likely representing RNP complexes that do not function as EJCs (Figure 4D).

**Figure 5:**
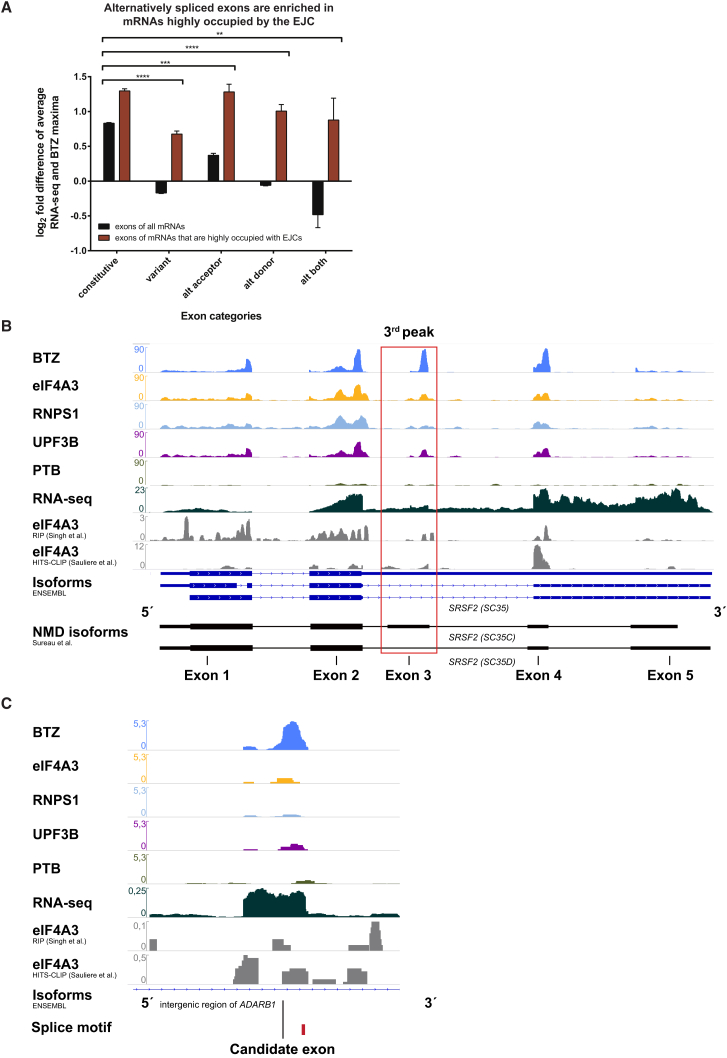
Binding of EJC Components Is Highly Enriched at Alternatively Spliced Exons in Transcripts with High EJC Occupancy and Enables Detection of Low-Abundance NMD-Sensitive mRNA Isoforms (A) We used the RNA-seq and BTZ maxima as shown in Figure 4 (see also Table S4) to calculate a log_2_ fold difference for both all mRNAs and mRNAs that are highly occupied by EJCs. The iCLIP/RNA-seq ratio is enriched for alternatively spliced exons in RNAs that are highly occupied by EJCs (see Figures 3G–3I) compared to all mRNAs. (B) Genome browser view of *SRSF2* (*SC35*) gene reveals EJC iCLIP peaks on exons corresponding to NMD-sensitive *SC35C* and *SC35D* mRNA isoforms. The NMD-insensitive *SC35WT* isoform is displayed in the Ensembl genes track as the upper isoform. The red box highlights the variant exon 3. (C) Candidate exon in the intronic region of the mRNA *ADARB1*. The track range displays CPM and was adjusted to the highest iCLIP signal obtained in the iCLIP libraries of this study in each genome browser view. The signals of the RNA-seq and literature data were not adjusted. The literature data were obtained from RIP (Singh et al., 2012) and HITS-CLIP (Saulière et al., 2012) of eIF4A3. See also Figures S4 and S6.

Since the composition of the EJCs displays remarkable homogeneity, we took an in-depth look at mRNAs that have been described as endogenous targets of alternative NMD pathways that are differentially sensitive to the depletion of different EJC core proteins including RNPS1, BTZ, and UPF3 (Chan et al., 2007, Chan et al., 2009, Gehring et al., 2005, Wang et al., 2014). One hypothesis to explain this differential sensitivity posits that the subunit composition of EJCs may differ on these transcripts. However, we identified high-confidence binding sites on more than 100 exons belonging to different mRNA targets of distinct NMD pathways (Table S5). For instance, the *SC35* mRNA described to be BTZ-sensitive, but RNPS1-insensitive, has binding sites for both proteins (Figure 5C). In addition, the *EPAS1* mRNA that was mainly upregulated after RNPS1 depletion (Gehring et al., 2005) shows high BTZ marks in our iCLIP data (Figure S4). Thus, differences of EJC composition do not explain the previously noted differential cofactor requirements for branch-specific NMD of such transcripts. Therefore, we hypothesize that posttranslational modifications and/or additional subunits are involved, although we cannot formally exclude that differences between the different strains of HeLa cells and growth conditions may have contributed.

An important finding of this work is that most exon junctions are bound by all four tested EJC proteins (as shown by the similar binding pattern in the heatmaps for the EJC components in Figure 4B), although some mRNAs are more prevalent in the BTZ and RNPS1 iCLIP data sets and were confirmed by RIP experiments (Figure S5). Interestingly, we observe binding of individual EJC subunits, in particular RNPS1, to non-EJC sites. For RNPS1, binding to non-EJC sites is indicated by the motif analysis because, in contrast to the other EJC components, RNPS1 bound fragments show a weaker enrichment for the splice site donor motif when the 20 top motifs are considered (see Table S2 and Figure 2D). Moreover, RNPS1 binding is less restricted to the 3′ end of exons when compared to the other three EJC components (see Figures 4A and 4B). This finding is likely explained by the known non-EJC functions of RNPS1 (Mayeda et al., 1999, Michelle et al., 2012, Murachelli et al., 2012).

In conclusion, the analysis of high-confidence EJC binding sites reveals BTZ as a reliable marker for bona fide EJCs on a transcriptome-wide level (Figure 4E).

### The EJC Signal Is Significantly Increased on Alternatively Spliced Exons

We therefore focused on those 2,194 mRNAs with particularly strong BTZ signals (see Figure 3). Because the GO terms for RNA processing, cell cycle, and chromosome organization in RNAs with strong BTZ signal (Figure 3C) were recently reported to be highly enriched for alternative splicing events (Pimentel et al., 2014), we next set out to analyze the relationship between alternative splicing and EJC deposition. Notably, this set of mRNAs shows strongly increased BTZ binding to variant and alternatively spliced exons when compared to all mRNAs (Figures 5A and S6A). Moreover, the mRNAs with strong BTZ occupancy are enriched for GO terms to be alternatively spliced (1,230/2,194) when compared to all mRNAs (7,458/19,113, p = 6.7 × 10^−72^). These findings indicate that BTZ binding, and by implication the recruitment of EJCs (see above), plays a particularly important role for alternatively spliced mRNAs.

The enrichment of BTZ binding on alternatively spliced exons is exemplified by *SRSF2* (*SC35*). This example also highlights the importance of using libraries with a high level of complexity and deep coverage of reads for the reliable detection of rare interactions between a RBP and its binding transcripts in iCLIP analyses (Sims et al., 2014), such as those occurring on alternatively spliced exons of NMD-sensitive mRNA isoforms. With the highly complex BTZ iCLIP data set, we detect not only the interactions on the abundant exons, but also disproportionately strong signals on low-abundance exons. We identify several hundred of low-abundance exons with an EJC signal that is at least twice as strong as the RNA-seq (Table 1), such as the exons of the rare NMD-sensitive *SC35C* isoform (see Figure 5B, red box highlighting exon 3), indicating that the EJC preferentially binds the variant exon of this transcript. In general, NMD-sensitive transcript isoforms are rapidly degraded, thus defining a suitable class of mRNAs for an analysis of iCLIP signals on low abundance exons. In addition, there are other low-abundance mRNAs, exemplified by *GADD45A*, which are not degraded by NMD with an average RNA-seq coverage in HeLa cells below 1 count per million (CPM; Figure S6B). Nevertheless, the BTZ iCLIP signal is strong for the two internal exons in *GADD45A*, which indicates that BTZ iCLIP data can detect EJC deposition sites with great sensitivity even in mRNAs with low expression levels (see Table 1). As a control for the specificity of EJC marks, which should be only present on mRNAs transcribed from intron containing genes, the PTB iCLIP data were used. For example, the RNA derived from the intronless *ZXDB* gene, which shows the expected lack of EJC-signals, clearly reflects PTB binding to the 3′ UTR of this transcript (Figure S6C), possibly reflecting the role of PTB in regulating 3′ end processing (Danckwardt et al., 2011, Millevoi et al., 2009).

With these data in hand, we set out to identify exons that have previously not been annotated. In an analysis including only exons containing canonical splice sites, and binding of BTZ plus one of the other EJC proteins, we identified 32 high confidence candidate exons that were not previously annotated in the Ensembl, RefSeq, or University of California Santa Cruz (UCSC) gene databases. We validated our bioinformatical approach to find candidate exons by manually checking these 32 regions in the genome browser and thus confirmed 28/32 exons in intergenic and intronic regions (Figures 5B, 5C, and S7A–S7C; Table S6). Thus, the analysis of this “deep” BTZ iCLIP data set enabled detailed insights into expressed mRNA-isoforms and even previously unknown transcripts.

### Low EJC Occupancy of mRNAs Encoding Ribosomal Proteins

**Figure 6:**
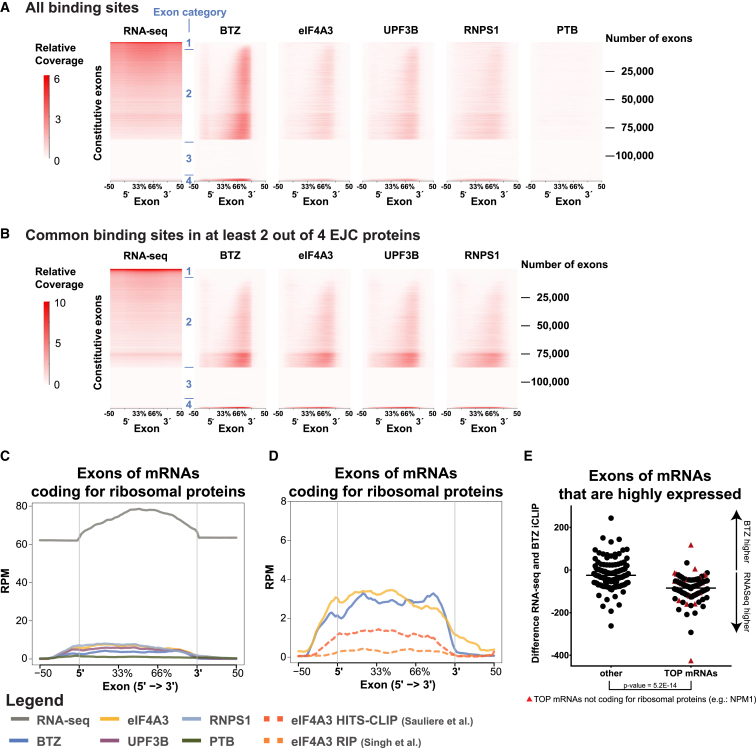
EJC Binding Is Underrepresented on mRNAs Coding for Ribosomal Proteins (A) Heatmaps of individual exons clustered by the differences between the RNA-seq and the iCLIP profile uncovered four different exon categories: (1) exons that are highly abundant and show a weak iCLIP signal; (2) exons that are expressed and harbor a corresponding EJC signal; (3) exons that are not expressed and therefore do not have an EJC signal; and (4) exons that are weakly expressed, but show enrichment of EJC binding. (B) The same clustering was performed only with reads in binding sites that were determined by at least two out of four EJC proteins. Each row of the heatmap represents one of the 123,585 constitutive exons of the human reference genome. The color key represents the signal strengths of the RNA-seq and iCLIP data. (C and D) EJC occupancy is highly diminished in mRNAs coding for ribosomal proteins. Average coverage profiles across canonical exons of ribosomal protein coding genes lack an increased EJC signal at the 3′ end of the exon for all EJC proteins (C) including published eIF4A3 data from RIP (Singh et al., 2012) and HITS-CLIP (Saulière et al., 2012) of eIF4A3 (D). The average profiles are plotted as read CPM mapped reads (RPM) calculated using peak data (C) or raw counts (D). (E) Exons of highly expressed mRNAs (>40 CPM) that do not belong to the TOP mRNA class show a higher BTZ signal analyzed by Welch two sample t test. See also Figures S4 and S7.

Ranking constitutive exons (from Figure 4B) by their EJC occupancy in relation to exon abundance (Figure 6A), we noticed four main categories: (1) exons that are highly abundant and show a weak iCLIP signal; (2) exons that are expressed and harbor a corresponding EJC signal; (3) exons that are not expressed and therefore do not have an EJC signal; and (4) exons that are weakly expressed but show enrichment of EJC binding. From the 123,585 constitutive exons in the database, 79,840 were expressed in HeLa cells above a threshold of 10 reads per exon. An EJC signal represented by BTZ was found on 60,793 of these expressed constitutive exons, if a conservative threshold of at least 10 reads per expressed exon was assigned, indicating that most exons harbor an EJC.

We further selected for bona fide EJC sites that bind at least two EJC proteins and analyzed the categories of the 123,585 constitutive exons according to their strength of BTZ/EJC binding (Figure 6B). To our surprise, more than half of the top 50 highly expressed exons with low EJC occupancy (category 1) are mRNAs coding for ribosomal proteins (Figure 6B). In general, the exons of mRNAs coding for ribosomal proteins show low BTZ/EJC binding (Figures 6C and 6D). When considering binding of the other EJC components, these can be found to bind to these exons with an equal distribution throughout their entire length, thus not representing bona fide binding as part of the EJC. We considered whether the lack of EJCs on these RNAs may be explained by the ribosomes efficiently removing EJCs because mRNAs of ribosomal proteins contain a terminal oligopyrimidine (TOP) in their 5′ UTR, which supports their efficient translation in actively proliferating cells (Meyuhas, 2000, Ruvinsky and Meyuhas, 2006). However, translation was inhibited by cycloheximide prior to iCLIP. Moreover, the average distribution of the raw reads of the two previously published transcriptome-wide studies of eIF4A3 (Saulière et al., 2012, Singh et al., 2012) also do not show a 3′ enrichment of binding on this type of exon (Figure 6D). We further considered whether the low abundance of EJC signals might be explained by the high stability of mRNAs coding for ribosomal proteins (Schwanhäusser et al., 2011). In this scenario, a high proportion of these RNA molecules may have already been translated and thus have lost their EJCs before translation has been blocked. Such a mechanism would predict that other mRNAs with long half-lives should also be found among those with a low abundance of bound EJC components. In contrast to this prediction, other stable and highly expressed mRNAs, such as actin mRNA, show strong EJC signals. We also determined whether the presence of a TOP motif might define a subset of mRNAs with low EJC abundance. While TOP mRNAs are indeed enriched in the category showing a weak iCLIP signal (Figure 6E), some exons of these mRNAs showed a canonical EJC signal (Figures S7D and S7E), indicating that the TOP motif per se does not define whether EJCs are efficiently recruited.

In conclusion, the analysis of high-confidence EJC binding sites by in vivo RNA binding studies of four different EJC proteins shows that approximately 80% of expressed constitutive exons harbor a detectable EJC signal at the canonical deposition site upstream of the 3′ end of exons. Surprisingly, exons coding for ribosomal proteins (and other TOP mRNAs) show a remarkably low occupancy of EJC components, which suggests a specific difference in RNA processing of this type of transcript. By contrast, an enrichment of the EJC signal was observed for mRNAs that derive from alternative spliced pre-mRNAs in general, including mRNAs coding for proteins associated with RNA processing in particular (McGlincy and Smith, 2008, Saltzman et al., 2008). The differential recruitment of EJCs might help the RNA-processing machinery to mark different mRNA isoforms. Moreover, the composition of the EJC is more homogeneous than previously assumed. Therefore, peripherally associated EJC proteins and/or posttranslational modifications likely play a role in determining the fate of mRNAs in alternative NMD pathways.

## Discussion

EJCs are involved in several stages of mRNA metabolism and are therefore key effectors of protein expression. In particular, EJC deposition can enhance the efficiency of NMD. Since inhibition of NMD stabilizes up to 10% of mRNAs (Mendell et al., 2004, Tani et al., 2012, Wittmann et al., 2006, Yepiskoposyan et al., 2011), one may hypothesize that the distribution and composition of EJCs across the transcriptome varies between different mRNA transcripts and in particular between targets of alternative NMD pathways (Chan et al., 2007, Chan et al., 2009, Gehring et al., 2005, Huang et al., 2011). The integrated analysis of RNA binding of four EJC components in vivo by iCLIP reported here enabled us to define high-confidence EJC binding sites defined by cooccurrence of at least two EJC subunits. The resulting landscape of bona fide EJCs across the transcriptome indicates that EJCs are recruited to most exon junctions corroborating data from IP experiments with the EJC protein eIF4A3 (Saulière et al., 2012, Singh et al., 2012).

Previously reported analyses of eIF4A3 binding indicated that 40%–50% of the binding sites of this protein are located at non-canonical regions outside of the known EJC location 20–24 nt upstream of exon-exon junctions (Saulière et al., 2012, Singh et al., 2012). Moreover, the proteomic analysis of EJCs purified from HEK293 cells showed that BTZ is substoichiometric to eIF4A3, indicating either: (1) that BTZ may not be present in all EJCs or (2) that eIF4A3 has additional functions outside the EJC core (Singh et al., 2012). Here, we provide direct evidence in favor of the latter scenario and demonstrate that fully assembled EJCs generally bind to the expected sites (see Figure 4).

Furthermore, our analyses show that the subunit composition of EJCs is surprisingly homogeneous. This result is remarkable because linking the EJC and the bound RNAs to diverse downstream cellular pathways thus appears to be independent of the composition of the core complex. These links are likely conferred by the more peripheral EJC components. Considering the structural data of the EJC-UPF3B subcomplex with short synthetic RNAs (Buchwald et al., 2010, Melero et al., 2012), one might have expected that the protein UPF3B does not bind RNA directly. By contrast, we demonstrate that all four EJC proteins directly bind to the RNA, as expected from RNA interactome data (Castello et al., 2012). Specifically, the majority of RNPS1 and UPF3B binding sites are shared by the EJC core proteins BTZ and eIF4A3. All four EJC proteins are bound to the RNA in an area of 15–30 nt upstream of the 3′ end of exons and thus map the proposed EJC binding site to a defined narrow range on a transcriptome-wide scale. This binding of multiple protein subunits of the EJC within a confined binding space may appear surprising. However, crystallography of the EJC core with BTZ and eIF4A3 revealed that both proteins could bind simultaneously in a region of six nucleotides (Andersen et al., 2006, Bono et al., 2006). Thus, there is likely sufficient space for two additional proteins. Further, our coIP experiments (Figure 1) and previous studies (Singh et al., 2012) show that the EJC is bound to the RNA as a stable complex. While PTB binds to unique sites that are mostly not shared with any of the EJC components (Table S3), eIF4A3 and UPF3B share 93% and 88% of the binding sites. Similarly, BTZ and RNPS1 accounted for fewer unique peaks (54% and 39%) compared to PTB. Importantly, unique peaks for BTZ correspond to classical EJC positions, reflecting the depth of the BTZ library. This finding is consistent with data showing that BTZ and the other EJC core factors can associate with pre-mRNAs in the nucleus (Björk et al., 2015), although BTZ is also abundant in the cytoplasm (Gehring et al., 2009a).

We suggest BTZ to serve a particularly important role as an EJC protein because: (1) it is bound almost exclusively to the canonical EJC deposition site and (2) it is present at more than 90% of the high-confidence EJC binding sites (see Table S3). As previously reported, BTZ can trigger important downstream pathways such as NMD (Gehring et al., 2005) and can stimulate translation (Chazal et al., 2013). In conclusion, our integrated approach to determine the transcriptome-wide binding of four EJC subunits identified high-confidence EJC deposition sites across the transcriptome. We propose that fully assembled EJCs are restricted to the canonical deposition sites at the 3′ end of mRNA exons in the majority of cases (see model in Figure 4E).

### EJCs Are Enriched at Alternatively Spliced mRNAs and Underrepresented on mRNAs Encoding Ribosomal Proteins and Other TOP mRNAs

It is remarkable that RNAs with strong EJC signals are enriched for alternatively spliced exons. Previous studies noted preferential binding of EJC proteins to transcripts coding for RNA processing proteins (Saulière et al., 2012, Singh et al., 2012, Wang et al., 2014), a subgroup of mRNAs that are frequently alternatively spliced (McGlincy and Smith, 2008, Pimentel et al., 2014, Saltzman et al., 2008). In contrast to earlier studies, we directly show that the enrichment of EJCs on alternatively spliced mRNAs is mainly due to the increased binding of the EJC to the alternatively spliced and variant, but not to the constitutive exons (Figure 5A).

Why may alternatively spliced mRNAs display increased EJC occupancy? Although PTCs introduced by alternative splicing could prevent the ribosome from interacting with downstream EJCs, this explanation is unlikely because translation was inhibited resulting in an equal distribution of EJCs across the transcripts (Figure S7F). Potentially, proteins involved in alternative splicing affect the splicing apparatus in a way that promotes EJC deposition.

By contrast to alternatively spliced exons, we found that mRNAs bearing the TOP motif at their 5′ ends display strongly reduced EJC binding at most exons across all sites without bias for the 5′ exon (Figures 6C, 6D, S7D, and S7E). This feature of TOP mRNAs does not appear to be governed by their typically long half-life or their high rate of translation (Schwanhäusser et al., 2011) because we found other long-lived RNAs and other particularly actively translated RNAs to be well covered by EJCs. Moreover, some individual exons of TOP RNAs display a similar EJC density as other constitutively spliced mRNAs (Figure S7D).

## Experimental Procedures

### Stable HeLa Cell Lines

Stable and inducible HeLa cell lines expressing GFP-tagged proteins at a level comparable to the endogenous counterparts were generated (see the Supplemental Experimental Procedures).

### NMD Efficiency Assay

NMD efficiency in HeLa cells was measured by a chemiluminescence NMD reporter (Figure S1A; Boelz et al., 2006).

### siRNA Knockdown and Complementation Assay

The siRNAs targeted the 3′ UTR of the endogenous EJC factors. The depletion of endogenous proteins and induction of recombinant proteins were monitored by immunoblotting and qRT-PCR (see the Supplemental Experimental Procedures; see Table S7 for siRNA and primer sequences).

### Immunoblots

Immunoblot conditions (e.g., antibodies) are provided in the Supplemental Experimental Procedures and in Table S7.

### iCLIP

Prior to iCLIP, HeLa cells were treated with 100 μg/ml cycloheximide for 1 hr to inhibit translation. For this study, the original iCLIP protocol (König et al., 2010) was used with some modifications (see the Supplemental Experimental Procedures).

### RIP

RIP followed the IP protocol for iCLIP, but omitted the UV crosslinking and the steps of RNA and protein digestion (see the Supplemental Experimental Procedures).

### RNA-Seq

RNA was isolated with TRIzol from 50 μl of the cell lysate of three biological replicates used for iCLIP experiments. Total RNA (1 μg) was diluted to a final volume of 8 μl with H2O, mixed with 1 μl of 10× reaction buffer and 1 μl of DNase I (1 U/μl) and incubated for 15 min at 37°C. RNA was purified according to the RNeasy Kit and rRNA depleted. Strand specific libraries were prepared and sequenced paired-end with 50 bp on an Illumina HiSeq2000.

### Mapping and Counting of Reads

The iCLIP and RNA-seq reads were mapped to the human reference genome (assembly GRCh37, as provided by Ensembl 75) and an exon junction database with an overhang of 99 nt using STAR v2.3.0 (Dobin et al., 2013). A total mismatch rate of 2 was allowed (outFilterMismatchNmax 3 and outFilterMismatchNoverLmax 0.12). If reads map equally well to two different locations, an alignment was randomly selected (default in STAR). iCLIP and RNA-seq reads were counted for each gene in GRCh37 with HTSeq (v0.6.1) and python (v2.7.5) using the sorted and random barcode evaluated reads.

### Genome Browser

The iCLIP reads were visualized in IGV (Robinson et al., 2011, Thorvaldsdóttir et al., 2013), together with literature data of eIF4A3 HITS-CLIP (Saulière et al., 2012) and RIP (Singh et al., 2012). RIP data (accession number: SRX189574) was converted to human genome 19 with the liftOver tool (UC Santa Cruz).

### Peak Analysis

To identify peaks, the iCount algorithm was applied (König et al., 2010, Sugimoto et al., 2012), together with our improved iCLIP analysis tools (Hauer et al., 2015). We used a flank size of 15 nt, 100 random permutations, and an FDR of <5% to identify read clusters (Yeo et al., 2009).

The enrichment factors of iCLIP peaks in the ORF and the UTRs were calculated by dividing the number of peaks by the cumulative length of those regions.

For high-confidence EJC binding site analysis, peaks that had at least an overlap of 1 nt between two different iCLIP experiments were merged into a single peak and filtered by GFP reads (see Supplemental Experimental Procedures).

### Motif Analysis

Genomic segments next to the detected iCLIP peaks were scanned for all possible 5mer. All occurring 5mers 40 nt up- and downstream of a peak midpoint were counted. As for the detection of peaks, reference data were generated by randomly shuffling the iCLIP positions 100 times within the corresponding genomic segments. The positional distribution was normalized by the mean random score of the detected 5mers in a region 100 nt up- and downstream of the peak. The 5mer is centered at the reported positions.

### Distribution of EJCs

The distribution of EJCs over the whole length of different mRNAs and exons was calculated using the ngs.plot software (Shen et al., 2014) with Ensembl 75 annotation. To visualize the differences between RNA-seq and EJC iCLIP signals, the exons were ranked with the parameter “-GO diff”.

### Candidate Exons

We searched for EJC binding sites that: (1) did not map to annotated exons (Ensembl 75, RefSeq, and UCSC databases); (2) had a BTZ signal of >2 reads per million (RPM) that was confirmed by at least one other overlapping EJC protein; and (3) contained the canonical splice site donor motif AGGTAAG or AGGTGAG in close proximity (a maximum of 25 nt).

### Differential Binding and GO Enrichment

Raw counts were used to analyze differential binding of RBPs to mRNAs by edgeR (v3.8.5) with a Benjamini-Hochberg calculated adjusted p value < 0.05 (Robinson et al., 2010). The enriched ontology terms were determined using the Database for Annotation, Visualization, and Integrated Discovery with whole genome background as default (DAVID, v6.7; Huang et al., 2009).

## Author Contributions

Conceptualization, C.H., M.W.H., and A.E.K.; Methodology, C.H. and A-.M.A.; Software and Formal Analysis, C.H., T.S., and T.C.; Visualization and Validation, C.H.; Investigation, C.H., J.S., and I.H.; Writing - Original Draft, C.H.; and Writing - Review & Editing, M.W.H. and A.E.K.

## Figures and Tables

**Table 1:** Binding of High-Confidence EJCs Identifies Low Abundance Exons and Previously Non-annotated Exons

Exon Type	Number of Exons with Signal of BTZ and One Other EJC Protein >1 RPM and RNA-Seq <0.5 RPM	% of All Annotated Exons
altBoth	208	4.8
altDonor	415	3.7
altAcceptor	668	4.0
Variant	935	1.8
Constitutive	2,904	2.3

	**Number of Non-annotated Exons with Signal of BTZ >2 RPM and One Other EJC Protein and Canonical Splice Site Donor Motif**	**Validated**

Candidate	32	28 (90%)

## Accession Numbers

The accession number for the FASTQ files of iCLIP and RNA-seq libraries reported in this paper is ArrayExpress: E-MTAB-4215.

## Supplemental Information

Document S1. Supplemental Experimental Procedures, Figures S1–S7, and Tables S1–S7

Document S2. Article plus Supplemental Information
