# Supplementary material for: Exon Junction Complexes Show a Distributional Bias toward Alternatively Spliced mRNAs and against mRNAs Coding for Ribosomal Proteins
Source: Cell Rep. 2016 Jul 28;16(6):1588–603. doi: 10.1016/j.celrep.2016.06.096 (PMC4978704; doi:10.1016/j.celrep.2016.06.096)
Supplement: Document S1. Supplemental Experimental Procedures, Figures S1–S7, and Tables S1–S7 [file mmc1.pdf]

Cell Reports, Volume *16*

Supplemental Information

Exon Junction Complexes Show a Distributional Bias toward Alternatively Spliced mRNAs and against mRNAs Coding for Ribosomal Proteins

Christian Hauer, Jana Sieber, Thomas Schwarzl, Ina Hollerer, Tomaz Curk, Anne-Marie Alleaume, Matthias W. Hentze, and Andreas E. Kulozik

Supplemental Figures

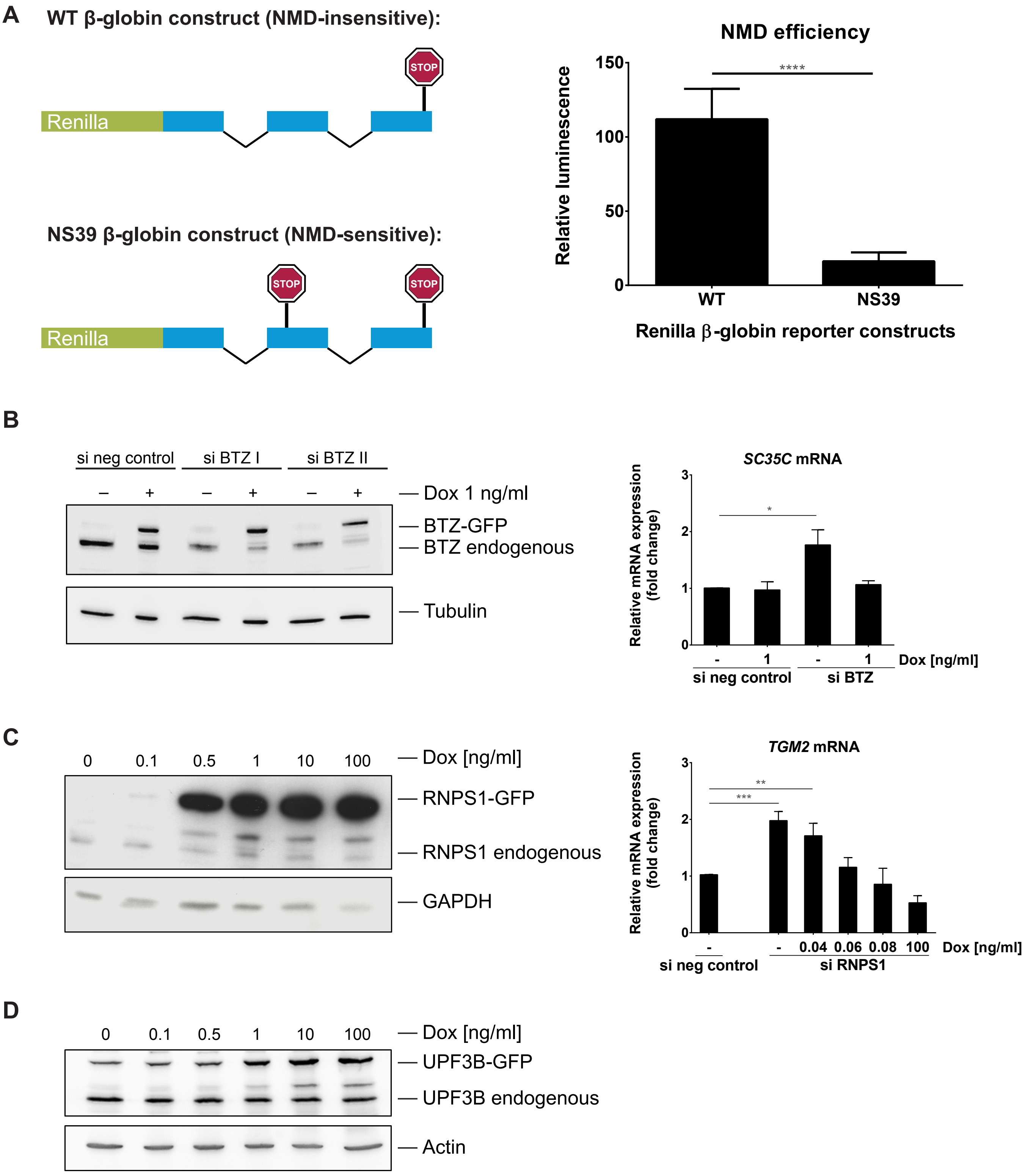

Figure S1. Related to Figure 1: Validation of HeLa cell lines. (a) Reporter constructs to measure the NMD efficiency of HeLa host cell line. The nonsense-mediated mRNA decay (NMD) pathway is fully functional in the HeLa cells used for iCLIP analyses. Error bars represent standard deviation and ****p-value < 0.0001 was calculated by two-tailed Student´s t-test, n = 3. (b-d) Validation of Hela cell lines with stably integrated fusion genes coding for proteins of (b) BTZGFP, (c) RNPS1-GFP, (d) UPF3B-GFP. The siRNA rescue experiments were only conducted for eIF4A3, BTZ and RNPS1. Error bars represent standard error of the mean and p-values were calculated by one-way ANOVA with Dunnett´s multiple comparison test; *p-value<0.05, **p-value<0.01, ***p-value<0.001 with n=3–5 independent biological experiments.Dox=doxycycline.

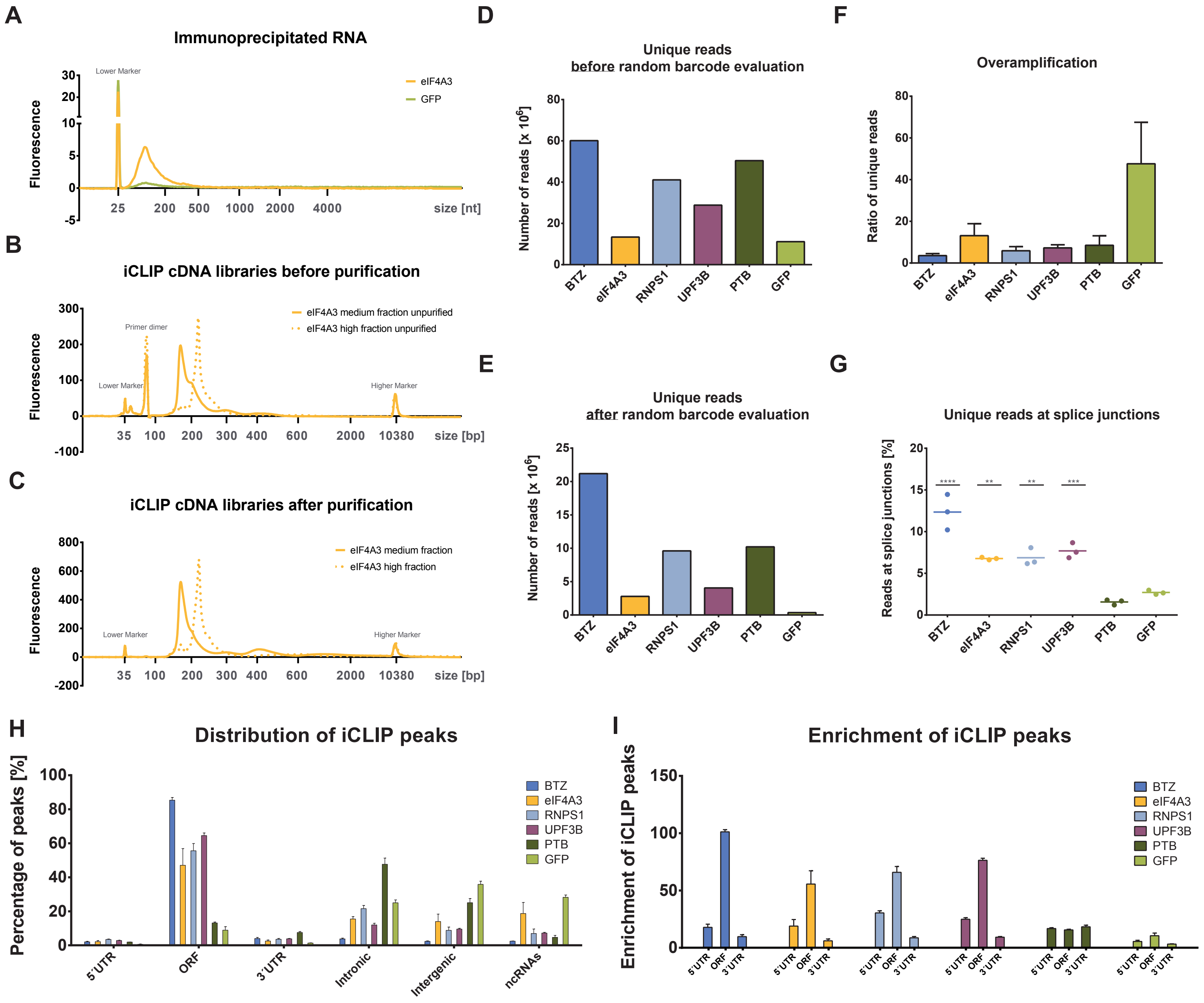

Figure S2. Related to Figure 2: Quality control of iCLIP libraries. (a-c) Size distributions during iCLIP. (a) Size distribution of co-immunoprecipitated RNA fragments after pull-down of eIF4A3-GFP and, as a control, of GFP. (b) After reverse transcription a cDNA size range between 150–300 bp was achieved and (c) primer dimers were successfully removed prior to sequencing. (d-f) iCLIP libraries of RPBs show low overamplification rates. The number of uniquely mapped reads was calculated as the sum of three biologically independent experiments before (d) and (e) after random barcode evaluation. (f) The overamplification rate was determined by the ratio of the uniquely mapped reads before and after random barcode evaluation for each replicate. The low number of uniquely mapped reads after random barcode evaluation and the high overamplification rate for GFP libraries indicated a low complexity of these libraries and validated GFP as an appropriate background control. (e) The four EJC proteins, but not PTB, were significantly enriched at the exon-exon boundaries in comparison to the GFP background control. Horizontal lines represent the mean of n = 3 independent biological experiments and the p-values compared to GFP were calculated by one-way ANOVA with Dunnett´s multiple comparison test; **p-value < 0.01, ***p-value < 0.001, ****p-value < 0.0001.
h) Distribution of peaksof all iCLIP datasets in % confirmed that EJC components bind predominantly in the ORF. This graph does not reflect the different library sizes and thus, in particular, the low GFP counts have to be taken into account. The sum of GFP reads after random barcode evaluation from the three replicates was 10-100 times lower compared to EJC and PTB reads (see panel E and Table S1). (f) Enrichment of iCLIP peaks as in Figure 2 with GFP included. Error bars represent the standard error of the mean.

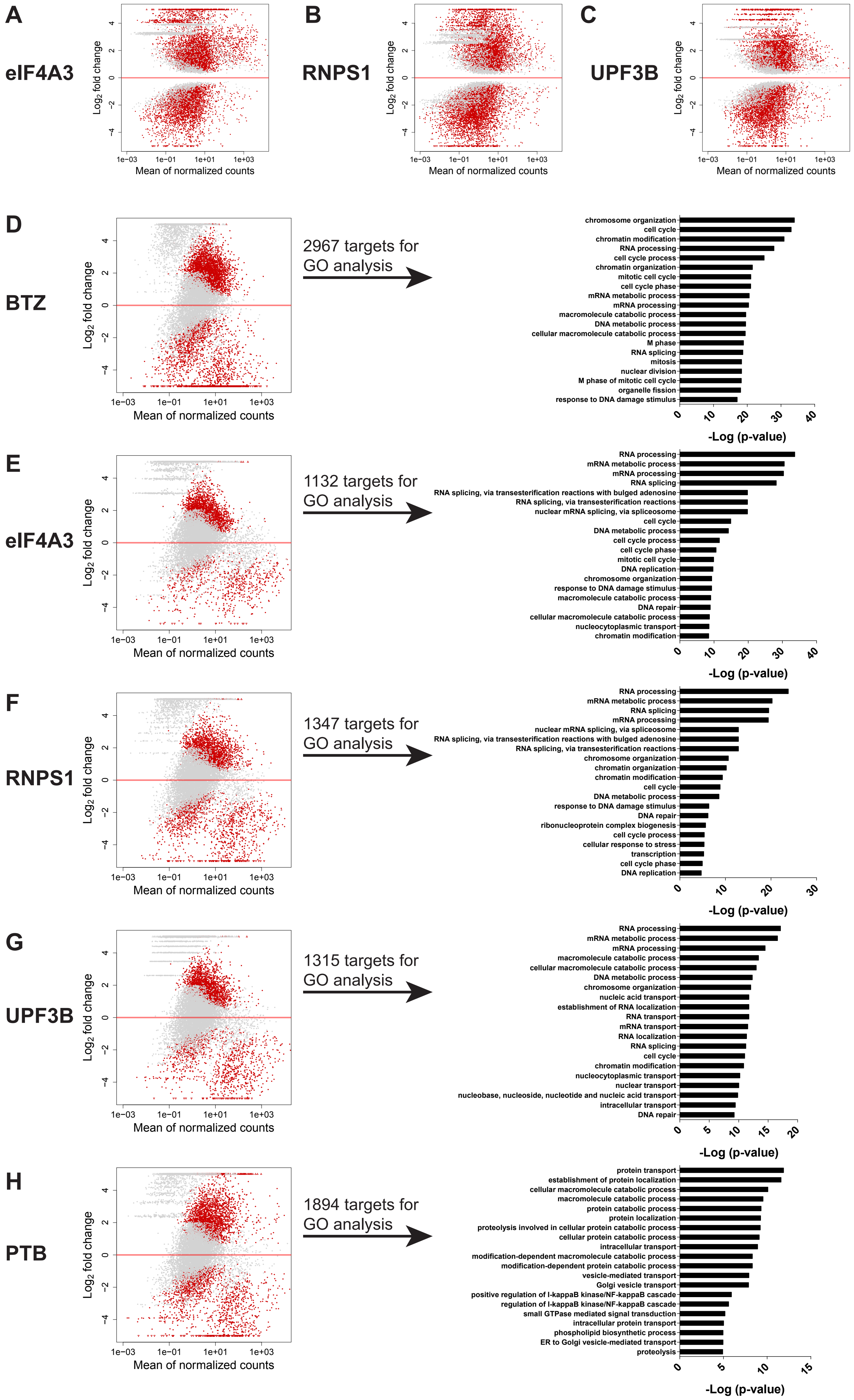
A
eIF4A3
B
RNPS1
C
UPF3B
Log2 fold change
Mean of normalized counts
D
BTZ
2967 targets for GO analysis
chromosome organization
cell cycle
chromatin modification
RNA processing
cell cycle process
chromatin organization
mitotic cell cycle
cell cycle phase
mRNA metabolic process
mRNA processing
macromolecule catabolic process
DNA metabolic process
cellular macromolecule catabolic process
M phase
RNA splicing
mitosis
nuclear division
M phase of mitotic cell cycle
organelle fission
response to DNA damage stimulus
-Log (p-value)
E
eIF4A3
1132 targets for GO analysis
RNA processing
mRNA metabolic process
mRNA processing
RNA splicing
RNA splicing, via transesterification reactions with bulged adenosine
RNA splicing, via transesterification reactions
nuclear mRNA splicing, via spliceosome
cell cycle
DNA metabolic process
cell cycle process
cell cycle phase
mitotic cell cycle
DNA replication
chromosome organization
response to DNA damage stimulus
macromolecule catabolic process
DNA repair
cellular macromolecule catabolic process
nucleocytoplasmic transport
chromatin modification
F
RNPS1
1347 targets for GO analysis
RNA processing
mRNA metabolic process
RNA splicing
mRNA processing
nuclear mRNA splicing, via spliceosome
RNA splicing, via transesterification reactions with bulged adenosine
RNA splicing, via transesterification reactions
chromosome organization
chromatin organization
chromatin modification
cell cycle
DNA metabolic process
response to DNA damage stimulus
DNA repair
ribonucleoprotein complex biogenesis
cell cycle process
cellular response to stress
transcription
cell cycle phase
DNA replication
G
UPF3B
1315 targets for GO analysis
RNA processing
mRNA metabolic process
mRNA processing
macromolecule catabolic process
cellular macromolecule catabolic process
DNA metabolic process
chromosome organization
nucleic acid transport
establishment of RNA localization
RNA transport
mRNA transport
RNA localization
RNA splicing
cell cycle
chromatin modification
nucleocytoplasmic transport
nuclear transport
nucleobase, nucleoside, nucleotide and nucleic acid transport
intracellular transport
DNA repair
H
PTB
1894 targets for GO analysis
protein transport
establishment of protein localization
cellular macromolecule catabolic process
macromolecule catabolic process
protein catabolic process
protein localization
proteolysis involved in cellular protein catabolic process
cellular protein catabolic process
intracellular transport
modification-dependent macromolecule catabolic process
modification-dependent protein catabolic process
vesicle-mediated transport
Golgi vesicle transport
positive regulation of I-kappaB kinase/NF-kappaB cascade
regulation of I-kappaB kinase/NF-kappaB cascade
small GTPase mediated signal transduction
intracellular protein transport
phospholipid biosynthetic process
ER to Golgi vesicle-mediated transport
proteolysis

Figure S3. Related to Figure 3: Differential analysis of EJC data compared to PTB and GFP iCLIP data. The red dots in the plots show signigicant mRNA targets either up- or downregulated after differential analysis using edgeR controlled by Benjamini-Hochberg procedure with a FDR < 0.05. (a-c) Analog to Figure 4A for (a) eIF4A3, (b) RNPS1 and (c) UPF3B. (d-h) Targets that are above the red line have a higher signal for the EJC (or PTB) component compared to GFP. These targets are analyzed for gene ontology (GO) enrichment for (d) BTZ, (e) eIF4A3, (f) RNPS1, (g) UPF3B and (h) PTB.

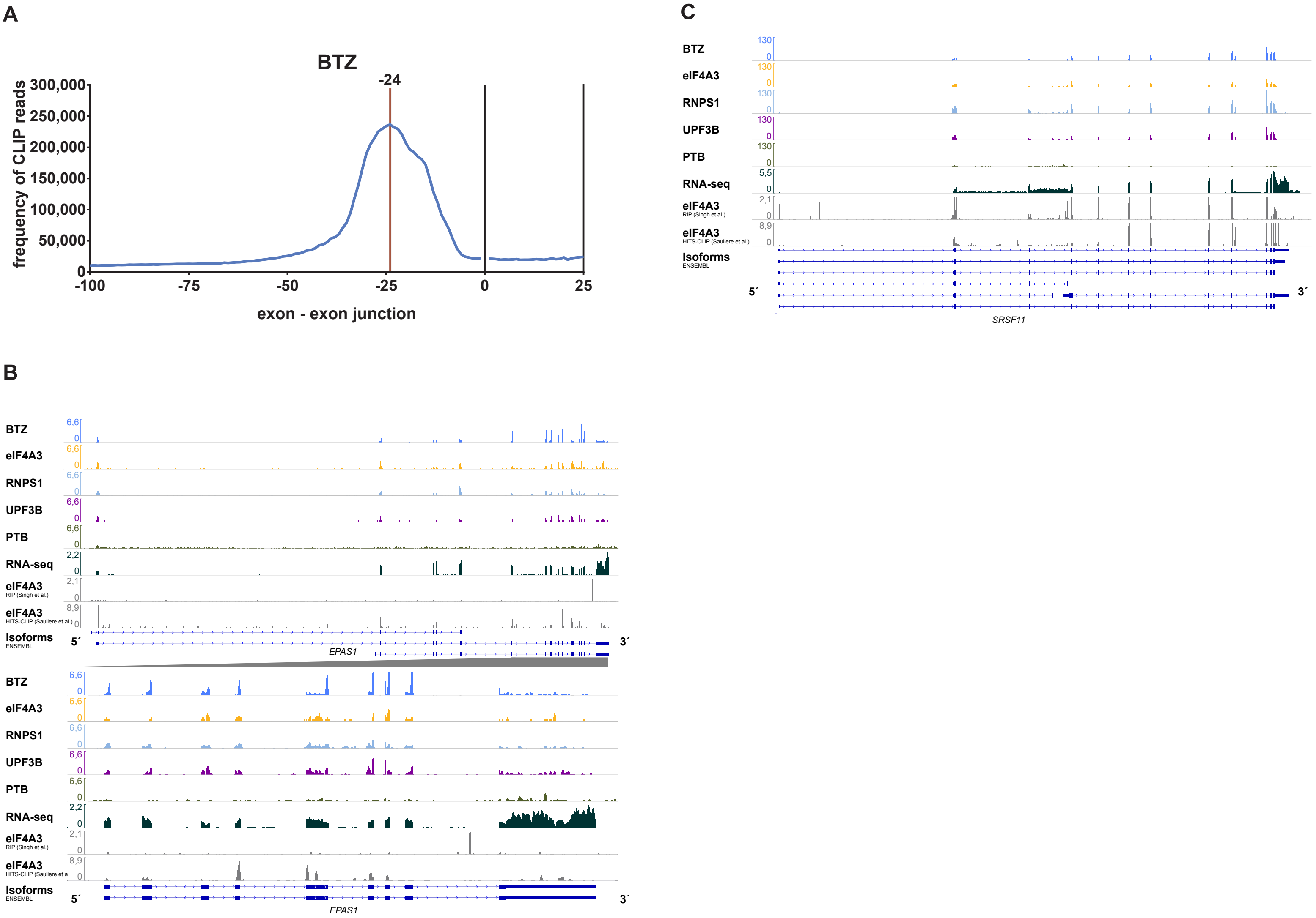

Figure S4. Related to Figures 4, 5, and 6: (a) Metablot of iCLIP reads of one BTZ replictate at exon-exon junctions. The vast majority of the signal clusters around the -24 nts position (red bar). (b-c) Genome browser view of mRNA targets belonging to alternative NMD pathways indicates homogenous EJC composition. (b) *EPAS1* mRNA is RNPS1 sensitive (Gehring et al., 2015), whereas (c) *SRSF11* mRNA is UPF3B sensitive (Chan et al., 2007). The track range displays count per million (CPM) and was adjusted to the highest iCLIP signal obtained in each genome browser view. The signals of the RNA-Seq and literature data were not adjusted. Literature data were obtained from RIP (Singh et al., 2012) and HITS-CLIP (Sauliere et al., 2012) of eIF4A3.

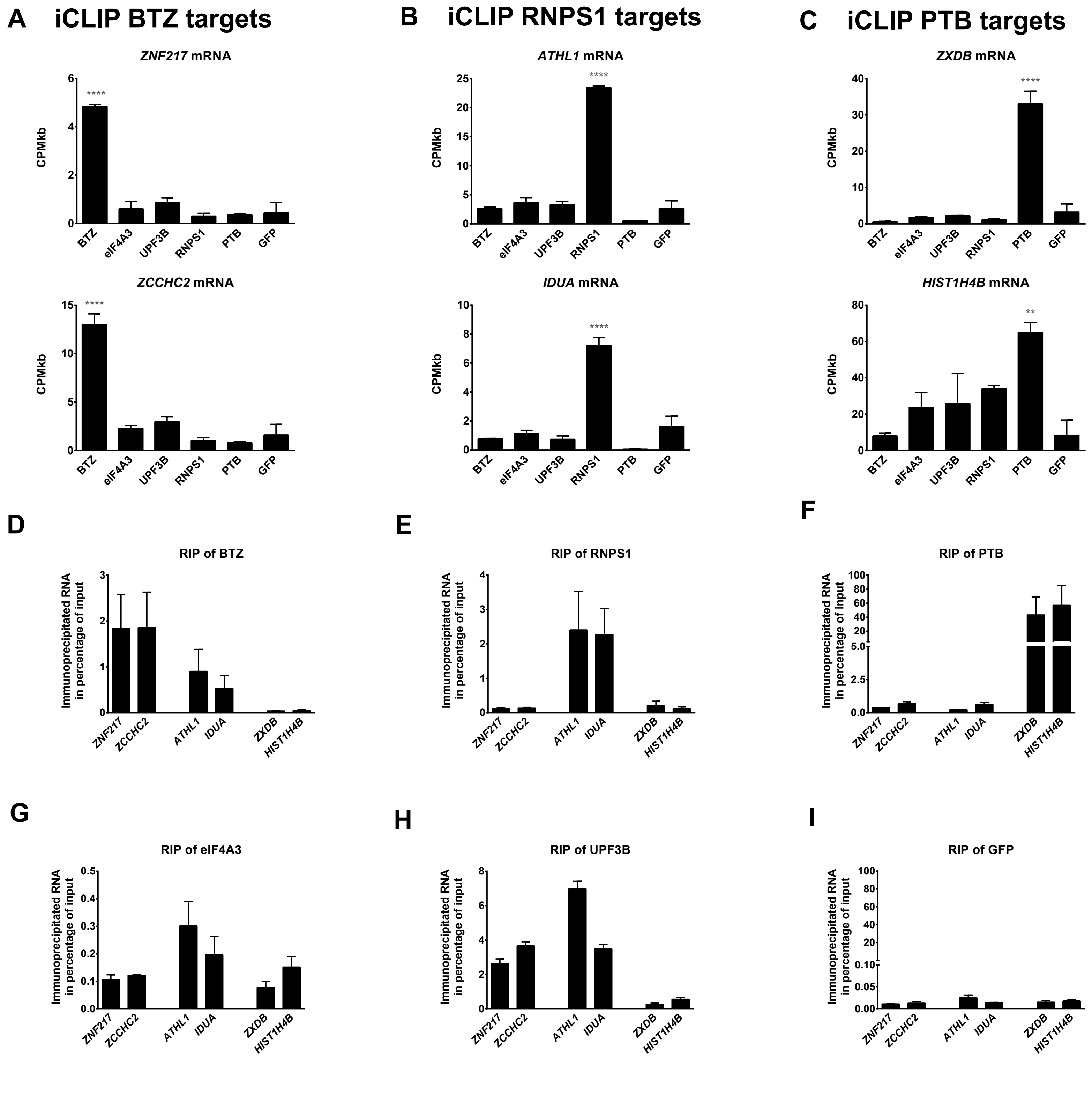

Figure S5 related to Figure 4: Selected mRNAs that were enriched for (a) BTZ, (b) RNPS1 and (c) PTB binding sites in iCLIP analysis. p-values compared to GFP were calculated by one-way ANOVA with Dunnett´s multiple comparison test; **p-value < 0.01, ****p-value <0.0001 with n = 3 independent biological experiments. CPMkb: counts per million mapped reads adjusted by gene length in kilobases. iCLIP identified targets of BTZ (*ZNF217*, *ZCCHC2*) and RNPS1 (*ATHL1*, *IDUA*) were quantified together with intronless transcripts enriched for PTB in the 3´UTR (*ZXDB*, *HIST1H4B*) using RNA immunoprecipitation (RIP) experiments (d-i). The RIP experiments of (d) BTZ, (e) RNPS1 and (f) PTB indicated distince specificities.
The RIP experiments of (g) eIF4A3 and (h) UPF3B exhibiteda similar pattern, but strong differences in the amount of co-immunoprecipitated RNA. The control immunoprecipitation of (i) GFP only resulted in background signals for all transcripts. Error bars represent standard error of the mean of n = 3 independentbiological experiments.

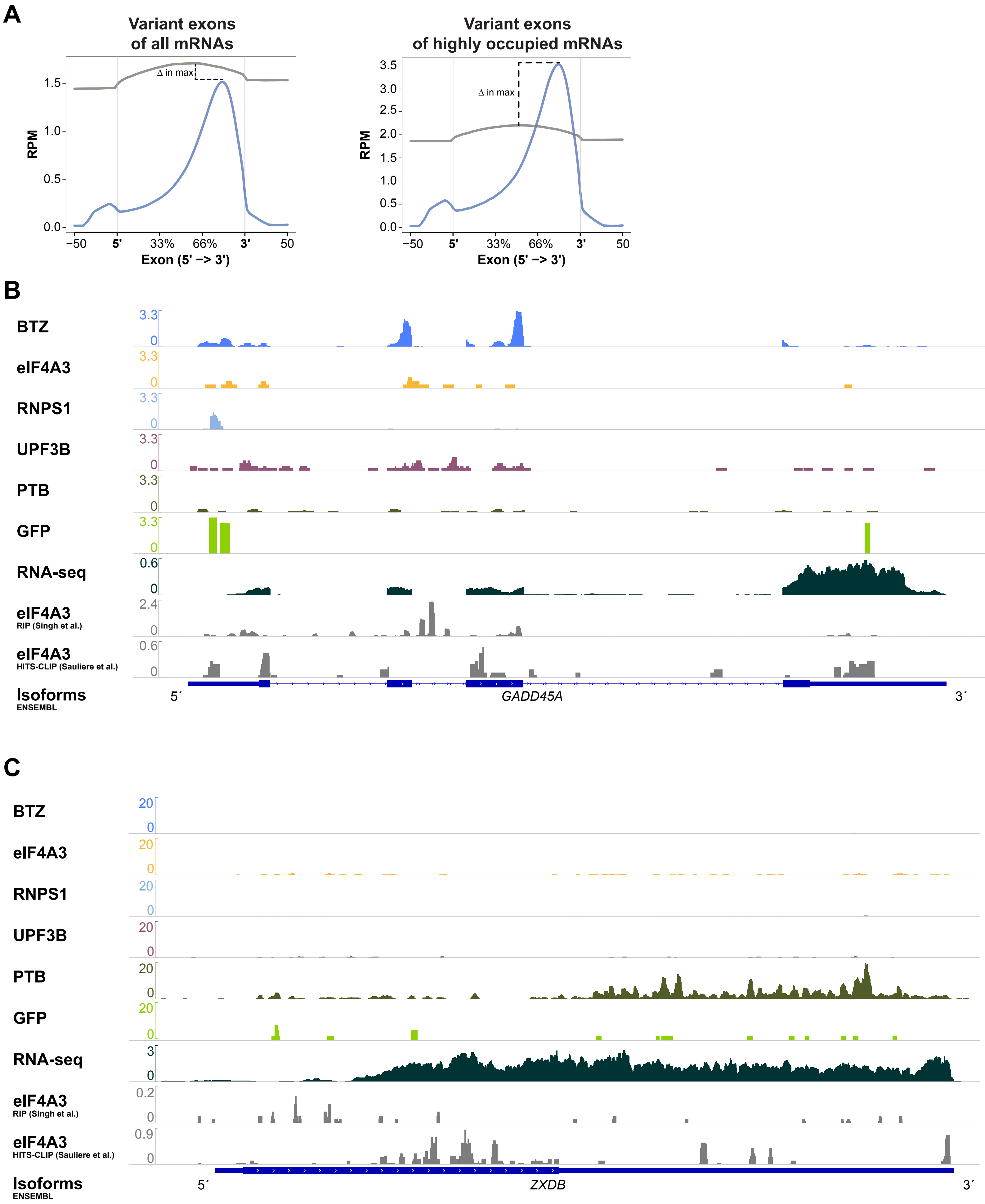

Figure S6. Related to Figure 5: Analysis of binding sites at specific exons and transcripts. (a) Scheme of how the data depicted in Figure 5A is generated. (b) Genome browser view of a low-abundance transcript, *GADD45A*, demonstrates the sensitive detection of EJC deposition sites. (c) Genome browser view on an intronless transcript, *ZXDB*, demonstrates the high degree of specificity of the iCLIP data, because only PTB and none of the EJC components were bound to the transcript. In particular, most of the PTB signal was located in the 3´UTR of this transcript. The track range displays count per million (CPM) and was adjusted to the highest iCLIP signal obtained in each genome browser view. The signals of the RNA-Seq and literature data were not adjusted. Literature data were obtained from RIP (Singh et al., 2012) and HITS-CLIP (Sauliere et al., 2012) of eIF4A3.

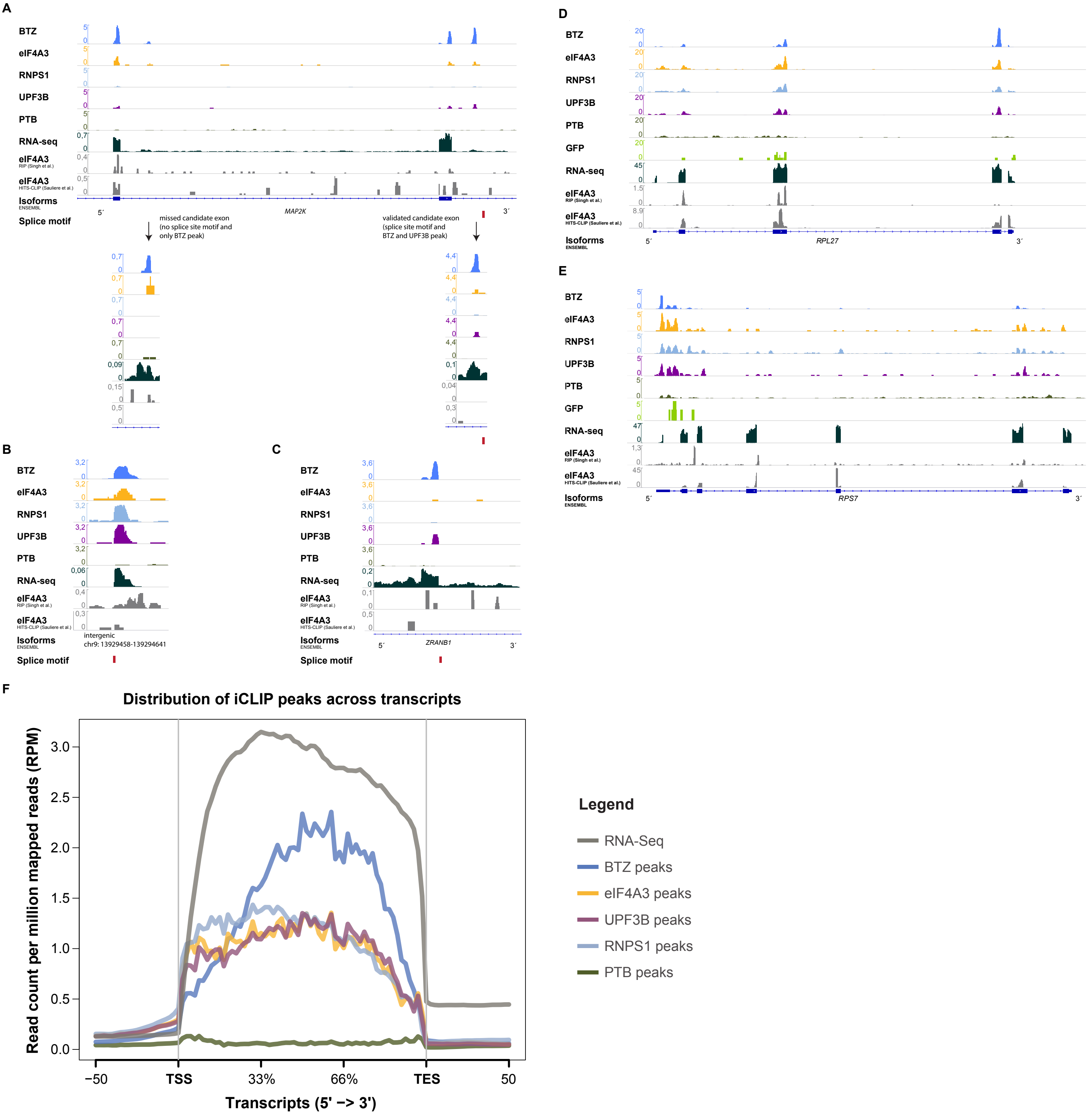

Figure S7. Related to Figure 4, 5 and 6: (a-c) Genome browser view of candidate exons. (a) *MAP2K4* mRNA with 4 BTZ peaks. Only two peaks overlap with annotated exons. The other two peaks map to intronic regions that are enriched in the RNA-seq compared to the surrounding (see zoom in). Our approach only detects the downstream region as a candidate exon due to the peak height and the splice site donor motif in the correct position. Further candidate exons were found in intergenic (b) and (c) intronic regions in the transcriptome (see also Table S5).
(d-e) Genome browser view of mRNAs coding for ribosomal proteins indicates differential binding of EJC components. (d) Internal exons of the mRNA coding for RPL27 possessed an EJC signal, whereas (b) internal exons of the mRNA coding for RBS7 did not exhibit a signal for EJC deposition.
The track range displays count per million (CPM) and was adjusted to the highest iCLIP signal obtained in each genome browser view. The signals of the RNA-Seq and literature data were not adjusted. Literature data were obtained from RIP (Singh et al., 2012) and HITS-CLIP (Sauliere et al., 2012) of eIF4A3.
(f) The RNA-seq and the EJC iCLIP datasets cover the entire gene body and thus EJC binding is not biased neither towards the 5´ nor the 3´ end of transcripts. Average profile of reads covering protein-coding mRNAs from the transcription start site (TSS) until the transcription end site (TES) are plotted as read count per million mapped reads (RPM) using ngsplot software (Shen et al., 2014).

Supplemental Tables

Table S1: Statistics for iCLIP libraries. Deletion and mismatch rates were calculated per base. Last column represents the final quality-controlled reads. Related to Figure 2.

	Rep.	Adapter removal		Mapped reads						Barcode
		Input reads	Output reads	Unique	Unique ratio	Mismatches	Deletions	Multiple loci	Too many loci	Evaluated reads
	1	17,583,508	16,523,573	7,143,889	43.23%	0.37%	0.08%	39.68%	14.70%	300,471
**eIF4A3**	2	29,781,052	28,329,506	9,190,552	32.44%	0.45%	0.07%	50.27%	7.22%	2,230,337
	3	9,832,432	9,349,933	2,950,133	31.55%	0.57%	0.04%	43.84%	16.51%	256,743
	1	35,130,380	33,715,899	19,720,084	58.49%	0.43%	0.04%	35.94%	2.49%	3,871,095
**BTZ**	2	39,043,789	37,127,273	23,722,280	63.89%	0.30%	0.05%	27.85%	2.55%	12,907,006
	3	30,111,493	28,639,999	16,662,127	58.18%	0.42%	0.03%	29.17%	5.26%	4,404,326
	1	36,757,266	34,628,259	19,155,698	55.32%	0.50%	0.06%	33.15%	8.44%	3,582,604
**RNPS1**	2	36,708,404	34,475,502	14,520,941	42.12%	0.45%	0.08%	36.83%	9.33%	5,246,574
	3	20,049,932	19,156,642	7,415,654	38.71%	0.60%	0.04%	32.87%	14.91%	776,597
	1	31,038,246	29,478,768	11,898,252	40.36%	0.54%	0.06%	50.09%	6.35%	1,247,946
**UPF3B**	2	28,272,349	26,188,344	10,547,640	40.28%	0.53%	0.07%	49.91%	6.26%	1,350,142
	3	24,446,683	23,362,640	6,414,348	27.46%	0.56%	0.06%	53.35%	4.79%	1,455,514
	1	23,501,063	22,691,882	13,302,382	58.62%	0.47%	0.11%	30.33%	3.78%	6,933,311
**PTB**	2	19,356,590	18,688,782	11,147,430	59.65%	0.51%	0.05%	30.25%	3.94%	1,777,489
	3	50,139,813	47,293,611	25,973,284	54.92%	0.33%	0.04%	32.87%	4.73%	1,501,573
	1	21,484,620	17,976,060	3,489,070	19.41%	0.54%	0.08%	63.14%	8.23%	43,702
**GFP**	2	9,925,921	9,539,105	2,389,971	25.05%	0.68%	0.04%	44.93%	15.59%	211,148
	3	25,181,318	23,847,567	5,280,032	22.14%	0.73%	0.03%	42.94%	8.09%	102,122

Table S2: The 20 most enriched 5mers for the iCLIP datasets performed in triplicate in descending order. The colored 5mers contain a GT-motif. Related to Figure 2.

	eIF4A3		BTZ		RNPS1		UPF3B		PTB		GFP	
	**5mer**	**z-score**	**5mer**	**z-score**	**5mer**	**z-score**	**5mer**	**z-score**	**5mer**	**z-score**	**5mer**	**z-score**
**1**	AGGTA	200	AGGTA	908	GAAGA	322	GAAGA	189	TCTTC	401	GGGGG	103
**2**	GGTAA	151	GGTAA	803	AGAAG	268	AGGTA	166	CTTCC	380	CCCCC	87
**3**	GTAAG	144	GTAAG	713	AAGAA	250	GGTAA	165	CTTCT	373	AAAAA	74
**4**	GTGAG	143	GTGAG	526	GAGGA	234	AGAAG	154	TCTCT	360	TTTTT	56
**5**	GGTGA	136	CAGGT	510	AGGAG	209	AAGAA	139	CTCTT	360	GGCGG	44
**6**	CGGCG	122	AAGGT	486	GCGGC	204	GTAAG	137	TCTCC	358	CGGCG	40
**7**	CAGGT	122	GGTGA	455	GGCGG	183	GTGAG	122	TCCTC	357	GCGGG	40
**8**	GCGGC	121	GAAGA	354	CGGCG	182	GGTGA	115	CTCTC	350	GCGGC	36
**9**	GCCCC	108	AGGTG	331	CCCCC	177	GAGGA	105	TCCCT	348	CGGGG	35
**10**	CCGGC	108	TAAGT	312	GGAGG	175	AAGGT	100	CTTTC	346	GGGCG	30
**11**	GGCGG	107	AGAAG	292	AGGTA	171	AGGTG	93	CCTTC	345	CAGGG	28
**12**	CCCGG	106	AAGAA	279	TCCCC	164	GCGGC	93	TTCTC	343	CTCTC	27
**13**	CGGCC	106	TGAGT	254	CCCCG	164	CAGGT	89	CCTCT	339	CCCCG	27
**14**	CCCCG	103	GGTAT	243	GCCCC	156	GGCGG	88	TTCCC	326	TCTCT	26
**15**	CCCCC	100	GAGGT	233	CCCGG	154	CGGCG	83	TCCTT	325	GGTCG	26
**16**	AAGGT	98	GTATG	212	AGCGG	152	GGAGG	74	TCCCC	323	TGATC	24
**17**	AGGTG	92	CAAGG	187	CCCTC	151	AGGAG	72	TTTCC	310	GGGGC	22
**18**	CGCCG	88	GGTAG	186	CCTCC	151	AAAAA	71	TTCCT	303	GGGAG	22
**19**	GCCGG	86	GGTAC	179	GGTAA	148	CCCCG	71	CCTTT	294	CCCTC	22
**20**	GCTGC	79	AAGTA	177	AGAGG	141	GCCCC	70	TTTCT	291	CGCCC	21

Table S3: Number of peaks in iCLIP libraries. Related to Figure 2.

	BTZ	eIF4A3	RNPS1	UPF3B	PTB	Overlap
**Total Peaks**	173,944	48,584	127,642	68,146	172,175	
**after GFP filtering**	164,627	35,150	115,223	60,374	145,797	
**restricted to one RBP**	89,046	2,613	44,646	6,963	116,969	
**with PTB**						346,396
**w/o PTB, at least one EJC protein**						200,599
**w/o PTB, at least two EJC proteins**						59,521
**w/o PTB, at least two EJC proteins, protein-coding**						54,645
**BTZ + eIF4A3**						2,007
**BTZ + RNPS1**						16,690
**BTZ + UPF3B**						8,694
**eIF4A3 + RNPS1**						1,538
**eIF4A3 + UPF3B**						251
**RNPS1 + UPF3B**						3,026
**BTZ + eIF4A3 + RNPS1**						3,083
**BTZ + eIF4A3 + UPF3B**						1,644
**BTZ + RNPS1 + UPF3B**						10,699
**eIF4A3 + RNPS1 + UPF3B**						894
**BTZ + eIF4A3 + RNPS1 + UPF3B**						10,995
**high-confidence peaks with BTZ**						53,812
**high-confidence peaks w/o BTZ**						5,709

Table S4: Maximal read count per million mapped reads (RPM) value of iCLIP and RNA-seq libraries (Max) including standard error of the mean (SEM). These values correspond to average profiles of different exon types depicted in Figure 4.

	First		Terminal		Canonical		Variant		ALT acceptor		ALT donor		ALT both	
	Max	SEM	Max	SEM	Max	SEM	Max	SEM	Max	SEM	Max	SEM	Max	SEM
**RNA-seq**	1.43	0.02	2.50	0.03	1.41	0.01	1.71	0.03	2.42	0.07	2.45	0.11	4.29	0.23
**BTZ peaks**	0.93	0.01	0.61	0.01	2.51	0.02	1.52	0.03	3.13	0.07	2.35	0.06	3.07	0.15
**eIF4A3 peaks**	1.05	0.02	0.47	0.01	0.92	0.02	0.86	0.03	1.94	0.06	1.57	0.06	3.03	0.16
**UPF3B peaks**	0.79	0.01	0.41	0.01	1.07	0.02	0.75	0.02	1.63	0.06	1.40	0.06	2.29	0.17
**RNPS1 peaks**	0.80	0.01	0.50	0.01	0.64	0.01	0.57	0.01	1.29	0.04	1.20	0.04	2.42	0.11
**PTB peaks**	0.24	0.01	0.16	0.01	0.07	0.00	0.13	0.01	0.14	0.01	0.13	0.01	0.26	0.03

Table S5: EJC deposition on exons of targets belonging to alternative NMD pathways. Related to Figure 4.

chromosome	start	end	gene_ID	gene_name	transcript_ID	exon_category	start_peaks	end_peak	peak_constitution	reference of depletion	siRNA
chr17	66873660	66873820	ENSG00000141338	ABCA8	ENST00000269080	altAcceptor	66873666	66873698	BU	Chan et al., 2007	UPF3B
chr17	66878004	66878121	ENSG00000141338	ABCA8	ENST00000269080	constitutive	66878016	66878042	BU	Chan et al., 2007	UPF3B
chr2	85991114	85991305	ENSG00000168874	ATOH8	ENST00000469442	constitutive	85991243	85991304	BER	Chan et al., 2009	UPF3A and UPF3B
chr7	43843275	43843446	ENSG00000106605	BLVRA	ENST00000265523	constitutive	43843408	43843437	BU	Chan et al., 2007	UPF3B
chr6	87795968	87796152	ENSG00000135346	CGA	ENST00000369582	constitutive	87795929	87796009	BE	Chan et al., 2007	UPF3B
chr2	46609114	46609228	ENSG00000116016	EPAS1	ENST00000263734	constitutive	46609172	46609222	BER	Gehring et al., 2005	RNPS1
chr2	46574012	46574202	ENSG00000116016	EPAS1	ENST00000263734	constitutive	46574168	46574209	BR	Gehring et al., 2005	RNPS1
chr2	46587777	46587895	ENSG00000116016	EPAS1	ENST00000263734	constitutive	46587860	46587895	BR	Gehring et al., 2005	RNPS1
chr2	46602829	46602976	ENSG00000116016	EPAS1	ENST00000263734	constitutive	46602923	46602970	BR	Gehring et al., 2005	RNPS1
chr2	46603678	46603892	ENSG00000116016	EPAS1	ENST00000263734	constitutive	46603827	46603884	BR	Gehring et al., 2005	RNPS1
chr2	46605033	46605226	ENSG00000116016	EPAS1	ENST00000263734	constitutive	46605077	46605106	BR	Gehring et al., 2005	RNPS1
chr2	46605796	46605906	ENSG00000116016	EPAS1	ENST00000263734	constitutive	46605860	46605900	BR	Gehring et al., 2005	RNPS1
chr2	46608735	46608861	ENSG00000116016	EPAS1	ENST00000263734	constitutive	46608725	46608781	BR	Gehring et al., 2005	RNPS1
chr2	46605033	46605226	ENSG00000116016	EPAS1	ENST00000263734	constitutive	46605163	46605218	BU	Gehring et al., 2005	RNPS1
chr2	46609564	46609737	ENSG00000116016	EPAS1	ENST00000263734	constitutive	46609676	46609737	BU	Gehring et al., 2005	RNPS1
chr2	46607366	46607856	ENSG00000116016	EPAS1	ENST00000263734	constitutive	46607800	46607855	BUR	Gehring et al., 2005	RNPS1
chr2	46608735	46608861	ENSG00000116016	EPAS1	ENST00000263734	constitutive	46608823	46608881	BUR	Gehring et al., 2005	RNPS1
chr2	120831676	120831754	ENSG00000115109	EPB41L5	ENST00000263713	constitutive	120831727	120831743	BU	Chan et al., 2009	UPF3A and UPF3B
chr2	120849131	120849237	ENSG00000115109	EPB41L5	ENST00000263713	constitutive	120849193	120849236	BUR	Chan et al., 2009	UPF3A and UPF3B
chrX	133981413	133981505	ENSG00000156500	FAM122C	ENST00000370785	variant	133981467	133981495	BE	Chan et al., 2009	UPF3A and UPF3B
chrX	133963256	133963312	ENSG00000156500	FAM122C	ENST00000370784	constitutive	133963255	133963308	BER	Chan et al., 2009	UPF3A and UPF3B
chrX	133948801	133948879	ENSG00000156500	FAM122C	ENST00000370784	constitutive	133948818	133948879	BEUR	Chan et al., 2009	UPF3A and UPF3B
chrX	133986830	133986926	ENSG00000156500	FAM122C	ENST00000370784	variant	133986889	133986918	BR	Chan et al., 2009	UPF3A and UPF3B
chr9	74828802	74828907	ENSG00000119125	GDA	ENST00000545168	constitutive	74828871	74828899	BER	Chan et al., 2007	UPF3B
chr9	74856068	74856214	ENSG00000119125	GDA	ENST00000545168	constitutive	74856172	74856211	BER	Chan et al., 2007	UPF3B
chr9	74863188	74863322	ENSG00000119125	GDA	ENST00000475764	altDonor	74863289	74863319	BEU	Chan et al., 2007	UPF3B
chr1	158985662	158985777	ENSG00000163565	IFI16	ENST00000368131	constitutive	158985672	158985701	BR	Chan et al., 2009	UPF3A and UPF3B
chr1	159021469	159023514	ENSG00000163565	IFI16	ENST00000493884	altAcceptor	159023368	159023399	BR	Chan et al., 2009	UPF3A and UPF3B
chr15	99442706	99442850	ENSG00000140443	IGF1R	ENST00000268035	constitutive	99442799	99442844	BER	Chan et al., 2007	UPF3B
chr15	99456273	99456511	ENSG00000140443	IGF1R	ENST00000268035	constitutive	99456469	99456510	BEUR	Chan et al., 2007	UPF3B
chr15	99459193	99459360	ENSG00000140443	IGF1R	ENST00000268035	altAcceptor	99459305	99459358	BR	Chan et al., 2007	UPF3B
chr15	99459243	99459360	ENSG00000140443	IGF1R	ENST00000560144	altAcceptor	99459305	99459358	BR	Chan et al., 2007	UPF3B
chr15	99459901	99460105	ENSG00000140443	IGF1R	ENST00000268035	constitutive	99460071	99460097	BR	Chan et al., 2007	UPF3B
chr15	99482430	99482589	ENSG00000140443	IGF1R	ENST00000268035	constitutive	99482548	99482584	BR	Chan et al., 2007	UPF3B
chr15	99486152	99486281	ENSG00000140443	IGF1R	ENST00000268035	constitutive	99486196	99486212	BR	Chan et al., 2007	UPF3B
chr15	99451914	99452128	ENSG00000140443	IGF1R	ENST00000268035	constitutive	99452085	99452127	BU	Chan et al., 2007	UPF3B
chr15	99467105	99467241	ENSG00000140443	IGF1R	ENST00000268035	constitutive	99467208	99467237	BU	Chan et al., 2007	UPF3B
chr15	99486152	99486281	ENSG00000140443	IGF1R	ENST00000268035	constitutive	99486236	99486281	BU	Chan et al., 2007	UPF3B
chr15	99473464	99473534	ENSG00000140443	IGF1R	ENST00000268035	constitutive	99473476	99473569	BUR	Chan et al., 2007	UPF3B
chr15	99478545	99478655	ENSG00000140443	IGF1R	ENST00000268035	constitutive	99478608	99478651	BUR	Chan et al., 2007	UPF3B
chr15	99491803	99491937	ENSG00000140443	IGF1R	ENST00000268035	constitutive	99491802	99491828	BUR	Chan et al., 2007	UPF3B
chr15	99434554	99434866	ENSG00000140443	IGF1R	ENST00000268035	constitutive	99434776	99434790	UR	Chan et al., 2007	UPF3B
chr15	99459901	99460105	ENSG00000140443	IGF1R	ENST00000268035	constitutive	99459957	99459995	UR	Chan et al., 2007	UPF3B
chr15	99465377	99465660	ENSG00000140443	IGF1R	ENST00000268035	constitutive	99465480	99465498	UR	Chan et al., 2007	UPF3B
chr15	99478053	99478282	ENSG00000140443	IGF1R	ENST00000268035	variant	99478102	99478158	UR	Chan et al., 2007	UPF3B
chr4	57898592	57898718	ENSG00000163453	IGFBP7	ENST00000295666	constitutive	57898592	57898629	BUR	Chan et al., 2007	UPF3B
chr2	208030191	208030430	ENSG00000118263	KLF7	ENST00000426163	variant	208030199	208030232	BR	Chan et al., 2007	UPF3B
chr3	122170823	122170911	ENSG00000114030	KPNA1	ENST00000344337	constitutive	122170829	122170861	BEU	Wang et al., 2014	eIF4A3 and BTZ
chr3	122182789	122182888	ENSG00000114030	KPNA1	ENST00000344337	constitutive	122182788	122182843	BEUR	Wang et al., 2014	eIF4A3 and BTZ
chr3	122186169	122186276	ENSG00000114030	KPNA1	ENST00000344337	constitutive	122186169	122186263	BEUR	Wang et al., 2014	eIF4A3 and BTZ
chr3	122215284	122215417	ENSG00000114030	KPNA1	ENST00000344337	constitutive	122215287	122215353	BEUR	Wang et al., 2014	eIF4A3 and BTZ
chr3	122160885	122160963	ENSG00000114030	KPNA1	ENST00000344337	constitutive	122160886	122160940	BR	Wang et al., 2014	eIF4A3 and BTZ
chr3	122168421	122168584	ENSG00000114030	KPNA1	ENST00000344337	constitutive	122168428	122168463	BR	Wang et al., 2014	eIF4A3 and BTZ
chr3	122180071	122180165	ENSG00000114030	KPNA1	ENST00000344337	constitutive	122180081	122180158	BR	Wang et al., 2014	eIF4A3 and BTZ
chr9	126777377	126777804	ENSG00000106689	LHX2	ENST00000446480	altAcceptor	126777763	126777801	BU	Chan et al., 2009	UPF3A and UPF3B
chr9	126777401	126777804	ENSG00000106689	LHX2	ENST00000373615	altAcceptor	126777763	126777801	BU	Chan et al., 2009	UPF3A and UPF3B
chr18	2937690	2938035	ENSG00000101577	LPIN2	ENST00000261596	constitutive	2937703	2937731	BR	Chan et al., 2007	UPF3B
chr18	2926721	2926803	ENSG00000101577	LPIN2	ENST00000261596	constitutive	2926724	2926764	BU	Chan et al., 2007	UPF3B
chr18	2939478	2939601	ENSG00000101577	LPIN2	ENST00000261596	constitutive	2939487	2939515	BU	Chan et al., 2007	UPF3B
chr15	94927251	94927353	ENSG00000140563	MCTP2	ENST00000456504	constitutive	94927306	94927344	BE	Chan et al., 2009	UPF3A and UPF3B
chr15	95019925	95020022	ENSG00000140563	MCTP2	ENST00000456504	constitutive	95019971	95020015	BE	Chan et al., 2009	UPF3A and UPF3B
chr15	94883428	94883504	ENSG00000140563	MCTP2	ENST00000456504	constitutive	94883466	94883498	BER	Chan et al., 2009	UPF3A and UPF3B
chr15	94928652	94928754	ENSG00000140563	MCTP2	ENST00000456504	constitutive	94928708	94928753	BER	Chan et al., 2009	UPF3A and UPF3B
chr15	94983405	94983527	ENSG00000140563	MCTP2	ENST00000456504	variant	94983446	94983527	BEU	Chan et al., 2009	UPF3A and UPF3B
chr15	94841430	94841959	ENSG00000140563	MCTP2	ENST00000456504	constitutive	94841919	94841957	BEUR	Chan et al., 2009	UPF3A and UPF3B
chr15	94882519	94882661	ENSG00000140563	MCTP2	ENST00000456504	constitutive	94882622	94882659	BEUR	Chan et al., 2009	UPF3A and UPF3B
chr15	94943150	94943224	ENSG00000140563	MCTP2	ENST00000456504	constitutive	94943174	94943218	BEUR	Chan et al., 2009	UPF3A and UPF3B
chr15	94899366	94899530	ENSG00000140563	MCTP2	ENST00000456504	constitutive	94899479	94899512	BR	Chan et al., 2009	UPF3A and UPF3B
chr15	94901711	94901841	ENSG00000140563	MCTP2	ENST00000456504	altAcceptor	94901801	94901833	BR	Chan et al., 2009	UPF3A and UPF3B
chr15	94901776	94901841	ENSG00000140563	MCTP2	ENST00000557742	altAcceptor	94901801	94901833	BR	Chan et al., 2009	UPF3A and UPF3B
chr15	94986148	94986189	ENSG00000140563	MCTP2	ENST00000456504	variant	94986150	94986182	BR	Chan et al., 2009	UPF3A and UPF3B
chr15	94945129	94945248	ENSG00000140563	MCTP2	ENST00000456504	constitutive	94945200	94945243	BU	Chan et al., 2009	UPF3A and UPF3B
chr15	94884042	94884153	ENSG00000140563	MCTP2	ENST00000456504	constitutive	94884092	94884154	BUR	Chan et al., 2009	UPF3A and UPF3B
chr15	94913316	94913409	ENSG00000140563	MCTP2	ENST00000456504	constitutive	94913362	94913408	BUR	Chan et al., 2009	UPF3A and UPF3B
chr15	94841430	94841959	ENSG00000140563	MCTP2	ENST00000456504	constitutive	94841799	94841874	EUR	Chan et al., 2009	UPF3A and UPF3B
chr3	131217023	131217121	ENSG00000114686	MRPL3	ENST00000264995	variant	131217033	131217067	BER	Wang et al., 2014	eIF4A3 and BTZ
chr3	131219274	131219365	ENSG00000114686	MRPL3	ENST00000264995	constitutive	131219284	131219306	BER	Wang et al., 2014	eIF4A3 and BTZ
chr3	131206524	131206584	ENSG00000114686	MRPL3	ENST00000264995	constitutive	131206541	131206575	BEUR	Wang et al., 2014	eIF4A3 and BTZ
chr2	183066415	183066516	ENSG00000115252	PDE1A	ENST00000435564	constitutive	183066421	183066465	BE	Chan et al., 2007	UPF3B
chr2	183129028	183129141	ENSG00000115252	PDE1A	ENST00000435564	constitutive	183129044	183129086	BR	Chan et al., 2007	UPF3B
chr2	183070667	183070792	ENSG00000115252	PDE1A	ENST00000435564	constitutive	183070669	183070714	BU	Chan et al., 2007	UPF3B
chr2	183088591	183088691	ENSG00000115252	PDE1A	ENST00000435564	constitutive	183088603	183088624	BU	Chan et al., 2007	UPF3B
chr2	183094733	183094873	ENSG00000115252	PDE1A	ENST00000435564	constitutive	183094748	183094769	BU	Chan et al., 2007	UPF3B
chr4	57876527	57876670	ENSG00000047315	POLR2B	ENST00000381227	constitutive	57876632	57876664	BEUR	Chan et al., 2007	UPF3B
chr4	57883209	57883407	ENSG00000047315	POLR2B	ENST00000381227	constitutive	57883362	57883407	BEUR	Chan et al., 2007	UPF3B
chr4	57861417	57861575	ENSG00000047315	POLR2B	ENST00000381227	constitutive	57861533	57861573	BR	Chan et al., 2007	UPF3B
chr4	57883726	57883894	ENSG00000047315	POLR2B	ENST00000381227	constitutive	57883851	57883892	BU	Chan et al., 2007	UPF3B
chr4	57888318	57888412	ENSG00000047315	POLR2B	ENST00000381227	constitutive	57888373	57888409	BU	Chan et al., 2007	UPF3B
chr4	57891052	57891165	ENSG00000047315	POLR2B	ENST00000381227	constitutive	57891124	57891165	BU	Chan et al., 2007	UPF3B
chr4	57891543	57891703	ENSG00000047315	POLR2B	ENST00000381227	constitutive	57891652	57891703	BU	Chan et al., 2007	UPF3B
chr4	57896370	57896565	ENSG00000047315	POLR2B	ENST00000381227	constitutive	57896510	57896565	BU	Chan et al., 2007	UPF3B
chr4	57872982	57873168	ENSG00000047315	POLR2B	ENST00000381227	constitutive	57873118	57873191	BUR	Chan et al., 2007	UPF3B
chr1	186645030	186645845	ENSG00000073756	PTGS2	ENST00000490885	altDonor	186645602	186645655	BUR	Gehring et al., 2005	RNPS1
chr1	186645599	186645845	ENSG00000073756	PTGS2	ENST00000367468	altDonor	186645602	186645655	BUR	Gehring et al., 2005	RNPS1
chr5	98109657	98109910	ENSG00000174136	RGMB	ENST00000308234	altAcceptor	98109773	98109830	BR	Chan et al., 2007	UPF3B
chr5	98109660	98109910	ENSG00000174136	RGMB	ENST00000504776	altAcceptor	98109773	98109830	BR	Chan et al., 2007	UPF3B
chr5	98109660	98109910	ENSG00000174136	RGMB	ENST00000504776	altAcceptor	98109861	98109903	BR	Chan et al., 2007	UPF3B
chr17	38787844	38788619	ENSG00000073584	SMARCE1	ENST00000469334	altDonor	38788456	38788495	BER	Chan et al., 2007	UPF3B
chr17	38792647	38792778	ENSG00000073584	SMARCE1	ENST00000348513	constitutive	38792646	38792685	BEUR	Chan et al., 2007	UPF3B
chr17	38798707	38798811	ENSG00000073584	SMARCE1	ENST00000348513	variant	38798742	38798774	BR	Chan et al., 2007	UPF3B
chr17	38793744	38793824	ENSG00000073584	SMARCE1	ENST00000348513	constitutive	38793791	38793819	BUR	Chan et al., 2007	UPF3B
chr1	70701184	70701233	ENSG00000116754	SRSF11	ENST00000370950	constitutive	70701191	70701222	BE	Chan et al., 2007	UPF3B
chr1	70710367	70710498	ENSG00000116754	SRSF11	ENST00000370950	constitutive	70710377	70710382	ER	Chan et al., 2007	UPF3B
chr17	74732236	74732546	ENSG00000161547	SRSF2	ENST00000359995	altBoth	74732532	74732556	BER	Gehring et al., 2005	BTZ
chr17	74731854	74731957	ENSG00000161547	SRSF2	ENST00000582449	variant	74731855	74731899	BEU	Gehring et al., 2005	BTZ

Table S6: Candidate exons. Related to Figure 4.

chromosome	peak_start	peak_end	peak_constitution	peak_strand	motif	RNASeq_max	validated
chr1	31408390	31408421	BU	-	AGGTAAG	1.92454392	yes
chr1	70759193	70759249	BUR	-	AGGTAAG	1.567413308	yes
chr1	109489973	109490002	BU	-	AGGTGAG	1.738607591	yes
chr1	117533144	117533161	BU	+	AGGTGAG	0.017108138	yes
chr1	220943849	220943889	BUR	+	AGGTAAG	0.627329422	yes
chr1	232709928	232709963	BR	-	AGGTAAG	0.167478378	yes
chr2	28633019	28633134	BUR	+	AGGTGAG	2.437001638	yes
chr2	101924302	101924333	BU	-	AGGTGAG	1.644242086	yes
chr3	127295012	127295084	BEUR	-	AGGTGAG	2.673589249	yes
chr5	60820926	60820966	BU	+	AGGTAAG	0.740998643	yes
chr6	2773253	2773295	BU	+	AGGTGAG	0.748281238	yes
chr6	32084101	32084254	BEUR	-	AGGTGAG	0.76875387	yes
chr6	170876536	170876595	BEUR	+	AGGTAAG	1.038479446	yes
chr8	144885747	144885755	BU	-	AGGTGAG	0.37534245	no
chr9	100780727	100780771	BR	+	AGGTGAG	0.139927314	yes
chr9	139294585	139294641	BUR	-	AGGTGAG	0.077491378	yes
chr10	126650932	126650974	BU	+	AGGTAAG	0.98799819	yes
chr11	123351535	123351602	BUR	+	AGGTAAG	0.731953671	yes
chr12	133220306	133220421	BR	-	AGGTGAG	2.340115173	no
chr16	590272	590410	BR	+	AGGTAAG	0.300513055	yes
chr16	56939303	56939353	BE	+	AGGTAAG	1.420646329	yes
chr17	12032843	12032886	BU	+	AGGTGAG	0.600925841	yes
chr19	3784892	3784921	BR	-	AGGTGAG	1.11493953	no
chr19	10505588	10505696	BR	-	AGGTAAG	9.804446049	no
chr19	19229840	19229883	BER	-	AGGTGAG	0.165178692	yes
chr20	62514181	62514239	BR	+	AGGTGAG	4.811648049	yes
chr21	46627254	46627299	BUR	+	AGGTGAG	1.263387152	yes
chr22	44727378	44727444	BR	-	AGGTGAG	0.009703228	yes
chr22	50875345	50875414	BEUR	+	AGGTGAG	1.33428187	yes
chr22	50879503	50879632	BEUR	+	AGGTGAG	0.501333486	yes
chrX	18910367	18910405	BR	+	AGGTGAG	2.105646862	yes

Table S7: siRNAs, primers and antibodies used in this study. Related to Figure 1.

	siRNA sequence (5' to 3')	ID
**eIF4A3**	GGAAGTATTTAGATCCAGA	SI05098044
	CCCATAAACTCTATACTTCTA	SI00107842
**BTZ**	CTGCTGAGGGCCAATACCCTA	SI05014849
	CTCCCTATCCATGGCACTAAA	SI05014856
**RNPS1**	GCCGTTCATGGTGGTCTTTCA	SI05027743
	AAACCCAAGATCGGAATGCAA	SI05027750
**UPF3B**	AAGTGAGGACTTCATCACATA	SI05096406
	CGGCAAGGACATGCATTGCAA	SI05096413
**UPF3A**	TTGGGTTTCATTCGTTTGTAA	SI03246411

	Primer sequence (5´ to 3´)	used in final iCLIP experiment replicate (rep) number
**L3 linker**	/5rApp/AGATCGGAAGAGCGGTTCAG/3ddC	
**RT primer 1**	/5Phos/NNAACCNNNAGATCGGAAGAGCGTCGTGGATCCTGAACCGC	UPF3B rep 2, RNPS1 rep 1
**RT primer 2**	/5Phos/NNACAANNNAGATCGGAAGAGCGTCGTGGATCCTGAACCGC	not used
**RT primer 3**	/5Phos/NNATTGNNNAGATCGGAAGAGCGTCGTGGATCCTGAACCGC	BTZ rep 1, GFP rep 1, eIF4A3 rep 2, UPF3B rep 2, PTB rep 2
**RT primer 4**	/5Phos/NNAGGTNNNAGATCGGAAGAGCGTCGTGGATCCTGAACCGC	not used
**RT primer 5**	/5Phos/NNCGCCNNNAGATCGGAAGAGCGTCGTGGATCCTGAACCGC	PTB rep 1, GFP rep 2
**RT primer 6**	/5Phos/NNCCGGNNNAGATCGGAAGAGCGTCGTGGATCCTGAACCGC	not used
**RT primer 7**	/5Phos/NNCTAANNNAGATCGGAAGAGCGTCGTGGATCCTGAACCGC	not used
**RT primer 8**	/5Phos/NNCATTNNNAGATCGGAAGAGCGTCGTGGATCCTGAACCGC	not used
**RT primer 9**	/5Phos/NNGCCANNNAGATCGGAAGAGCGTCGTGGATCCTGAACCGC	not used
**RT primer 10**	/5Phos/NNGACCNNNAGATCGGAAGAGCGTCGTGGATCCTGAACCGC	eIF4A3 rep 2, PTB rep 2, GFP rep 3
**RT primer 11**	/5Phos/NNGGTTNNNAGATCGGAAGAGCGTCGTGGATCCTGAACCGC	not used
**RT primer 12**	/5Phos/NNGTGGNNNAGATCGGAAGAGCGTCGTGGATCCTGAACCGC	not used
**RT primer 13**	/5Phos/NNTCCGNNNAGATCGGAAGAGCGTCGTGGATCCTGAACCGC	not used
**RT primer 14**	/5Phos/NNTGCCNNNAGATCGGAAGAGCGTCGTGGATCCTGAACCGC	not used
**RT primer 15**	/5Phos/NNTATTNNNAGATCGGAAGAGCGTCGTGGATCCTGAACCGC	eIF4A3 rep 1, RNPS1 rep 1, BTZ rep 2
**RT primer 16**	/5Phos/NNTTAANNNAGATCGGAAGAGCGTCGTGGATCCTGAACCGC	UPF3B rep 1, RNPS1 rep 2, BTZ rep 3
**Cut Oligo**	GTTCAGGATCCACGACGCTCTTCAAAA	
**Solexa 5´**	AATGATACGGCGACCACCGAGATCTACACTCTTTCCCTACACGACGCTCTTCCGATCT	
**Solexa 3´**	CAAGCAGAAGACGGCATACGAGATCGGTCTCGGCATTCCTGCTGAACCGCTCTTCCGATCT	

	Forward primer (5' to 3')	Location	Reverse primer (5' to 3')	Location
**eIF4A3 endo**	GATGCCGATGAACGTTGCTG	Exon 11–12	TTCAAAGGAAGGGAGACGGC	3´UTR
**eIF4A3**	TCATCGCACAGTCTCAGTCC	Exon 2–3	TTCCTGATGTCCTCGCCAAC	Exon 5
**BTZ endo**	AGGAGTCGGAGTGTGAGAGT	Exon 1–2	AGTGCTTTTGGTGTCAGGCT	Exon 4
**BTZ**	AGCACATGGATGGAGGACAA	Exon 7–8	CAGGCACACAGCATCCAAAC	3´UTR
**RNPS1 endo**	CGCTGAAGCACATGGATGGA	Exon 7	GGAGCGGGACCTTCTCCT	Exon 8–9
**RNPS1**	ACCAAAGAGACCTGAAGATGAGA	Exon 9–10	ACAGTCAGGACACCTAAGGC	3´UTR
**UPF3B endo**	CAATAAAGGTCAGGAATATCCCGC	Exon 3–4	AGGAAGCTCAAAAGTGGGGT	Exon 6
**UPF3B**	GCGACTGGCAAACAAGGACC	Exon 9–10	CTCCTTCCCAGTCACACTCG	3´UTR
**UPF3A endo**	GCCCCAGGAGACGTCACACA	Exon 7–8	CACTCCTTCTGGAAAGCCGT	Exon 8
**SC35C**	GGCGTGTATTGGAGCAGATGTA	3´UTR	CTGCTACACAACTGCGCCTTTT	3´UTR
**SC35D**	CGGTGTCCTCTTAAGAAAATGATGTA	3´UTR	CTGCTACACAACTGCGCCTTTT	3´UTR
**SC35WT**	CGTGCCTGAAACTGAAACCA	3´UTR	TTGCCAACTGAGGCAAAGC	3´UTR
**TGM2**	GGGGTGAGAGAGGAAAGACC	Exon 13	AGTGCTTTTGGTGTCAGGCT	Exon 13
**IDUA**	GGCCATGGTGGTGAAGGTCAT	Exon 7–8	GAAGGCATTGTCGTTGCTCAG	Exon 8
**ATHL1**	TGGGACCAGGACCTCTGGATG	Exon 4–5	AGTCTGCACTCTCCCAGGCAA	Exon 6
**ZNF217**	ACTGTTACCGCAGGACTGTGT	Exon 3	TTTCCTTCTAACGTCGAGCTG	Exon 3–4
**ZCCHC2**	TTCTACTGAAGCTTCCAAAGG	Exon 4–5	GGAGTCTGATGATATGGACTG	Exon 6
**GAPDH**	TGAGCTTGACAAAGTGGTCG	Exon 8	GGCTCTCCAGAACATCATCC	Exon 8
**ZXDB**	TGTGTGACCCTGAGCAAGTC	3´UTR	GCCACATCACTGAAAAACGTCC	3´UTR
**HIST1H4B**	CTAGGCGTGGTGGGGTTAAG	Exon 1	CGTACAGAGTGCGTCCTTGA	Exon 1

	Concentration	Secondary antibody	Company	ID
**α-eIF4A3**	1:1,000	Rabbit	Genscript	custom-made*
**α-BTZ**	1:1,000	Rabbit	Abcam	ab90651
**α-RNPS1**	1:1,000	Rabbit	Gift from Dr. Akila Mayeda	
**α-UPF3B**	1:1,500	Rabbit	Sigma-Aldrich	HPA001800
**α-Y14**	1:1,000	Mouse	ImmuQuest	IQ220
**α-hnRNPA 1**	1:1,000	Mouse	Santa Cruz	sc-32301
**α-Actin**	1:1,000	Mouse	Sigma-Aldrich	A1978
**α-Tubulin**	1:1,000	Mouse	Life Technologies	480011
**α-GAPDH**	1:1,000	Goat	GeneTex	GTX89409
**α-GFP**	1:1,000	Rabbit	Abcam	ab290
**α-Mouse**	1:10,000	–	Sigma-Aldrich	A9044
**α-Rabbit**	1:10,000	–	Sigma-Aldrich	A0545
**α-Goat**	1:10,000	–	Sigma-Aldrich	A9452

*DDX48 custom made against ATSGSARKRLLKEEDC peptide

Supplemental Experimental Procedures

Stable HeLa cell lines

A HeLa host cell line containing a Flp recombination target (FRT) site combined with an antibiotic (Zeocin™) resistance gene and stably expressing a tetracycline repressor was used to generate cell lines expressing the fusion proteins. The gene of interest was cloned into a pcDNA5 vector containing a FRT and a tetracycline-on (TO) system, pcDNA5/FRT/TO, and integrated into the host cell line by the pOG44 Flp recombinase expressing plasmid. The FRT site located upstream of another antibiotic (hygromycin) resistance gene in the pcDNA5/FRT/TO vector was used for the integration of the fusion protein into the HeLa host cell line. In addition, the hygromycin resistance gene enabled the specific selection of colonies containing the fusion gene. The human *cytomegalovirus* (CMV) promoter together with the TO system allowed the expression of fusion proteins close to the level of the endogenous counterpart.

For each stable cell line, 1,000,000 cells were seeded in a 10 cm dish. On day 2, the medium was renewed 2 h before the transfection of the plasmids using the calcium phosphate method. For the transfection, pOG44 Flp recombinase plasmid was mixed with the pcDNA5/FRT/TO/TEV/Protein-GFP plasmids in a ratio of 1:9 and H2O was added to a final volume of 450 µl. The plasmids were then mixed with 50 µl of 2.5 M $CaCl_2$ and 500 µl of 2 x borate buffered saline (BBS). After incubation for 20 min at room temperature, the mixture was added to the cells in 10 ml Dulbecco's Modified Eagle Medium (DMEM) and incubated for 20 h at 37 °C and 5% CO2. On day 3, the cells were split into three 10 cm dishes using different ratios of cells (5:10, 3:10 and 2:10). On day 4, DMEM was exchanged by fresh DMEM containing the selection antibiotics blasticidin (final concentration 5 µg/ml) and hygromycin (final concentration 200 µg/ml). The cells were monitored for approximately two weeks. During this time, the medium was exchanged when appropriate. Single colonies were picked with 10 µl trypsin and transferred to a 12-well plate containing 1 ml DMEM with selection antibiotics (blasticidin and hygromycin) per well. On week 4, colonies were detached with 70 µl trypsin and transferred into two wells of a 6-well plate, each containing 2 ml DMEM with selection antibiotics. The expression of a GFP-fusion construct was controlled by fluorescence microscopy in one well by the addition of 1 µg/ml doxycycline, which belongs to the tetracycline antibiotics class and thus can be used in Tet-on systems to replace tetracycline. In case the fusion protein was expressed, the non-induced well was passaged to a T175 flask and, after the cells reached 80% confluence, frozen in two aliquots.

The stable HeLa cells were frozen in liquid nitrogen in batches to ensure that the experiments will be performed on freshly thawed cells from the same passage. The first experiments were conducted from passage 2 onwards and the cells were discarded at passage 10.

siRNA knockdown and complementation assay

For the siRNA rescue experiments, 100,000 cells in 2 ml DMEM GlutaMax-I medium (supplemented with 10% (v/v) FCS and 1% (v/v) penicillin/streptomycin) were seeded per well of a 6-well plate. The cells were transfected with siRNAs 6–8 h after seeding. For each sample, 1 µl siRNA (siRNA stocks 10 pmol/µl) was diluted in 100 µl pre-warmed reduced serum medium (Opti-MEM®) without antibiotics and 12 µl transfection reagent (HiPerfect) were added. The mixture was vortexed and incubated for 10 min at room temperature. In the meantime, the cells were washed twice with Opti-MEM®. The siRNA mixture was added drop-wise to 887 µl Opti-MEM per 6-well. Thus, the cells were incubated overnight with a siRNA concentration of 10 nM. On the following day, Opti-MEM® was replaced by 2 ml DMEM containing appropriate doxycycline concentration for the induction of the fusion proteins (for RNPS1 0.1 ng/ml and for the other EJC proteins and controls 1 ng/ml doxycycline). The cell samples were split for RNA and protein analysis. The RNA was isolated by 750 µl of TRIzol® and the cDNA synthesis was performed with oligo-(dT) primer. The protein sample was centrifuged at 1100 g for 10 min at 4 °C. After removal of the supernatant, the cells were lysed in 50 µl RIPA buffer (150 mM NaCl, 0.5% (w/v) sodium deoxycholate, 50 mM Tris-Base, 1% (w/v) nonyl phenoxypolyethoxylethanol (NP-40), 0.1% (w/v) SDS and 1 x protease inhibitor) and incubated for 10 min on ice. The protein samples were stored at −80 °C prior to immunoblot analysis. The siRNA and primer sequences are listed in Table S7.

Immunoblots

For immunoblots, proteins were separated using SDS-PAGE. The protein samples were supplemented with 6 x loading buffer and heated for 5 min at 95 °C. Protein size was determined using 10 µl of the protein ladder (PageRuler™ Plus). After electrophoresis at 20–60 mA, protein transfer was carried out at 1mA/cm2 of the membrane (Westran-S) for 1 h. To slow down this protein transfer for small proteins, like Y14 (20 kDa), the anode and cathode buffers were supplemented with 20% methanol. The membrane was incubated for 1 h in 5% skim milk in Tris-buffered saline plus Tween (TBS-T) to block unspecific binding sites prior to the antibody labeling. The primary antibody was incubated under gentle shaking overnight at 4 °C (see Table S7 for antibodies). Unbound antibody was removed by three washing steps for 10 min with TBS-T at room temperature. The secondary antibody was incubated for 1 h at room temperature. Subsequently, three washing

steps, each of 10 min at room temperature with TBS-T, were carried out to remove unbound secondary antibody prior to the protein detection. The proteins were labeled with a chemiluminescence kit and detected with a multi-imaging system (Fusion Fx).

iCLIP

Day 1 and 2 – Preparation of stable cells

For each iCLIP replicate, 3,000,000 stable HeLa cells were seeded in two 15 cm dishes, each containing 30 ml DMEM supplemented with 10% (v/v) FCS and 1% (v/v) penicillin/streptomycin. The cells were incubated at 37 °C and 5% CO2 for two days. The GFP-fusion constructs were induced directly after seeding with a final doxycycline concentration of 1 ng/ml (0.1 ng/ml for RNPS1-GFP) to adjust the expression level of the recombinant protein close to that of the endogenous counterpart.

Day 3 – UV cross-linking

To stop protein translation, the cells were treated with a final concentration of 100 µg/ml cycloheximide. A cross-link between the RBP and the bound RNA was mediated by an irradiation with 150 mJ/cm^2 and a wavelength of 254 nm.

Day 4 – Immunoprecipitation

After harvesting the cells, the cells were lysed (all buffers as in (Konig et al., 2011)) and protein concentration was adjusted to approximately 2–3 mg/ml and a final volume of 1400 µl. To increase the salt concentration, 350 µl of 5 x high salt buffer were added to the sample. Meanwhile, 30 µl of agarose beads coupled to a GFP antibody (GFP-Trap®_A) were washed three times with 1 ml lysis buffer and spinning at 1000 g at 4 °C for 1 min. The beads were resuspended in 60 µl lysis buffer and transferred to the sample. To resuspend the beads thoroughly, the pipette tip had to be cut using a sharp blade. The sample was incubated at 4 °C and 10 rpm in a rotator mixer (RM Multi-1) for 2 h.

The proteins bound to antibody-coupled beads were pelleted at 1000 g at 4 °C for 30 s and the supernatant was stored as flow-through. All subsequent washing steps were performed with 1 ml washing buffer and a 2 min incubation at 10 rpm in a rotator mixer followed by centrifugation at 1000 g for 1 min. To remove unbound proteins, the beads were washed twice with high salt buffer at room temperature to avoid precipitation of SDS and protease inhibitors. The samples were further purified by two washing steps with medium salt buffer and one washing step with low salt buffer containing only 0.5 x of RNase inhibitors (RNasin). For the partial RNA digestion, the sample was resuspended in 1 ml low salt buffer without RNasin and 10 µl of 1:50 dilution of RNase I (final concentration 1:5000, 0.02 U/µl) and 2 µl of Turbo DNase. The RNA was partially digested by incubation for exactly 3 min at 37 °C at 1100 rpm in a heat block (Thermomixer compact). Subsequently, the beads were washed with 1 ml of high salt buffer containing protease and RNase inhibitors at room temperature, followed by two washing steps with low salt buffer.

To dephosphorylate the 3´end of the RNA fragments, the beads were resuspended in 20 µl PNK mix (15 µl H2O, 4 µl 5 x PNK buffer pH6.5, 0.5 µl PNK and 0.5 µl RNasin) and incubated at 37 °C for 20 min at 1100 rpm in a heat block. The enzyme was inactivated by two high salt and two low salt buffer washing steps. For ligation of a L3 linker to the RNA fragments – being essential for the reverse transcription – the supernatant was removed and the sample incubated in 20 µl ligation mix (9 µl H2O, 4 µl 4 x ligation buffer, 1 µl RNA ligase, 0.5 µl RNasin, 1.5 µl 20 µM pre-adenylated linker L3 and 4 µl polyethylene glycol (PEG) 400) at 16 °C using 1100 rpm overnight in a heat block.

Day 5 – RNA isolation

Unligated L3 linker was removed by three washing steps with high salt buffer (containing protease and RNase inhibitors). After the second washing step, the samples were transferred into new low DNA binding tubes to remove potentially non-ligated L3 linker bound to the plastic surface. Subsequently, the samples were washed twice with low salt buffer containing RNase inhibitors (but no protease inhibitors) to prepare the samples for protein digestion. In order to digest any RNases in the proteinase K (PK) buffer, the buffer was treated with proteinase K (10 µl) at 37 °C for 20 min. Afterwards, 200 µl of this mixture were transferred to each sample and the tubes were incubated at 37 °C. After 20 min, 200 µl PK-Urea buffer were added to each sample followed by an incubation for another 20 min at 37 °C to denature the proteins.

The RNA was isolated using phase lock gel (PLG™) heavy tubes. To facilitate the RNA precipitation, the samples were mixed with 0.5 µl glycogen (GlycoBlue™) and 40 µl 3M sodium acetate pH 5.2. The precipitation was performed with 1 ml 100% ethanol at −20 °C overnight.

Day 6 – cDNA synthesis

The precipitated RNA was pelleted at 16000 g at 4 °C for 20 min. After removal of the supernatant, the pellet was washed with 80% (v/v) ethanol and centrifuged for another 2 min. Ethanol was completely removed and the pellets were air-dried for 2 min at room temperature. A volume of 6.25 µl was further transferred to a new 200 µl tube to continue the iCLIP experiment. The remaining 1.75 µl were used to measure the size and concentration of the isolated RNA with a Bioanalyzer2100 instrument and RNA Pico chips. The size distribution of the RNA should have a peak within 25–300 nts.

For iCLIP, 0.5 µl RT primer (0.5 pmol/µl) and 0.5 µl dNTP (10 mM each) were mixed with the sample and incubated for 5 min at 70 °C in a PCR machine. For each replicate and experiment performed in one batch, a different RT primer was used. Table S7 lists all possible RT primer sequences including the barcodes. The RT primers contained a sample and a random barcode. The sample barcode allows multiplexing of samples and the random barcode enables the detection and elimination of PCR duplicates. The RT primers were checked for their efficiency in reverse transcription reactions by subcloning, using TA cloning and sequencing before final use in iCLIP experiments. In brief, for each iCLIP library with different RT primers at least 5 colonies were picked and the plasmids isolated. The plasmid sequences were evaluated regarding meaningful inserts, i.e. fragments upstream of exon-exon junctions for the EJC iCLIP experiments and, even more importantly, if the introduced sample barcode was present. Only RT primers that fulfilled these criteria were used for the subsequent iCLIP experiments. More specifically, the barcodes used were swapped to other RBPs and to the GFP control in further biological replicates in order to rule out any bias that was introduced by a single RT primer.

For iCLIP experiments, the RT primers were annealed to the L3 linker by slowly cooling down (−0.1 °C/s) the sample from 70 °C to 25 °C. For the reverse transcription, 2.75 µl reverse transcriptase mix (2 µl 5 x reverse transcriptase buffer, 0.5 µl 0.1M DTT and 0.25 µl Superscript III) were added. The second cDNA synthesis step was performed at 50 °C instead of the initial 42 °C in order to improve the denaturing of RNA secondary structures. The mixture was diluted with 90 µl Tris-EDTA (TE) buffer and transferred to a new low DNA binding tube containing 1 µl glycogen and 10 µl 3M sodium acetate pH 5.2. After vortexing, the cDNA was precipitated with 250 µl 100% ethanol at −20 °C overnight.

Day 7 – cDNA size selection

The precipitated cDNA was pelleted, washed as described at day 6 and resuspended in 6 µl H2O. For the gel purification of the cDNA, the same volume of 2 x Tris-borate EDTA urea (TBE-urea) loading buffer was added. For one gel, 3 µl of low molecular weight DNA ladder were mixed with the same volume of 2 x TBE-urea loading buffer. The samples and the marker were heated for 3 min at 80 °C. The samples plus the marker were loaded on the gel and exposed to a voltage of 180V for approximately 40 min. Afterwards, slices of low, medium and high molecular weight cDNAs were cut from the gel according to a size range of 70–85, 85–120 and 120–200 nts. Each gel slice was transferred to a 1.5 ml low DNA binding tube and 400 µl TE buffer were added. The gel slice was crushed into small pieces using a pestle and incubated for 2 h at 37 °C under agitation at 1100 rpm. Meanwhile, two glass microfiber filters were placed into a centrifuge tube filter column using clean tweezers. Each sample was transferred to one of these columns and centrifuged for 1 min at 16,000 g at room temperature. The flow-through was transferred to a low DNA binding tube containing 0.5 µl glycogen and 40 µl 3M sodium acetate pH 5.2. The cDNA was precipitated with 1 ml 100% ethanol at −20 °C overnight.

Day 8 – cDNA circularization

The size-selected and precipitated cDNA was pelleted, washed as described at day 6, resuspended in 8 µl ligation mix (6.5 µl H2O, 0.8 µl 10 x CircLigase buffer II, 0.4 µl 50 mM MnCl2 and 0.3 µl CircLigase II) and transferred to a PCR tube. For circularization of the cDNA, the samples were incubated for 1 h at 60 °C in a pre-heated PCR machine to avoid evaporation of the sample into the tube lid. Afterwards, 30 µl of oligonucleotide annealing mix (26 µl H2O, 3 µl SmartCut buffer and 1 µl 10 µM Cut Oligo, see Table S7) were added. The sample was incubated at 95 °C for 1 min and then the temperature was decreased by 1 °C/10 s to a final temperature of 25 °C. The annealed oligonucleotide was cut by 2 µl of the restriction enzyme BamHI-HF for 30 min at 37 °C. The sample was mixed with 50 µl TE buffer and transferred to a low DNA binding tube containing 1 µl glycogen and 10 µl 3M sodium acetate pH 5.2. The cDNA was precipitated with 250 µl 100% ethanol at −20 °C overnight.

Day 9 – cDNA library preparation

The precipitated cDNA was pelleted, washed as described at day 6, resuspended in 19 µl H2O and transferred to a PCR tube. For the PCR amplification (25 cycles), 1 µl of 10 µM 5′ and 3′Solexa primer and 20 µl DNA polymerase (Accuprime Supermix 1) were added to the cDNA.

Afterwards, primer dimers and fragments smaller than 130 nts were removed from the cDNA library by magnetic beads (AMPure® XP) coated on their surface with carboxyl molecules, which can bind DNA molecules. The beads were vortexed and equilibrated for 30 min at room temperature. To each library, beads in a

volume corresponding to the 1.8 volume of the cDNA sample were added. In practice, 35 µl of cDNA library were diluted to a total volume of 50 µl with H2O and 90 µl of beads were added. After vortexing, the sample was incubated for 15 min at room temperature. Then, the tube was put to a magnetic particle concentrator (DynaMagTM-2) for 5 min to capture the cDNAs that were longer than 130 nts. The tube was maintained at the magnet until the elution step. The supernatant was discarded and 200 µl of freshly prepared 80% (v/v) ethanol was added for 30 s to wash the beads without disturbing the bead pellet. This step was repeated and then the ethanol was completely removed by incubating the tubes with open lid for 5 min at room temperature. To elute the captured cDNAs, the pellet was resuspended in 15 µl H2O and incubated for 2 min at room temperature. The tube was placed back to the magnetic stand for 5 min to remove the beads. The supernatant, containing the purified cDNA iCLIP library, was transferred to a low DNA binding tube and the purity was analyzed with a high sensitivity DNA chip. Therefore, 1 µl of the samples was diluted 1:10 with H2O and the analysis was performed according to the user manual [109]. The iCLIP library should only contain one peak corresponding to the selected cDNA size plus the added primer length. In general, the iCLIP library should be free from any primer dimers around 120 nts.
For the final sequencing, only the medium and high molecular weight cDNA libraries were submitted, since the low molecular weight cDNA libraries showed low complexity for the iCLIP reads in the test runs. Prior to multiplexing, the cDNA-concentrations of the libraries were measured with a Qubit™ dsDNA high sensitivity assay. Purified libraries with a fragment size in a range of 150–300 nts were pooled in equimolar ratios to a final cDNA concentration of 2 µg/µl and a volume of at least 30 µl. The cDNA libraries were sequenced with 50 bp single-end on an Illumina HiSeq2000.

RIP

For RNA extraction, the beads (GFP-Trap®_A) were washed four times with low salt buffer supplemented with 0.1% RNasin. To dissociate the complex from the bound RNA, 40 µl clear sample buffer (100 mM Tris-HCl pH 6.8, 4% SDS, 10 mM EDTA and 100 mM DTT) were added and the protein-bead samples were centrifuged for 5 min at 1000 g at room temperature. The input and the precipitated samples were diluted in 200 µl PBS and 750 µl TRIzol® to prepare them for the RNA isolation. The isolated RNA was reverse transcribed into cDNA and the samples were spiked with 2 µl TATAA universal RNA, which served as a quality control throughout the entire experimental workflow. For each RBP, three independent biological experiments were performed. To minimize the error in the q-RT-PCR every target was measured in triplicate and reported in C_t (threshold cycle) values for each independent experiment. The arithmetic mean of the technical replicates ($C_{t\ mean\text{-}triplicate}$) had to be adjusted by a dilution factor since the same RNA quantity for cDNA synthesis was used for each RBP. This dilution factor ($C_{t\ \mathrm{dilution\text{-}factor}}$) was calculated by the negative logarithm of the ratio between the RNA quantity that was used for the cDNA synthesis ($RNA_{cDNA\text{-}synthesis}$) and the RNA quantity retained after RIP ($RNA_{after\ RIP}$).

$$C_{t\ \mathrm{dilution-factor}} = -\log 2\ \mathrm{RNA}_{cDNA\text{-}synthesis} / \mathrm{RNA}_{afterRIP}$$

To calculate the relative RNA concentration of the target ($C_{t\ target\text{-}adjusted}$) this dilution factor has to be subtracted from the $C_{t\ mean\text{-}triplicate}$.

$$C_{t\ target-adjusted} = C_{t\ mean-triplicate} - C_{t\ dilution-factor}$$

This equation was applied to every RBP for both immunoprecipitation and input samples and the target enrichment ($C_{t\ enrichment}$) was calculated by subtracting the immunoprecipitation from the input values.

$$C_{t\ enrichment} = Input\ C_{t\ target-adjusted} - IP\ C_{t\ target-adjusted}$$

Afterwards, the target enrichment was adjusted (C*t enrichment-adjusted*) for the loss of RNA during the entire workflow by subtracting the spike-in Ct enrichment from the Ct enrichment target values.

$$C_{t\ enrichment-adjusted} = Target\ C_{t\ enrichment} - Spike-in\ C_{t\ enrichment}$$

Finally, for every target the percentage of the immunoprecipitated RNA compared to the input RNA (*Percentage of IP Input*) was calculated to determine which target RNA binds best to the RBPs after RIP.

$$Percentageof\ IP\ Input = 100 \times 2^{Ct\ enrichment-adjusted}$$

Peak analysis

Because the replicates had very similar distribution patterns across the different genomic regions, the peak analysis was repeated after merging the read-files of the three replicates. Consequently, the detection of peaks became more powerful with the increased number of reads. The detected peaks per RBP were filtered for high-confidence targets by applying a filter based on the GFP control. The number of peaks before and after the GFP filtering is listed in Table S3.

The number of false-positive hits was lowered by filtering for high GFP counts. The mean overamplification rate for the three biological replicates of GFP was divided by the mean overamplification rate of the other five proteins. This overamplification-factor was multiplied to the iCLIP read signal. We then applied a two step filtering approach. First, all peaks were removed that have a higher overamplification-factor adjusted CPM value in the GFP control than in the other libraries. Second, we scanned the individual replicates of an iCLIP experiment and kept only the peaks that have a higher summed CPM value compared to the GFP control. The overlap of the detected peaks was calculated with the software R (version 3.1.2) applying the Bioconductor package GenomicRanges (version 1.18.4) (Lawrence et al., 2013).

2 out of 4 approach

In order to analyze whether different proteins bind to the same genomic location, binding sites of different RBPs which overlapped with at least one nucleotide were merged into one contig. As a result, the number of detected binding sites was reduced from 521,171 to 346,396. The RNA binding to specific sites was controlled and confirmed by analysis of the PTB iCLIP dataset which possessed over 80% of unique binding sites that were not shared with any of the EJC components (see restricted peaks to one RBP in Table S3). By contrast, eIF4A3 and UPF3B shared 93% and 88% of the binding sites (showing only 7% and 12% unique binding sites), respectively. Similarly, BTZ and RNPS1 (with comparable or even larger libraries than PTB) accounted for less unique peaks (54% and 39%) compared to PTB. This indicated that all four EJC components share common sites where they bind directly to the RNA in close proximity and thus form EJCs with a homogeneous composition.

To further study the co-deposition of different EJC proteins as stringently as possible, all PTB binding sites were excluded from the analysis. This filtering resulted in approximately 200,000 sites at which at least one EJC protein, but not PTB, was deposited (Table S3).

This analysis was further specified to the EJC function by analyzing sites that were occupied by at least two out of 4 EJC proteins (Table S3 bottom part). These binding sites were termed high-confidence binding sites.

Supplemental References

Konig, J., Zarnack, K., Rot, G., Curk, T., Kayikci, M., Zupan, B., Turner, D.J., Luscombe, N.M., and Ule, J. (2011). iCLIP--transcriptome-wide mapping of protein-RNA interactions with individual nucleotide resolution. Journal of visualized experiments : JoVE.

Lawrence, M., Huber, W., Pages, H., Aboyoun, P., Carlson, M., Gentleman, R., Morgan, M.T., and Carey, V.J. (2013). Software for computing and annotating genomic ranges. PLoS computational biology *9*, e1003118.
