# Supplementary material for: Exon Junction Complexes Show a Distributional Bias toward Alternatively Spliced mRNAs and against mRNAs Coding for Ribosomal Proteins
Source: Cell Rep. 2016 Jul 28;16(6):1588–603. doi: 10.1016/j.celrep.2016.06.096 (PMC4978704; doi:10.1016/j.celrep.2016.06.096)
Supplement: Document S2. Article plus Supplemental Information [file mmc2.pdf]

Cell Reports

Article

Exon Junction Complexes Show a Distributional Bias toward Alternatively Spliced mRNAs and against mRNAs Coding for Ribosomal Proteins

Graphical Abstract

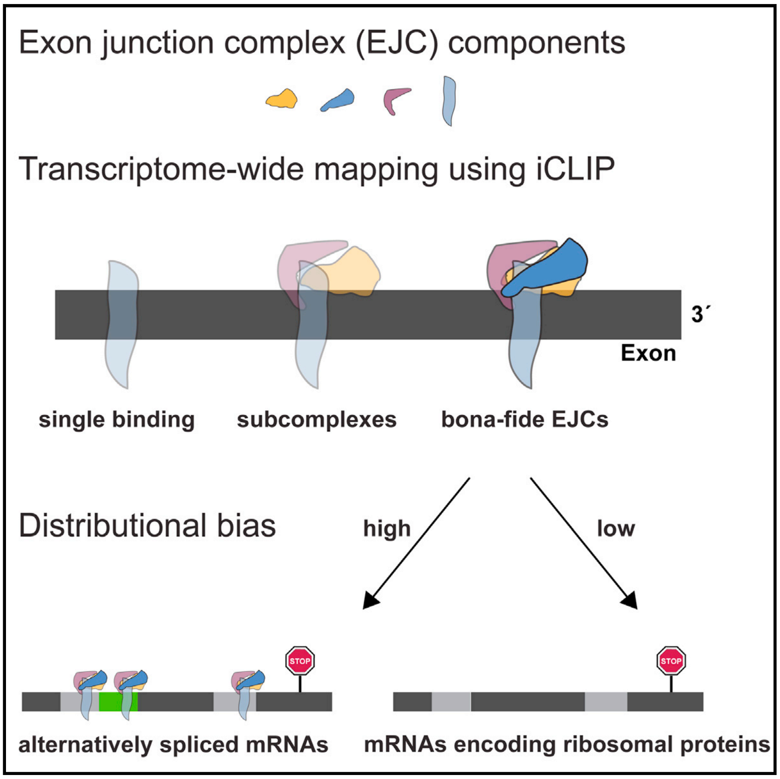

Highlights

- iCLIP analyses of EJC components provide a comprehensive map of bona fide EJCs
- EJC proteins, in particular BTZ, are largely restricted to canonical deposition sites
- EJCs are enriched on alternatively spliced mRNAs
- EJCs are underrepresented on mRNAs encoding ribosomal proteins

Authors

Christian Hauer, Jana Sieber, Thomas Schwarzl, ..., Anne-Marie Alleaume, Matthias W. Hentze, Andreas E. Kulozik

Correspondence

hentze@embl.de (M.W.H.), andreas.kulozik@med.uni-heidelberg.de (A.E.K.)

In Brief

Exon junction complexes govern multiple critical decisions in posttranscriptional gene regulation. Using all four RNA binding subunits of the complex, Hauer et al. provide a comprehensive map of bona fide EJCs across a mammalian transcriptome and show enrichment on alternatively spliced mRNAs and underrepresentation on RNAs encoding ribosomal proteins.

Accession Numbers

E-MTAB-4215

Hauer et al., 2016, Cell Reports *16*, 1588–1603
August 9, 2016 © 2016 The Author(s).
http://dx.doi.org/10.1016/j.celrep.2016.06.096

CellPress

OPEN ACCESS
CellPress

Cell Reports
Article

Exon Junction Complexes Show a Distributional Bias toward Alternatively Spliced mRNAs and against mRNAs Coding for Ribosomal Proteins

Christian Hauer,[1,2,3] Jana Sieber,[1,2] Thomas Schwarzl,[3] Ina Hollerer,[1,2,3] Tomaz Curk,[3,4] Anne-Marie Alleaume,[3] Matthias W. Hentze,[2,3,*] and Andreas E. Kulozik[1,2,*]
[1]Department of Pediatric Oncology, Hematology, and Immunology, University of Heidelberg, Im Neuenheimer Feld 430, 69120 Heidelberg, Germany
[2]Molecular Medicine Partnership Unit (MMPU), Im Neuenheimer Feld 350, 69120 Heidelberg, Germany
[3]European Molecular Biology Laboratory (EMBL) Heidelberg, Meyerhofstrasse 1, 69117 Heidelberg, Germany
[4]Faculty of Computer and Information Science, University of Ljubljana, Vecna Pot 113, 1000 Ljubljana, Slovenia
*Correspondence: hentze@embl.de (M.W.H.), andreas.kulozik@med.uni-heidelberg.de (A.E.K.)
http://dx.doi.org/10.1016/j.celrep.2016.06.096

SUMMARY

The exon junction complex (EJC) connects spliced mRNAs to posttranscriptional processes including RNA localization, transport, and regulated degradation. Here, we provide a comprehensive analysis of bona fide EJC binding sites across the transcriptome including all four RNA binding EJC components eIF4A3, BTZ, UPF3B, and RNPS1. Integration of these data sets permits definition of high-confidence EJC deposition sites as well as assessment of whether EJC heterogeneity drives alternative nonsense-mediated mRNA decay pathways. Notably, BTZ (MLN51 or CASC3) emerges as the EJC subunit that is almost exclusively bound to sites 20–24 nucleotides upstream of exon-exon junctions, hence defining EJC positions. By contrast, eIF4A3, UPF3B, and RNPS1 display additional RNA binding sites suggesting accompanying non-EJC functions. Finally, our data show that EJCs are largely distributed across spliced RNAs in an orthodox fashion, with two notable exceptions: an EJC deposition bias in favor of alternatively spliced transcripts and against the mRNAs that encode ribosomal proteins.

INTRODUCTION

Exon junction complexes (EJCs) are deposited in a splicing-dependent and essentially sequence-independent fashion approximately 20–24 nt upstream of exon-exon boundaries (Le Hir et al., 2000). The mammalian EJC is composed of four core subunits: eukaryotic translation initiation factor 4A3 (eIF4A3), barentsz (BTZ, CASC3, and MLN51), RNA binding protein 8A (RBM8A and Y14), and mago nashi homolog (MAGOH) (Ballut et al., 2005; Degot et al., 2004). In addition, peripheral EJC components bind to the EJC core. These proteins include the up-frameshift protein 3B (UPF3B), the RNA binding protein with serine-rich domain 1 (RNPS1), and the apoptotic chromatin condensation inducer in the nucleus (Acinus) to form a complex of approximately 335 kDa (Kim et al., 2001; Le Hir et al., 2000; Mayeda et al., 1999; Tange et al., 2005). Fully assembled EJCs are dissociated and recycled by the ribosome and the disassembly factor PYM (Gehring et al., 2009b). Because EJC components bind to (pre)-mRNA and remain associated with the mature mRNA until after export, the EJC and its components play important roles in the posttranscriptional fate of the mRNA including (pre)-mRNA splicing (Michelle et al., 2012), export (Le Hir et al., 2001), stability (Gehring et al., 2005; Palacios et al., 2004), translation (Chazal et al., 2013; Nott et al., 2004), and localization (Hachet and Ephrussi, 2004; Palacios et al., 2004).

Considering the broad role of EJCs in posttranscriptional processes, it is important that the abundance of the EJC components varies and is insufficient by orders of magnitude to bind and remain bound to all EJCs of a cell's transcriptome (Gehring et al., 2009b). Previous transcriptome-wide studies used the binding sites of eIF4A3 as a proxy for EJC deposition sites and found that 40%–50% of these sites are located outside the canonical deposition site (Saulière et al., 2012; Singh et al., 2012). By contrast, we provide a comprehensive map of bona fide EJCs across a mammalian transcriptome by using individual nucleotide resolution and immunoprecipitation (iCLIP) analyses for all four RNA binding subunits of the EJC: eIF4A3, BTZ, UPF3B, and RNPS1; the latter two bind to the core EJC and have been directly implicated in alternative nonsense-mediated decay (NMD) pathways (Chan et al., 2007, 2009; Gehring et al., 2005; Huang et al., 2011). In particular, we explored: (1) the distribution of these four EJC subunits across a cell's transcriptome; (2) the subunit composition of EJCs; and (3) quantitative differences for EJC components on functionally defined subsets of mRNAs.

RESULTS

Establishment of a Validated Experimental System for Identifying Regions of EJC Enrichment

We chose and validated HeLa cells as a system with active NMD (Figure S1A; Boelz et al., 2006) to derive cell lines that stably

 © 2016 The Author(s).
This is an open access article under the CC BY license (http://creativecommons.org/licenses/by/4.0/).

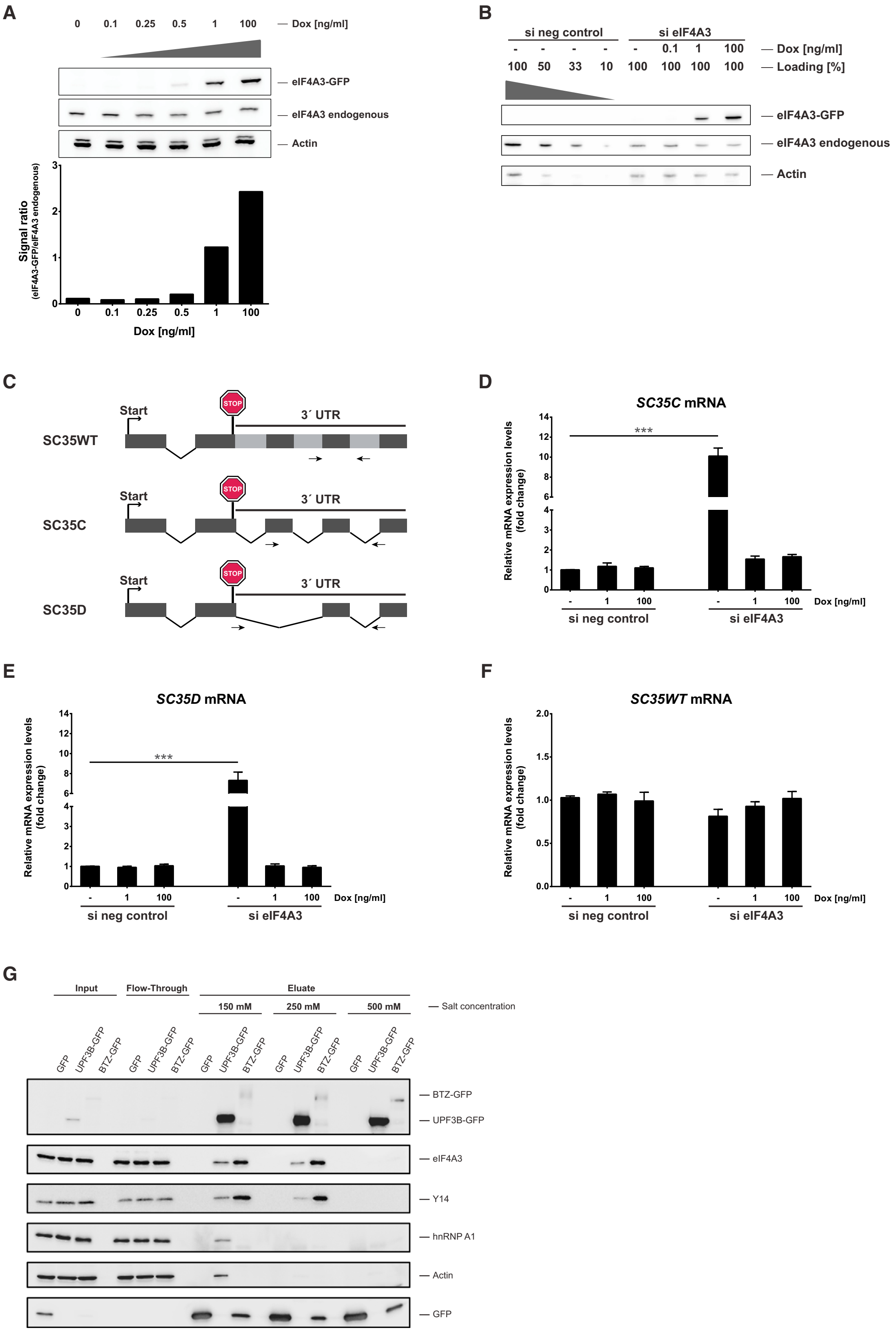

(legend on next page)

express inducible genes encoding GFP-tagged eIF4A3, BTZ, UPF3B, and RNPS1, respectively. The induction conditions for the tagged proteins were titrated to correspond to the expression of their endogenous counterparts (Figures 1A and S1B–S1D). The functionality of the GFP-tagged proteins was validated by small interfering (si)RNA complementation and coimmunoprecipitation (coIP) experiments (Figures 1B–1G). The depletion of endogenous eIF4A3 (approx. 30% residual protein) and the induction of eIF4A3-GFP by different doxycycline concentrations is shown in Figure 1B and the effect on endogenous NMD targets was measured by quantitative (q)RT-PCR. These experiments confirmed that the endogenous NMD targets *SC35C* and *SC35D* (Figure 1C; Sureau et al., 2001) were upregulated 8- to 10-fold upon depletion of endogenous eIF4A3 protein (Figures 1D and 1E), whereas the depletion of endogenous eIF4A3 had no effect on the *SC35WT* mRNA that is NMD-insensitive (Figure 1F). Since 1 ng/ml doxycycline induced the expression of eIF4A3-GFP close to that of endogenous eIF4A3 (Figure 1A) and rescued NMD completely (Figures 1D and 1E), we conclude that the eIF4A3-GFP fusion protein is fully active and thus suitable for iCLIP. Successful RNAi and rescue experiments were also performed for HeLa cells expressing either BTZ-GFP or RNPS1-GFP, although the upregulation of the respective NMD targets was less pronounced than for eIF4A3 (Figures S1B and S1C). In case of UPF3B, it has been challenging to test the functionality following RNAi and rescue, which may be related to the function of the highly homologous UPF3A that has been reported both to compensate and to antagonize UPF3B functions (Chan et al., 2009; Shum et al., 2016). We therefore validated UPF3B function biochemically by specific coIP of endogenous eIF4A3 and Y14 upon UPF3B-GFP pull-down (Figure 1G). Taken together, these results validate the GFP fusion proteins for iCLIP experiments.

The iCLIP protocol was optimized as detailed in Experimental Procedures and monitored to guarantee efficient and specific IP, RNA fragmentation, and cDNA library purification. In brief, we adjusted the IP conditions so that only the bait, but not the EJC proteins that do not directly bind the RNA (such as Y14), were detected (500 mM NaCl; Figure 1G). The distribution of the RNA fragments that coimmunoprecipitate with the proteins was adjusted to an optimal size range of 50–300 nt for iCLIP libraries (Figure S2A; Huppertz et al., 2014). After reverse transcription and amplification, the amplicons separated well (Figure S2B) and were purified from primer dimers prior to sequencing (Figure S2C).

Faithful Mapping of EJCs across the Transcriptome

We achieved highly specific and reproducible iCLIP reads with an excellent signal to noise ratio for all four tested EJC components (eIF4A3, BTZ, UPF3B, and RNPS1) and compared them to the two controls PTB and GFP (Figures S2D–S2F; Table S1). Each RBP was assessed by three biologically independent replicates, and RNA overamplification was avoided (Figure S2F) by including a barcoding system (König et al., 2010). Figure S2F shows that iCLIP experiments with the negative control (GFP) resulted in the expected high overamplification rate. This observation is in line with the low quantity of coimmunoprecipitated RNA fragments (Figure S2A) and validates GFP as a suitable background control. In comparison to GFP, the iCLIP reads of eIF4A3, UPF3B, RNPS1, and in particular BTZ, but not PTB, are highly enriched at splice junctions, demonstrating the specificity and reproducibility of the experiment (Figure S2G).

To identify potential differences of EJC binding to different regions of mRNAs, we next analyzed EJC binding to individual sites in a transcript-independent fashion. We used iCount (König et al., 2010; Sugimoto et al., 2012) together with our improved iCLIP analysis tools (Hauer et al., 2015) to detect pronounced binding sites (peaks) in the iCLIP data sets and calculated the percentage of these binding sites in 5′ UTRs, open reading frames (ORFs), 3′ UTRs, intronic or intergenic regions, or noncoding RNAs (ncRNAs), respectively (Figures 2A, S2H, and S2I). The biological specificity of these assignments is highlighted by the PTB iCLIP data set that reflects the known binding preference of this protein for intronic regions (Figure 2A; Han et al., 2014; Spellman and Smith, 2006; Xue et al., 2009). By contrast, EJC proteins display the expected predominant binding to ORFs (Figure 2A), a highly reproducible pattern between individual replicates (exemplified for BTZ in Figure 2B). Correlating the number of EJC subunit binding sites in the ORF and UTRs to the cumulative length of the respective regions demonstrated predominant binding of EJC proteins to coding regions and the preference for the 5′ UTR over the 3′ UTR, as expected (Figure 2C), as 5′ UTRs harbor about three times more introns than 3′ UTRs (Bicknell et al., 2012). The lower signal in the 5′

Figure 1. The Experimental HeLa Cell System Expressing Fully Functional EJC-GFP Fusion Proteins Is Suitable for iCLIP Experiments

(A) Titration of doxycycline demonstrated a concentration-dependent increase of the eIF4A3-GFP fusion proteins and that a concentration of 1 ng/ml doxycycline was best suited to achieve an expression close to the endogenous level. This image shows a representative immunoblot of three biologically independent experiments. Endogenous and recombinant eIF4A3 were stained concurrently with an α-eIF4A3 antibody.

(B) Representative immunoblot after siRNA treatment of three biologically independent experiments is shown. The endogenous and recombinant eIF4A3 were stained concurrently with an α-eIF4A3 antibody.

(C) Schematic drawing of three *SC35* mRNA isoforms adapted from Sureau et al. (2001). The grey boxes in *SC35WT* mRNA represent RNA regions that are spliced out in the other isoforms. The arrows show the position of the primers that were used for the amplification of the transcripts (Table S7).

(D and E) Upregulation of *SC35C* (D) and *SC35D* (E) transcripts after depletion of endogenous eIF4A3 and rescue of efficient NMD upon induction of the fusion protein with doxycycline.

(F) The expression of the NMD-insensitive *SC35WT* isoform did not change under the different conditions. The error bars represent SEM, and p values were calculated by one-way ANOVA with Dunnett's multiple comparison test (***p value < 0.001 with n = 3–5 independent biological experiments).

(G) CoIPs show that the EJC core proteins eIF4A3 and Y14 were stably associated with UPF3B and BTZ under salt concentrations of 150 and 250 mM NaCl and disassembled at 500 mM NaCl. Therefore, 500 mM NaCl was used for the subsequent iCLIP experiments. GFP, BTZ-GFP, and UPF3B-GFP were stained concurrently with an α-GFP antibody.

See also Figure S1 and Table S7.

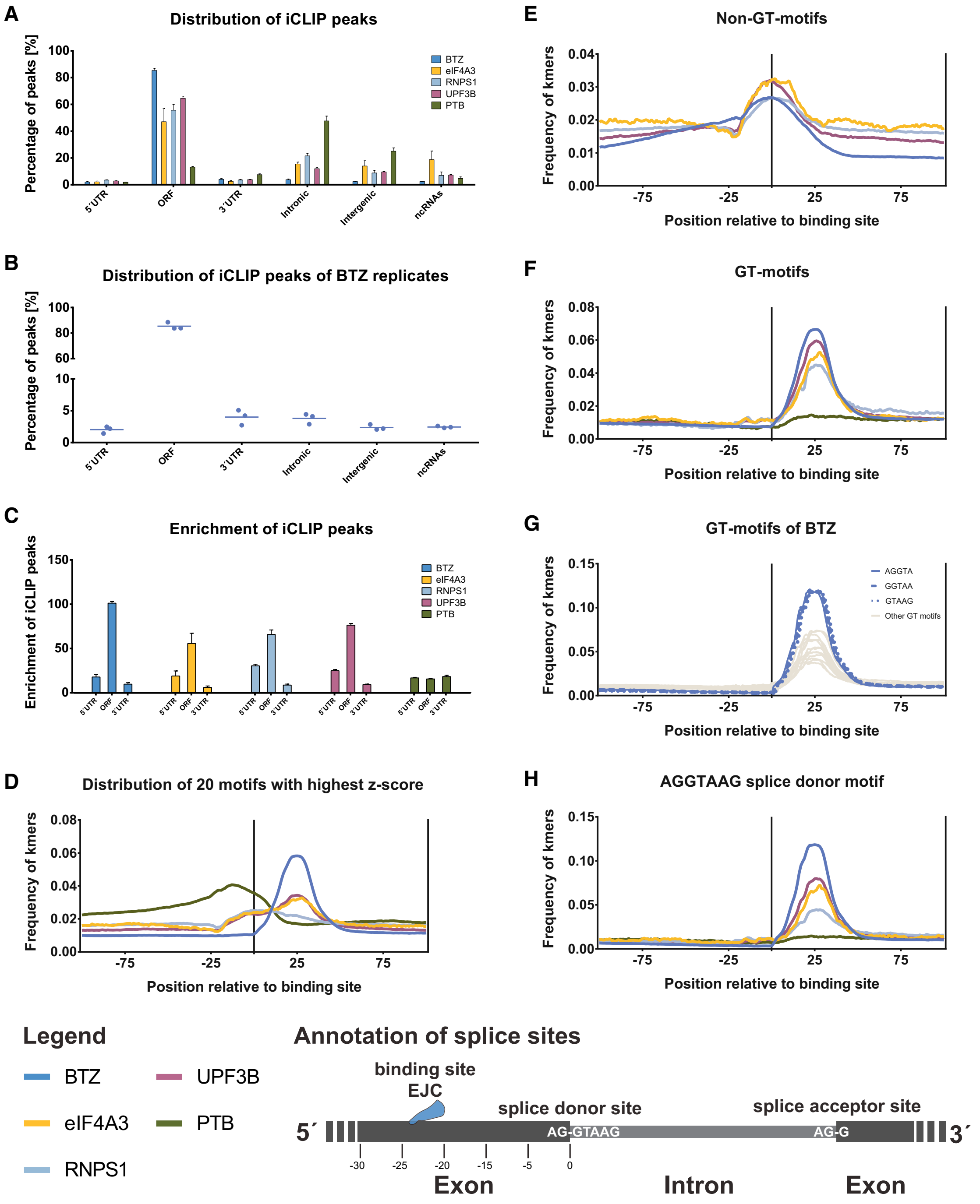

Figure 2. The Distribution of iCLIP Peaks Validates the Predominantly Sequence-Independent Deposition of EJC Proteins at Exonic Regions

(A) Relative distribution of peaks for all iCLIP data sets confirms that EJC components bind predominantly in the ORF.

(B) Distribution of BTZ peaks indicates the high reproducibility of the biological replicates; horizontal lines highlight the mean of three biologically independent BTZ iCLIP replicates.

(C) Enrichment of iCLIP peaks at mRNA regions calculated by dividing the number of iCLIP peaks by the cumulative length of indicated regions reveals that the ORF harbors by far the most EJC binding sites, followed by the 5′ UTR, and then the 3′ UTR regions. The error bars represent SEM of n = 3 independent biological experiments.

(D–H) The higher enrichment of the splice site signal compared to motifs within the reads confirmed the predominant sequence-independent deposition of the EJC. These graphs display the distribution of 5mer motifs around the binding site of the protein (illustrated as the vertical line in the middle of the plots).

(D) Average distribution of the 20 most enriched motifs (see also Table S2) relative to the binding site.

(E) Average distribution of 5mers that do not contain GT in their motif.

(legend continued on next page)

UTR compared to the ORF may arise from a proportion of EJC-free 5′ UTRs. The 5′ UTR is thought to be EJC-free when the first intron of the pre-mRNA is located more than 25 nt downstream of the start codon (Le Hir et al., 2000). In general, these analyses thus confirmed the expected and specific binding pattern of EJC components to the ORF.

The strong enrichment and high coverage of EJC binding sites in the ORF enabled the exploration of the postulated sequence-independent (Shibuya et al., 2004) as well as the low-stringency conserved sequence-dependent deposition of EJCs (Saulière et al., 2012; Singh et al., 2012). To discover potential sequence motifs, the region around iCLIP peaks was scanned for all possible combinations of five consecutive nucleotides (5mers). The specificity of the motif search was controlled by GFP and PTB data. The analysis of the GFP data set shows predominantly unspecific binding to the immunoprecipitated RNA indicated by low Z scores and "enriched motifs" that match recently published GFP background controls for PAR-CLIP (Table S2; Friedersdorf and Keene, 2014). By contrast, the 20 most highly enriched motifs for PTB reach higher Z scores and show the expected preference for polypyrimidine tracts (Table S2; Spellman and Smith, 2006). For the four EJC components, the average positional distributions of the 20 most frequent 5mers were slightly increased directly at the binding site, accompanied by a stronger enrichment downstream (Figure 2D). Interestingly, this motif profile for the EJC proteins could be separated into two distinct motif classes: one non-GT-containing (Figure 2E) and one GT-containing (Figure 2F) set of 5mers. The GT-containing 5mers cluster 25 nt downstream of the non-GT-containing ones (Figure 2F), perfectly matching the position of splice junctions. The distribution of all GT-motifs relative to the binding site of BTZ further revealed that three GT-motifs are even more highly enriched at this position (Figure 2G). Remarkably, these motifs concatenate to the canonical splice donor site 5′-AGGTAAG-3′, directly validating the canonical EJC position. The direct search for the canonical splice donor site motif revealed that all four EJC proteins, but not PTB, bind closely upstream of the splice site (Figure 2H).

By contrast, the weakly enriched non-GT-containing 5mers map to the EJC binding site, indicating minor sequence preferences (Figure 2E). This class of motifs includes 5′-GAAGA-3′ as the top motif for RNPS1, UPF3B, and BTZ (Table S2). For eIF4A3, CG-dinucleotides are enriched (Table S2), supporting a previous transcriptome-wide study of eIF4A3 using RNA-IP (RIP) (Singh et al., 2012), while it contrasts with published HITS-CLIP data (Saulière et al., 2012). Thus, EJC components exhibit a weak sequence preference, but the key determinant for EJC deposition across the transcriptome is its position vis-à-vis the splice junction (Figures 2E–2H).

mRNAs Are Differentially Marked by EJCs

We next tested the binding preferences of the EJC components to different types of RNA. We therefore directly compared EJC-bound RNAs to those bound by PTB using the differential analysis software edgeR (Robinson et al., 2010). We used the comparison to PTB to avoid bias by RNA abundance and because the PTB data set included >80% unique binding sites that are not shared with any of the EJC components (see restricted peaks to one RBP in Table S3).

This differential analysis separated mRNAs that are either enriched for EJC binding or for PTB binding at a false discovery rate (FDR) of <5% (Figures 3A and S3). We then performed gene ontology (GO) enrichment analysis for these two categories. Interestingly, this analysis revealed that all EJC subunits enrich on mRNAs that encode proteins that are involved in RNA processing, cell-cycle control, or chromosome organization. By contrast, PTB-bound RNAs are enriched for those encoding proteins with other functions such as electron and protein transport (Figures 3B–3F). Similar results were obtained for the EJC subunits when the differential analysis used GFP as a control (Figure S3).

Next, we selected common targets (n = 2,194) that are significantly enriched for BTZ binding sites in both differential analyses (compared to PTB and GFP) to reduce the number of false positive assignments. With this reliable subset, we were able to control the enrichment of EJC proteins on specific targets for mRNA abundance. Both, the RNA-sequencing (seq) and the BTZ iCLIP analyses show a high degree of reproducibility between independent biological replicates (Figures 3G and 3H). In general, BTZ iCLIP counts correlate well with RNA-abundance (Figure 3I). However, the majority of the targets that show the strongest enrichment in binding when compared to PTB (Figure 3A) also exhibit a higher BTZ iCLIP than RNA-seq signal (highlighted as red dots in Figure 3I). Remarkably, these mRNAs are distributed broadly across low and high abundance mRNAs in the cell. Thus, these data demonstrate: (1) a particularly pronounced association of EJCs with the mRNAs enriched in the functional categories outlined above and (2) independence of EJC association from RNA abundance per se (Figure 3I).

The Majority of Exons Harbor EJCs at the Canonical Deposition Sites

Next, we set out to analyze EJC composition in a systematic manner and thus classified exons in protein-encoding mRNAs into seven categories: (1) 5′ terminal exons; (2) constitutive exons present in all isoforms; (3) variant exons not present in all isoforms; (4) exons with alternative donor sites; (5) exons with alternative acceptor sites; (6) exons with both alternative donor and acceptor sites; and (7) 3′ terminal exons. We first considered all mRNAs and found that both constitutive and alternatively spliced exons are homogenously engaged with all four EJC components with the expected exception of 3′ terminal exons (Figure 4A; see Table S4 for statistics). To obtain a more detailed view for each individual exon, we plotted the binding sites of all four EJC components for each of the 123,585 constitutive exons as a single row in a heatmap (Figure 4B). As in the composite

(F) Average distribution of 5mers that do contain GT in their motif.

(G) The distribution of GT-containing 5mers in BTZ iCLIP experiments revealed three prominent motifs (AGGTA, GGTAA, and GTAAG).

(H) These most enriched GT-containing 5mers concatenate to the canonical splice donor site AGGTAAG, which is enriched in all EJC, but not in the PTB, libraries. See also Figure S2 and Tables S1–S3.

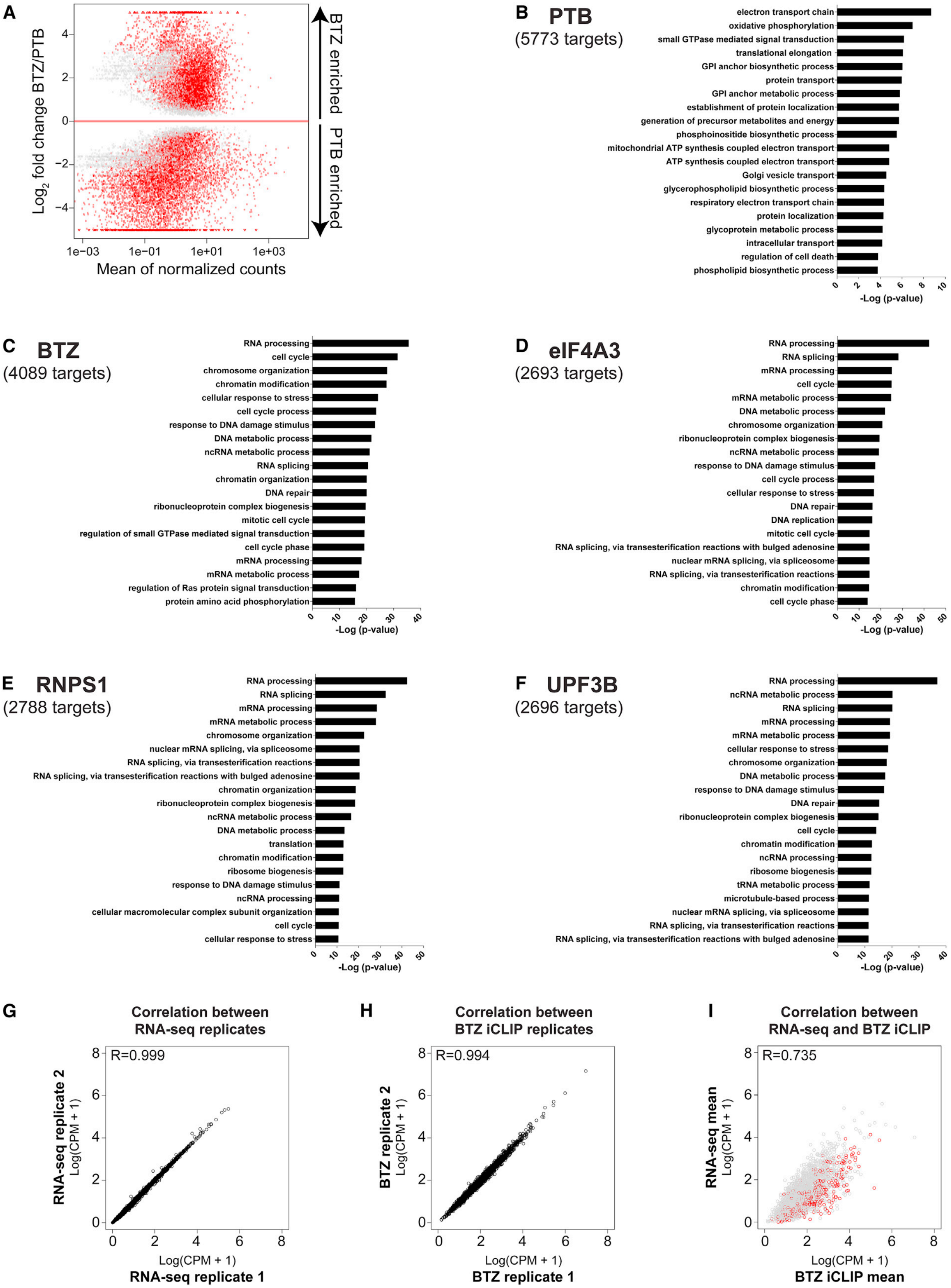

(legend on next page)

plots, BTZ exhibits the strongest signal, followed by eIF4A3, UPF3B, and RNPS1, whereas the PTB control shows the expected flat profile. In the heatmap, the length of each exon was set to a relative length of 100% and subsequently ordered by its absolute length. While the EJC signal displays the expected downstream shift with increasing length, the strength of the EJC signal does not depend on exon length, indicating that exon length does not determine the degree of EJC occupancy, which is consistent with the expectation that an exon generally binds only one EJC. However, very short exons show a considerably decreased EJC signal, fitting with earlier findings that exons shorter than 20 nt do not harbor an EJC (Gehring et al., 2009a).

These data thus indicate that >80% of fully assembled EJCs (as determined by coincident signals for all four tested subunits) are deposited at the expected sites 20–24 nt upstream of exon-exon junctions, contrasting with previous reports focusing on eIF4A3 binding alone, which suggested that 40%–50% of EJC recruitment occurs outside the 20–24 nt region (Saulière et al., 2012; Singh et al., 2012). Further, we directly show that binding clusters around the −24 position in a metaplot showing the iCLIP reads of BTZ at exon-exon junctions (Figure S4A).

BTZ Determines the Canonical EJC Deposition Site

To elucidate whether EJC composition is homogeneous or heterogeneous, we further enriched for the presence of bona fide EJC binding sites by defining high-confidence EJC deposition sites as those that bind at least two of the four EJC proteins (Figures 4C and 4D; see the Supplemental Information for filtering details). At least two of the four proteins could be detected on 59,521 sites across the whole transcriptome (Table S3). Remarkably, BTZ binding marks 53,812 of these sites, indicating that BTZ peaks offer the best single EJC protein approximation of bona fide EJC deposition sites (Table S3). Figure 4C illustrates the increasing density of BTZ deposition sites for three subsets of reads: (1) raw reads; (2) reads in peaks; and (3) high-confidence reads in peaks that overlapped with at least one of the other EJC proteins (eIF4A3, UPF3B, and RNPS1).

The interpretation of BTZ representing an essential EJC component and a valid marker for EJCs is reinforced by examination of common peaks between the other three EJC proteins (eIF4A3, UPF3B, and RNPS1) not coinciding with BTZ peaks. The average profiles of such peaks are enriched toward the 5′ rather than 3′ end of exons, thus likely representing RNP complexes that do not function as EJCs (Figure 4D).

Since the composition of the EJCs displays remarkable homogeneity, we took an in-depth look at mRNAs that have been described as endogenous targets of alternative NMD pathways that are differentially sensitive to the depletion of different EJC core proteins including RNPS1, BTZ, and UPF3 (Chan et al., 2007, 2009; Gehring et al., 2005; Wang et al., 2014). One hypothesis to explain this differential sensitivity posits that the subunit composition of EJCs may differ on these transcripts. However, we identified high-confidence binding sites on more than 100 exons belonging to different mRNA targets of distinct NMD pathways (Table S5). For instance, the *SC35* mRNA described to be BTZ-sensitive, but RNPS1-insensitive, has binding sites for both proteins (Figure 5C). In addition, the *EPAS1* mRNA that was mainly upregulated after RNPS1 depletion (Gehring et al., 2005) shows high BTZ marks in our iCLIP data (Figure S4). Thus, differences of EJC composition do not explain the previously noted differential cofactor requirements for branch-specific NMD of such transcripts. Therefore, we hypothesize that posttranslational modifications and/or additional subunits are involved, although we cannot formally exclude that differences between the different strains of HeLa cells and growth conditions may have contributed.

An important finding of this work is that most exon junctions are bound by all four tested EJC proteins (as shown by the similar binding pattern in the heatmaps for the EJC components in Figure 4B), although some mRNAs are more prevalent in the BTZ and RNPS1 iCLIP data sets and were confirmed by RIP experiments (Figure S5). Interestingly, we observe binding of individual EJC subunits, in particular RNPS1, to non-EJC sites. For RNPS1, binding to non-EJC sites is indicated by the motif analysis because, in contrast to the other EJC components, RNPS1 bound fragments show a weaker enrichment for the splice site donor motif when the 20 top motifs are considered (see Table S2 and Figure 2D). Moreover, RNPS1 binding is less restricted to the 3′ end of exons when compared to the other three EJC components (see Figures 4A and 4B). This finding is likely explained by the known non-EJC functions of RNPS1 (Mayeda et al., 1999; Michelle et al., 2012; Murachelli et al., 2012).

In conclusion, the analysis of high-confidence EJC binding sites reveals BTZ as a reliable marker for bona fide EJCs on a transcriptome-wide level (Figure 4E).

The EJC Signal Is Significantly Increased on Alternatively Spliced Exons

We therefore focused on those 2,194 mRNAs with particularly strong BTZ signals (see Figure 3). Because the GO terms for

Figure 3. Binding of All EJC Components Is Highly Enriched to Transcripts Coding for RNA Processing Proteins

(A–F) The red dots show significant mRNA targets either up- or downregulated after differential analysis using edgeR controlled by the Benjamini-Hochberg procedure with an FDR <0.05. The targets below the red line provided a higher PTB signal, whereas targets above the red line exhibited a higher BTZ signal (A). The targets below the red line were used for GO enrichment analysis of PTB (B). All EJC iCLIP data sets were compared to PTB, and the targets with a $\log_2$ fold change >0 (above red line) were analyzed for GO enrichment for BTZ (C), eIF4A3 (D), RNPS1 (E), and UPF3B (F).

(G–I) Enriched EJC occupancy does not correlate with mRNA abundance. These plots display the correlation of iCLIP and RNA-seq data using the 2,194 common targets that were significantly enriched in BTZ iCLIP in both differential analyses (compared to PTB and GFP).

(G and H) The RNA-seq (G) and BTZ iCLIP (H) replicates were highly reproducible.

(I) Relationship between the mean count of all three RNA-seq and BTZ iCLIP libraries. The red dots highlight mRNA targets for BTZ with a $\log_2$ fold change >3 compared to PTB in the differential analysis using edgeR and thus indicate mRNAs that are particularly strongly bound by BTZ. The specificity of these highly occupied mRNAs is demonstrated by the finding that these mRNAs were distributed across transcripts with high and low expression levels as measured by RNA-seq (CPM and R = Pearson's correlation coefficient).

See also Figure S3.

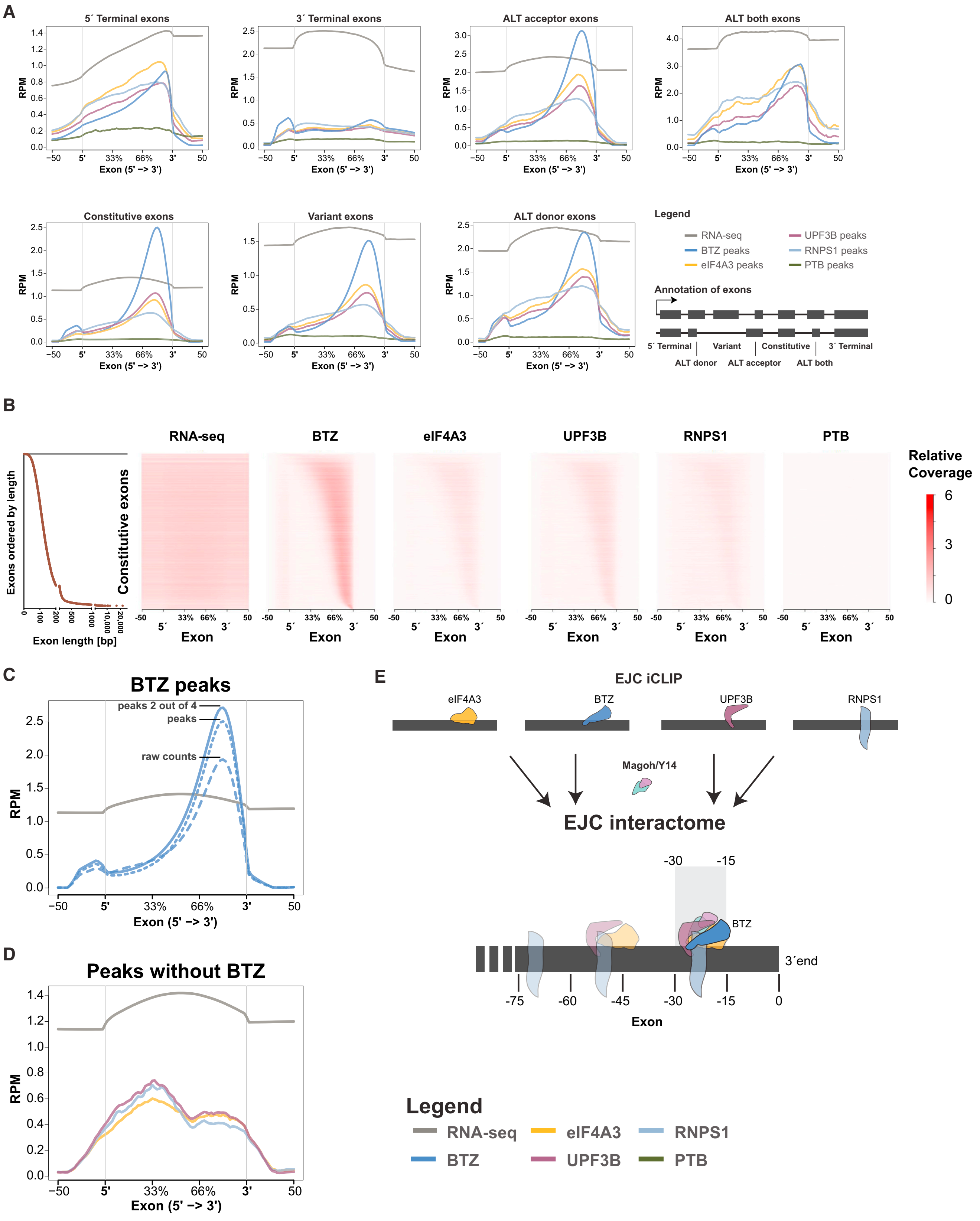

(legend on next page)

RNA processing, cell cycle, and chromosome organization in RNAs with strong BTZ signal (Figure 3C) were recently reported to be highly enriched for alternative splicing events (Pimentel et al., 2014), we next set out to analyze the relationship between alternative splicing and EJC deposition. Notably, this set of mRNAs shows strongly increased BTZ binding to variant and alternatively spliced exons when compared to all mRNAs (Figures 5A and S6A). Moreover, the mRNAs with strong BTZ occupancy are enriched for GO terms to be alternatively spliced (1,230/2,194) when compared to all mRNAs (7,458/19,113, $p = 6.7 \times 10^{-72}$). These findings indicate that BTZ binding, and by implication the recruitment of EJCs (see above), plays a particularly important role for alternatively spliced mRNAs.

The enrichment of BTZ binding on alternatively spliced exons is exemplified by *SRSF2* (*SC35*). This example also highlights the importance of using libraries with a high level of complexity and deep coverage of reads for the reliable detection of rare interactions between a RBP and its binding transcripts in iCLIP analyses (Sims et al., 2014), such as those occurring on alternatively spliced exons of NMD-sensitive mRNA isoforms. With the highly complex BTZ iCLIP data set, we detect not only the interactions on the abundant exons, but also disproportionately strong signals on low-abundance exons. We identify several hundred of low-abundance exons with an EJC signal that is at least twice as strong as the RNA-seq (Table 1), such as the exons of the rare NMD-sensitive *SC35C* isoform (see Figure 5B, red box highlighting exon 3), indicating that the EJC preferentially binds the variant exon of this transcript. In general, NMD-sensitive transcript isoforms are rapidly degraded, thus defining a suitable class of mRNAs for an analysis of iCLIP signals on low abundance exons. In addition, there are other low-abundance mRNAs, exemplified by *GADD45A*, which are not degraded by NMD with an average RNA-seq coverage in HeLa cells below 1 count per million (CPM; Figure S6B). Nevertheless, the BTZ iCLIP signal is strong for the two internal exons in *GADD45A*, which indicates that BTZ iCLIP data can detect EJC deposition sites with great sensitivity even in mRNAs with low expression levels (see Table 1). As a control for the specificity of EJC marks, which should be only present on mRNAs transcribed from intron containing genes, the PTB iCLIP data were used. For example, the RNA derived from the intronless *ZXDB* gene, which shows the expected lack of EJC-signals, clearly reflects PTB binding to the 3′ UTR of this transcript (Figure S6C), possibly reflecting the role of PTB in regulating 3′ end processing (Danckwardt et al., 2011; Millevoi et al., 2009).

With these data in hand, we set out to identify exons that have previously not been annotated. In an analysis including only exons containing canonical splice sites, and binding of BTZ plus one of the other EJC proteins, we identified 32 high confidence candidate exons that were not previously annotated in the Ensembl, RefSeq, or University of California Santa Cruz (UCSC) gene databases. We validated our bioinformatical approach to find candidate exons by manually checking these 32 regions in the genome browser and thus confirmed 28/32 exons in intergenic and intronic regions (Figures 5B, 5C, and S7A–S7C; Table S6). Thus, the analysis of this "deep" BTZ iCLIP data set enabled detailed insights into expressed mRNA-isoforms and even previously unknown transcripts.

Low EJC Occupancy of mRNAs Encoding Ribosomal Proteins

Ranking constitutive exons (from Figure 4B) by their EJC occupancy in relation to exon abundance (Figure 6A), we noticed four main categories: (1) exons that are highly abundant and show a weak iCLIP signal; (2) exons that are expressed and harbor a corresponding EJC signal; (3) exons that are not expressed and therefore do not have an EJC signal; and (4) exons that are weakly expressed but show enrichment of EJC binding. From the 123,585 constitutive exons in the database, 79,840 were expressed in HeLa cells above a threshold of 10 reads per exon. An EJC signal represented by BTZ was found on 60,793 of these expressed constitutive exons, if a conservative threshold of at least 10 reads per expressed exon was assigned, indicating that most exons harbor an EJC.

We further selected for bona fide EJC sites that bind at least two EJC proteins and analyzed the categories of the 123,585 constitutive exons according to their strength of BTZ/EJC binding (Figure 6B). To our surprise, more than half of the top 50 highly expressed exons with low EJC occupancy (category 1) are mRNAs coding for ribosomal proteins (Figure 6B). In general, the exons of mRNAs coding for ribosomal proteins show low BTZ/EJC binding (Figures 6C and 6D). When considering binding

Figure 4. High-Confidence EJC Binding Sites Reveal that BTZ Is an Essential EJC Component at the Canonical Deposition Site

(A) Internal exons are predominantly bound by EJC components and constitutive exons display the strongest BTZ signal relative to their abundance. The average profile of reads covering exons are plotted for: (1) 5′ terminal exons; (2) 3′ terminal exons; (3) constitutive exons present in all isoforms; (4) variant exons not present in all isoforms; (5) exons with alternative acceptor sites (ALT acceptor); (6) exons with alternative donor sites (ALT donor); and (7) exons with both alternative donor and acceptor sites (ALT both) using ngsplot software (Shen et al., 2014).

(B) Histogram on the left hand side shows the length of the constitutive exons and is aligned to the heatmaps showing RNA-seq and iCLIP coverage across individual exons ordered by their length. The length of the exons can be extracted from the histogram. This image confirms that most exons harbor an EJC at the 3′ end and demonstrate that the EJC signal strength is independent of exon length. The color key represents the signal strengths of the RNA-seq and iCLIP data.

(C) Average coverage profiles across constitutive exons for BTZ iCLIP: (1) raw reads; (2) reads in peaks; and (3) reads in peaks that overlap with at least one of the other EJC proteins (eIF4A3, UPF3B, and RNPS1) are restricted to the 3′ end of exons. The peak detection and filtering approaches increased the BTZ signal.

(D) By contrast, the average exon profiles for eIF4A3, UPF3B, and RNPS1 iCLIP binding sites that were not determined concurrently by BTZ binding sites were absent of an EJC signal close to the 3′ end of exons suggesting that non-canonical binding sites do not contain the fully assembled EJC. The profiles are plotted as read CPM mapped reads (RPM).

(E) The integrated analyses over four distinct components of the EJC revealed that BTZ determines the position of fully assembled EJCs to the canonical deposition sites at 15–30 nt upstream of exon-exon junctions. Non-canonical depositions sites of EJC proteins (alone or in subcomplexes) located in other regions of the exon are less common and do not contain BTZ.

See also Figure S4 and Tables S4–S6.

OPEN
ACCESS
CellPress

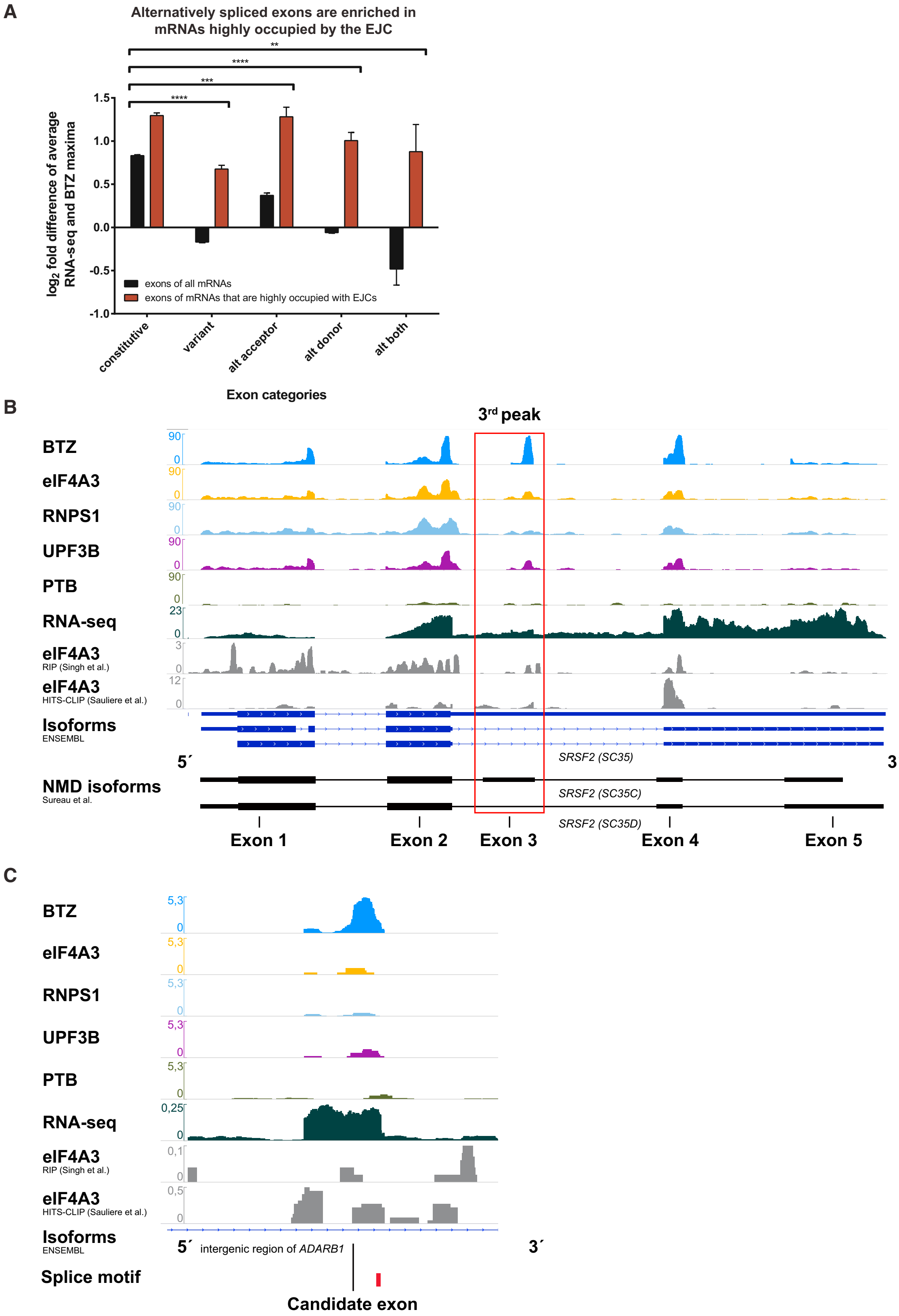

(legend on next page)

OPEN ACCESS
CellPress

Table 1. Binding of High-Confidence EJCs Identifies Low Abundance Exons and Previously Non-annotated Exons

Exon Type	Number of Exons with Signal of BTZ and One Other EJC Protein >1 RPM and RNA-Seq <0.5 RPM	% of All Annotated Exons
altBoth	208	4.8
altDonor	415	3.7
altAcceptor	668	4.0
Variant	935	1.8
Constitutive	2,904	2.3
	Number of Non-annotated Exons with Signal of BTZ >2 RPM and One Other EJC Protein and Canonical Splice Site Donor Motif	Validated
Candidate	32	28 (90%)

of the other EJC components, these can be found to bind to these exons with an equal distribution throughout their entire length, thus not representing bona fide binding as part of the EJC. We considered whether the lack of EJCs on these RNAs may be explained by the ribosomes efficiently removing EJCs because mRNAs of ribosomal proteins contain a terminal oligopyrimidine (TOP) in their 5′ UTR, which supports their efficient translation in actively proliferating cells (Meyuhas, 2000; Ruvinsky and Meyuhas, 2006). However, translation was inhibited by cycloheximide prior to iCLIP. Moreover, the average distribution of the raw reads of the two previously published transcriptome-wide studies of eIF4A3 (Saulière et al., 2012; Singh et al., 2012) also do not show a 3′ enrichment of binding on this type of exon (Figure 6D). We further considered whether the low abundance of EJC signals might be explained by the high stability of mRNAs coding for ribosomal proteins (Schwanhäusser et al., 2011). In this scenario, a high proportion of these RNA molecules may have already been translated and thus have lost their EJCs before translation has been blocked. Such a mechanism would predict that other mRNAs with long half-lives should also be found among those with a low abundance of bound EJC components. In contrast to this prediction, other stable and highly expressed mRNAs, such as actin mRNA, show strong EJC signals. We also determined whether the presence of a TOP motif might define a subset of mRNAs with low EJC abundance. While TOP mRNAs are indeed enriched in the category showing a weak iCLIP signal (Figure 6E), some exons of these mRNAs showed a canonical EJC signal (Figures S7D and S7E), indicating that the TOP motif per se does not define whether EJCs are efficiently recruited.

In conclusion, the analysis of high-confidence EJC binding sites by in vivo RNA binding studies of four different EJC proteins shows that approximately 80% of expressed constitutive exons harbor a detectable EJC signal at the canonical deposition site upstream of the 3′ end of exons. Surprisingly, exons coding for ribosomal proteins (and other TOP mRNAs) show a remarkably low occupancy of EJC components, which suggests a specific difference in RNA processing of this type of transcript. By contrast, an enrichment of the EJC signal was observed for mRNAs that derive from alternative spliced pre-mRNAs in general, including mRNAs coding for proteins associated with RNA processing in particular (McGlincy and Smith, 2008; Saltzman et al., 2008). The differential recruitment of EJCs might help the RNA-processing machinery to mark different mRNA isoforms. Moreover, the composition of the EJC is more homogeneous than previously assumed. Therefore, peripherally associated EJC proteins and/or posttranslational modifications likely play a role in determining the fate of mRNAs in alternative NMD pathways.

DISCUSSION

EJCs are involved in several stages of mRNA metabolism and are therefore key effectors of protein expression. In particular, EJC deposition can enhance the efficiency of NMD. Since inhibition of NMD stabilizes up to 10% of mRNAs (Mendell et al., 2004; Tani et al., 2012; Wittmann et al., 2006; Yepiskoposyan et al., 2011), one may hypothesize that the distribution and composition of EJCs across the transcriptome varies between different mRNA transcripts and in particular between targets of alternative NMD pathways (Chan et al., 2007, 2009; Gehring et al., 2005; Huang et al., 2011). The integrated analysis of RNA binding of four EJC components in vivo by iCLIP reported here enabled us to define high-confidence EJC binding sites defined by cooccurrence of at least two EJC subunits. The resulting landscape of bona fide EJCs across the transcriptome indicates that EJCs are recruited to most exon junctions corroborating data from IP experiments with the EJC protein eIF4A3 (Saulière et al., 2012; Singh et al., 2012).

Previously reported analyses of eIF4A3 binding indicated that 40%–50% of the binding sites of this protein are located at non-canonical regions outside of the known EJC location 20–24 nt upstream of exon-exon junctions (Saulière et al., 2012; Singh et al., 2012). Moreover, the proteomic analysis of EJCs purified from HEK293 cells showed that BTZ is substoichiometric to eIF4A3, indicating either: (1) that BTZ may not be present in all EJCs or (2) that eIF4A3 has additional functions outside the

Figure 5. Binding of EJC Components Is Highly Enriched at Alternatively Spliced Exons in Transcripts with High EJC Occupancy and Enables Detection of Low-Abundance NMD-Sensitive mRNA Isoforms

(A) We used the RNA-seq and BTZ maxima as shown in Figure 4 (see also Table S4) to calculate a $\log_2$ fold difference for both all mRNAs and mRNAs that are highly occupied by EJCs. The iCLIP/RNA-seq ratio is enriched for alternatively spliced exons in RNAs that are highly occupied by EJCs (see Figures 3G–3I) compared to all mRNAs.

(B) Genome browser view of *SRSF2* (*SC35*) gene reveals EJC iCLIP peaks on exons corresponding to NMD-sensitive *SC35C* and *SC35D* mRNA isoforms. The NMD-insensitive *SC35WT* isoform is displayed in the Ensembl genes track as the upper isoform. The red box highlights the variant exon 3.

(C) Candidate exon in the intronic region of the mRNA *ADARB1*. The track range displays CPM and was adjusted to the highest iCLIP signal obtained in the iCLIP libraries of this study in each genome browser view. The signals of the RNA-seq and literature data were not adjusted. The literature data were obtained from RIP (Singh et al., 2012) and HITS-CLIP (Saulière et al., 2012) of eIF4A3.

See also Figures S4 and S6.

OPEN
ACCESS
CellPress

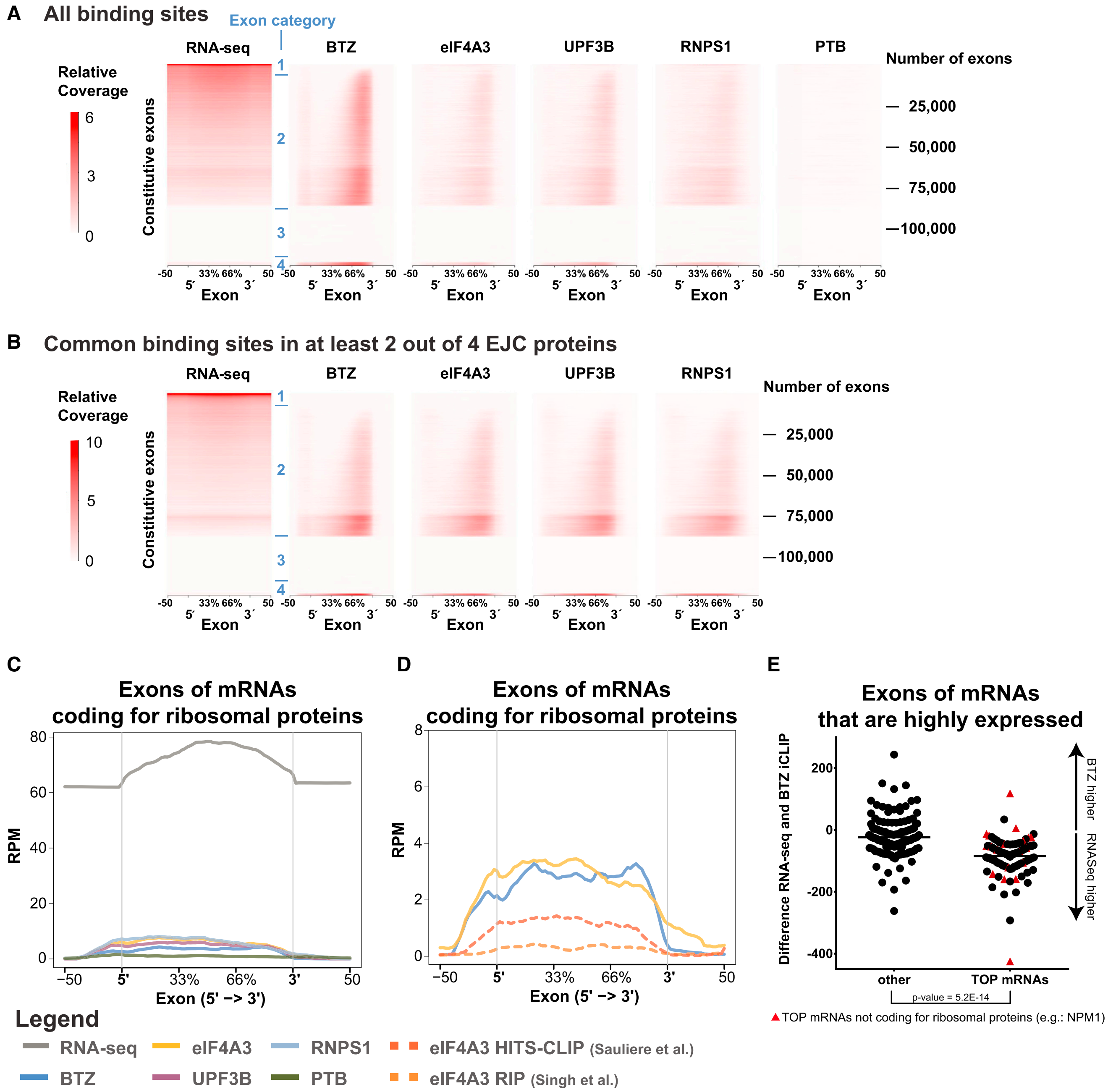

Figure 6. EJC Binding Is Underrepresented on mRNAs Coding for Ribosomal Proteins

(A) Heatmaps of individual exons clustered by the differences between the RNA-seq and the iCLIP profile uncovered four different exon categories: (1) exons that are highly abundant and show a weak iCLIP signal; (2) exons that are expressed and harbor a corresponding EJC signal; (3) exons that are not expressed and therefore do not have an EJC signal; and (4) exons that are weakly expressed, but show enrichment of EJC binding.

(B) The same clustering was performed only with reads in binding sites that were determined by at least two out of four EJC proteins. Each row of the heatmap represents one of the 123,585 constitutive exons of the human reference genome. The color key represents the signal strengths of the RNA-seq and iCLIP data.

(C and D) EJC occupancy is highly diminished in mRNAs coding for ribosomal proteins. Average coverage profiles across canonical exons of ribosomal protein coding genes lack an increased EJC signal at the 3′ end of the exon for all EJC proteins (C) including published eIF4A3 data from RIP (Singh et al., 2012) and HITS-CLIP (Saulière et al., 2012) of eIF4A3 (D). The average profiles are plotted as read CPM mapped reads (RPM) calculated using peak data (C) or raw counts (D).

(E) Exons of highly expressed mRNAs (>40 CPM) that do not belong to the TOP mRNA class show a higher BTZ signal analyzed by Welch two sample t test.

See also Figures S4 and S7.

EJC core (Singh et al., 2012). Here, we provide direct evidence in favor of the latter scenario and demonstrate that fully assembled EJCs generally bind to the expected sites (see Figure 4).

Furthermore, our analyses show that the subunit composition of EJCs is surprisingly homogeneous. This result is remarkable because linking the EJC and the bound RNAs to diverse downstream cellular pathways thus appears to be independent of the composition of the core complex. These links are likely conferred by the more peripheral EJC components. Considering the structural data of the EJC-UPF3B subcomplex with short synthetic RNAs (Buchwald et al., 2010; Melero et al., 2012), one might have expected that the protein UPF3B does not bind RNA directly. By contrast, we demonstrate that all four EJC proteins directly bind to the RNA, as expected from RNA interactome data (Castello et al., 2012). Specifically, the majority of RNPS1 and UPF3B binding sites are shared by the EJC core proteins BTZ and eIF4A3. All four EJC proteins are bound to the RNA in an area of 15–30 nt upstream of the 3′ end of exons and thus map the proposed EJC binding site to a defined narrow range on a transcriptome-wide scale. This binding of multiple protein subunits of the EJC within a confined binding space may appear surprising. However, crystallography of the EJC core with BTZ and eIF4A3 revealed that both proteins could bind simultaneously in a region of six nucleotides (Andersen et al., 2006; Bono et al., 2006). Thus, there is likely sufficient space for two additional proteins. Further, our coIP experiments (Figure 1) and previous studies (Singh et al., 2012) show that the EJC is bound to the RNA as a stable complex. While PTB binds to unique sites that are mostly not shared with any of the EJC components (Table S3), eIF4A3 and UPF3B share 93% and 88% of the binding sites. Similarly, BTZ and RNPS1 accounted for fewer unique peaks (54% and 39%) compared to PTB. Importantly, unique peaks for BTZ correspond to classical EJC positions, reflecting the depth of the BTZ library. This finding is consistent with data showing that BTZ and the other EJC core factors can associate with pre-mRNAs in the nucleus (Björk et al., 2015), although BTZ is also abundant in the cytoplasm (Gehring et al., 2009a).

We suggest BTZ to serve a particularly important role as an EJC protein because: (1) it is bound almost exclusively to the canonical EJC deposition site and (2) it is present at more than 90% of the high-confidence EJC binding sites (see Table S3). As previously reported, BTZ can trigger important downstream pathways such as NMD (Gehring et al., 2005) and can stimulate translation (Chazal et al., 2013). In conclusion, our integrated approach to determine the transcriptome-wide binding of four EJC subunits identified high-confidence EJC deposition sites across the transcriptome. We propose that fully assembled EJCs are restricted to the canonical deposition sites at the 3′ end of mRNA exons in the majority of cases (see model in Figure 4E).

EJCs Are Enriched at Alternatively Spliced mRNAs and Underrepresented on mRNAs Encoding Ribosomal Proteins and Other TOP mRNAs

It is remarkable that RNAs with strong EJC signals are enriched for alternatively spliced exons. Previous studies noted preferential binding of EJC proteins to transcripts coding for RNA processing proteins (Saulière et al., 2012; Singh et al., 2012; Wang et al., 2014), a subgroup of mRNAs that are frequently alternatively spliced (McGlincy and Smith, 2008; Pimentel et al., 2014; Saltzman et al., 2008). In contrast to earlier studies, we directly show that the enrichment of EJCs on alternatively spliced mRNAs is mainly due to the increased binding of the EJC to the alternatively spliced and variant, but not to the constitutive exons (Figure 5A).

Why may alternatively spliced mRNAs display increased EJC occupancy? Although PTCs introduced by alternative splicing could prevent the ribosome from interacting with downstream EJCs, this explanation is unlikely because translation was inhibited resulting in an equal distribution of EJCs across the transcripts (Figure S7F). Potentially, proteins involved in alternative splicing affect the splicing apparatus in a way that promotes EJC deposition.

By contrast to alternatively spliced exons, we found that mRNAs bearing the TOP motif at their 5′ ends display strongly reduced EJC binding at most exons across all sites without bias for the 5′ exon (Figures 6C, 6D, S7D, and S7E). This feature of TOP mRNAs does not appear to be governed by their typically long half-life or their high rate of translation (Schwanhäusser et al., 2011) because we found other long-lived RNAs and other particularly actively translated RNAs to be well covered by EJCs. Moreover, some individual exons of TOP RNAs display a similar EJC density as other constitutively spliced mRNAs (Figure S7D).

EXPERIMENTAL PROCEDURES

Stable HeLa Cell Lines

Stable and inducible HeLa cell lines expressing GFP-tagged proteins at a level comparable to the endogenous counterparts were generated (see the Supplemental Experimental Procedures).

NMD Efficiency Assay

NMD efficiency in HeLa cells was measured by a chemiluminescence NMD reporter (Figure S1A; Boelz et al., 2006).

siRNA Knockdown and Complementation Assay

The siRNAs targeted the 3′ UTR of the endogenous EJC factors. The depletion of endogenous proteins and induction of recombinant proteins were monitored by immunoblotting and qRT-PCR (see the Supplemental Experimental Procedures; see Table S7 for siRNA and primer sequences).

Immunoblots

Immunoblot conditions (e.g., antibodies) are provided in the Supplemental Experimental Procedures and in Table S7.

iCLIP

Prior to iCLIP, HeLa cells were treated with 100 μg/ml cycloheximide for 1 hr to inhibit translation. For this study, the original iCLIP protocol (König et al., 2010) was used with some modifications (see the Supplemental Experimental Procedures).

RIP

RIP followed the IP protocol for iCLIP, but omitted the UV crosslinking and the steps of RNA and protein digestion (see the Supplemental Experimental Procedures).

RNA-Seq

RNA was isolated with TRIzol from 50 μl of the cell lysate of three biological replicates used for iCLIP experiments. Total RNA (1 μg) was diluted to a final

volume of 8 μl with H2O, mixed with 1 μl of 10× reaction buffer and 1 μl of DNase I (1 U/μl) and incubated for 15 min at 37°C. RNA was purified according to the RNeasy Kit and rRNA depleted. Strand specific libraries were prepared and sequenced paired-end with 50 bp on an Illumina HiSeq2000.

Mapping and Counting of Reads

The iCLIP and RNA-seq reads were mapped to the human reference genome (assembly GRCh37, as provided by Ensembl 75) and an exon junction database with an overhang of 99 nt using STAR v2.3.0 (Dobin et al., 2013). A total mismatch rate of 2 was allowed (outFilterMismatchNmax 3 and outFilterMismatchNoverLmax 0.12). If reads map equally well to two different locations, an alignment was randomly selected (default in STAR). iCLIP and RNA-seq reads were counted for each gene in GRCh37 with HTSeq (v0.6.1) and python (v2.7.5) using the sorted and random barcode evaluated reads.

Genome Browser

The iCLIP reads were visualized in IGV (Robinson et al., 2011; Thorvaldsdóttir et al., 2013), together with literature data of eIF4A3 HITS-CLIP (Saulière et al., 2012) and RIP (Singh et al., 2012). RIP data (accession number: SRX189574) was converted to human genome 19 with the liftOver tool (UC Santa Cruz).

Peak Analysis

To identify peaks, the iCount algorithm was applied (König et al., 2010; Sugimoto et al., 2012), together with our improved iCLIP analysis tools (Hauer et al., 2015). We used a flank size of 15 nt, 100 random permutations, and an FDR of <5% to identify read clusters (Yeo et al., 2009).

The enrichment factors of iCLIP peaks in the ORF and the UTRs were calculated by dividing the number of peaks by the cumulative length of those regions.

For high-confidence EJC binding site analysis, peaks that had at least an overlap of 1 nt between two different iCLIP experiments were merged into a single peak and filtered by GFP reads (see Supplemental Experimental Procedures).

Motif Analysis

Genomic segments next to the detected iCLIP peaks were scanned for all possible 5mer. All occurring 5mers 40 nt up- and downstream of a peak midpoint were counted. As for the detection of peaks, reference data were generated by randomly shuffling the iCLIP positions 100 times within the corresponding genomic segments. The positional distribution was normalized by the mean random score of the detected 5mers in a region 100 nt up- and downstream of the peak. The 5mer is centered at the reported positions.

Distribution of EJCs

The distribution of EJCs over the whole length of different mRNAs and exons was calculated using the ngs.plot software (Shen et al., 2014) with Ensembl 75 annotation. To visualize the differences between RNA-seq and EJC iCLIP signals, the exons were ranked with the parameter "-GO diff".

Candidate Exons

We searched for EJC binding sites that: (1) did not map to annotated exons (Ensembl 75, RefSeq, and UCSC databases); (2) had a BTZ signal of >2 reads per million (RPM) that was confirmed by at least one other overlapping EJC protein; and (3) contained the canonical splice site donor motif AGGTAAG or AGGTGAG in close proximity (a maximum of 25 nt).

Differential Binding and GO Enrichment

Raw counts were used to analyze differential binding of RBPs to mRNAs by edgeR (v3.8.5) with a Benjamini-Hochberg calculated adjusted p value < 0.05 (Robinson et al., 2010). The enriched ontology terms were determined using the Database for Annotation, Visualization, and Integrated Discovery with whole genome background as default (DAVID, v6.7; Huang et al., 2009).

ACCESSION NUMBERS

The accession number for the FASTQ files of iCLIP and RNA-seq libraries reported in this paper is ArrayExpress: E-MTAB-4215.

SUPPLEMENTAL INFORMATION

Supplemental Information includes Supplemental Experimental Procedures, seven figures, and seven tables and can be found with this article online at http://dx.doi.org/10.1016/j.celrep.2016.06.096.

AUTHOR CONTRIBUTIONS

Conceptualization, C.H., M.W.H., and A.E.K.; Methodology, C.H. and A-.M.A.; Software and Formal Analysis, C.H., T.S., and T.C.; Visualization and Validation, C.H.; Investigation, C.H., J.S., and I.H.; Writing - Original Draft, C.H.; and Writing - Review & Editing, M.W.H. and A.E.K.

ACKNOWLEDGMENTS

We thank the EMBL Genomics Core Facility for high-throughput sequencing and especially V. Benes for advice in library preparation. T.C. acknowledges support by the Slovenian Research Agency (J7-5460). M.W.H. and A.E.K. acknowledge support by the Deutsche Forschungsgemeinschaft in the context of the program grants FOR 855 and SFB 1036.

Received: March 14, 2016
Revised: May 16, 2016
Accepted: June 29, 2016
Published: July 28, 2016

REFERENCES

Andersen, C.B., Ballut, L., Johansen, J.S., Chamieh, H., Nielsen, K.H., Oliveira, C.L., Pedersen, J.S., Séraphin, B., Le Hir, H., and Andersen, G.R. (2006). Structure of the exon junction core complex with a trapped DEAD-box ATPase bound to RNA. Science *313*, 1968–1972.

Ballut, L., Marchadier, B., Baguet, A., Tomasetto, C., Séraphin, B., and Le Hir, H. (2005). The exon junction core complex is locked onto RNA by inhibition of eIF4AIII ATPase activity. Nat. Struct. Mol. Biol. *12*, 861–869.

Bicknell, A.A., Cenik, C., Chua, H.N., Roth, F.P., and Moore, M.J. (2012). Introns in UTRs: why we should stop ignoring them. BioEssays *34*, 1025–1034.

Björk, P., Persson, J.O., and Wieslander, L. (2015). Intranuclear binding in space and time of exon junction complex and NXF1 to premRNPs/mRNPs in vivo. J. Cell Biol. *211*, 63–75.

Boelz, S., Neu-Yilik, G., Gehring, N.H., Hentze, M.W., and Kulozik, A.E. (2006). A chemiluminescence-based reporter system to monitor nonsense-mediated mRNA decay. Biochem. Biophys. Res. Commun. *349*, 186–191.

Bono, F., Ebert, J., Lorentzen, E., and Conti, E. (2006). The crystal structure of the exon junction complex reveals how it maintains a stable grip on mRNA. Cell *126*, 713–725.

Buchwald, G., Ebert, J., Basquin, C., Sauliere, J., Jayachandran, U., Bono, F., Le Hir, H., and Conti, E. (2010). Insights into the recruitment of the NMD machinery from the crystal structure of a core EJC-UPF3b complex. Proc. Natl. Acad. Sci. USA *107*, 10050–10055.

Castello, A., Fischer, B., Eichelbaum, K., Horos, R., Beckmann, B.M., Strein, C., Davey, N.E., Humphreys, D.T., Preiss, T., Steinmetz, L.M., et al. (2012). Insights into RNA biology from an atlas of mammalian mRNA-binding proteins. Cell *149*, 1393–1406.

Chan, W.K., Huang, L., Gudikote, J.P., Chang, Y.F., Imam, J.S., MacLean, J.A., 2nd, and Wilkinson, M.F. (2007). An alternative branch of the nonsense-mediated decay pathway. EMBO J. *26*, 1820–1830.

Chan, W.K., Bhalla, A.D., Le Hir, H., Nguyen, L.S., Huang, L., Gécz, J., and Wilkinson, M.F. (2009). A UPF3-mediated regulatory switch that maintains RNA surveillance. Nat. Struct. Mol. Biol. *16*, 747–753.

Chazal, P.E., Daguenet, E., Wendling, C., Ulryck, N., Tomasetto, C., Sargueil, B., and Le Hir, H. (2013). EJC core component MLN51 interacts with eIF3 and activates translation. Proc. Natl. Acad. Sci. USA *110*, 5903–5908.

Danckwardt, S., Gantzert, A.S., Macher-Goeppinger, S., Probst, H.C., Gentzel, M., Wilm, M., Gröne, H.J., Schirmacher, P., Hentze, M.W., and Kulozik, A.E. (2011). p38 MAPK controls prothrombin expression by regulated RNA 3′ end processing. Mol. Cell *41*, 298–310.

Degot, S., Le Hir, H., Alpy, F., Kedinger, V., Stoll, I., Wendling, C., Seraphin, B., Rio, M.C., and Tomasetto, C. (2004). Association of the breast cancer protein MLN51 with the exon junction complex via its speckle localizer and RNA binding module. J. Biol. Chem. *279*, 33702–33715.

Dobin, A., Davis, C.A., Schlesinger, F., Drenkow, J., Zaleski, C., Jha, S., Batut, P., Chaisson, M., and Gingeras, T.R. (2013). STAR: ultrafast universal RNA-seq aligner. Bioinformatics *29*, 15–21.

Friedersdorf, M.B., and Keene, J.D. (2014). Advancing the functional utility of PAR-CLIP by quantifying background binding to mRNAs and lncRNAs. Genome Biol. *15*, R2.

Gehring, N.H., Kunz, J.B., Neu-Yilik, G., Breit, S., Viegas, M.H., Hentze, M.W., and Kulozik, A.E. (2005). Exon-junction complex components specify distinct routes of nonsense-mediated mRNA decay with differential cofactor requirements. Mol. Cell *20*, 65–75.

Gehring, N.H., Lamprinaki, S., Hentze, M.W., and Kulozik, A.E. (2009a). The hierarchy of exon-junction complex assembly by the spliceosome explains key features of mammalian nonsense-mediated mRNA decay. PLoS Biol. *7*, e1000120.

Gehring, N.H., Lamprinaki, S., Kulozik, A.E., and Hentze, M.W. (2009b). Disassembly of exon junction complexes by PYM. Cell *137*, 536–548.

Hachet, O., and Ephrussi, A. (2004). Splicing of oskar RNA in the nucleus is coupled to its cytoplasmic localization. Nature *428*, 959–963.

Han, A., Stoilov, P., Linares, A.J., Zhou, Y., Fu, X.D., and Black, D.L. (2014). De novo prediction of PTBP1 binding and splicing targets reveals unexpected features of its RNA recognition and function. PLoS Comput. Biol. *10*, e1003442.

Hauer, C., Curk, T., Anders, S., Schwarzl, T., Alleaume, A.M., Sieber, J., Hollerer, I., Bhuvanagiri, M., Huber, W., Hentze, M.W., and Kulozik, A.E. (2015). Improved binding site assignment by high-resolution mapping of RNA-protein interactions using iCLIP. Nat. Commun. *6*, 7921.

Huang, W., Sherman, B.T., and Lempicki, R.A. (2009). Bioinformatics enrichment tools: paths toward the comprehensive functional analysis of large gene lists. Nucleic Acids Res. *37*, 1–13.

Huang, L., Lou, C.H., Chan, W., Shum, E.Y., Shao, A., Stone, E., Karam, R., Song, H.W., and Wilkinson, M.F. (2011). RNA homeostasis governed by cell type-specific and branched feedback loops acting on NMD. Mol. Cell *43*, 950–961.

Huppertz, I., Attig, J., D'Ambrogio, A., Easton, L.E., Sibley, C.R., Sugimoto, Y., Tajnik, M., König, J., and Ule, J. (2014). iCLIP: protein-RNA interactions at nucleotide resolution. Methods *65*, 274–287.

Kim, V.N., Kataoka, N., and Dreyfuss, G. (2001). Role of the nonsense-mediated decay factor hUpf3 in the splicing-dependent exon-exon junction complex. Science *293*, 1832–1836.

König, J., Zarnack, K., Rot, G., Curk, T., Kayikci, M., Zupan, B., Turner, D.J., Luscombe, N.M., and Ule, J. (2010). iCLIP reveals the function of hnRNP particles in splicing at individual nucleotide resolution. Nat. Struct. Mol. Biol. *17*, 909–915.

Le Hir, H., Izaurralde, E., Maquat, L.E., and Moore, M.J. (2000). The spliceosome deposits multiple proteins 20-24 nucleotides upstream of mRNA exon-exon junctions. EMBO J. *19*, 6860–6869.

Le Hir, H., Gatfield, D., Izaurralde, E., and Moore, M.J. (2001). The exon-exon junction complex provides a binding platform for factors involved in mRNA export and nonsense-mediated mRNA decay. EMBO J. *20*, 4987–4997.

Mayeda, A., Badolato, J., Kobayashi, R., Zhang, M.Q., Gardiner, E.M., and Krainer, A.R. (1999). Purification and characterization of human RNPS1: a general activator of pre-mRNA splicing. EMBO J. *18*, 4560–4570.

McGlincy, N.J., and Smith, C.W. (2008). Alternative splicing resulting in nonsense-mediated mRNA decay: what is the meaning of nonsense? Trends Biochem. Sci. *33*, 385–393.

Melero, R., Buchwald, G., Castaño, R., Raabe, M., Gil, D., Lázaro, M., Urlaub, H., Conti, E., and Llorca, O. (2012). The cryo-EM structure of the UPF-EJC complex shows UPF1 poised toward the RNA 3′ end. Nat. Struct. Mol. Biol. *19*, 498–505, S1–S2.

Mendell, J.T., Sharifi, N.A., Meyers, J.L., Martinez-Murillo, F., and Dietz, H.C. (2004). Nonsense surveillance regulates expression of diverse classes of mammalian transcripts and mutes genomic noise. Nat. Genet. *36*, 1073–1078.

Meyuhas, O. (2000). Synthesis of the translational apparatus is regulated at the translational level. Eur. J. Biochem. *267*, 6321–6330.

Michelle, L., Cloutier, A., Toutant, J., Shkreta, L., Thibault, P., Durand, M., Garneau, D., Gendron, D., Lapointe, E., Couture, S., et al. (2012). Proteins associated with the exon junction complex also control the alternative splicing of apoptotic regulators. Mol. Cell. Biol. *32*, 954–967.

Millevoi, S., Decorsière, A., Loulergue, C., Iacovoni, J., Bernat, S., Antoniou, M., and Vagner, S. (2009). A physical and functional link between splicing factors promotes pre-mRNA 3′ end processing. Nucleic Acids Res. *37*, 4672–4683.

Murachelli, A.G., Ebert, J., Basquin, C., Le Hir, H., and Conti, E. (2012). The structure of the ASAP core complex reveals the existence of a Pinin-containing PSAP complex. Nat. Struct. Mol. Biol. *19*, 378–386.

Nott, A., Le Hir, H., and Moore, M.J. (2004). Splicing enhances translation in mammalian cells: an additional function of the exon junction complex. Genes Dev. *18*, 210–222.

Palacios, I.M., Gatfield, D., St Johnston, D., and Izaurralde, E. (2004). An eIF4AIII-containing complex required for mRNA localization and nonsense-mediated mRNA decay. Nature *427*, 753–757.

Pimentel, H., Parra, M., Gee, S., Ghanem, D., An, X., Li, J., Mohandas, N., Pachter, L., and Conboy, J.G. (2014). A dynamic alternative splicing program regulates gene expression during terminal erythropoiesis. Nucleic Acids Res. *42*, 4031–4042.

Robinson, M.D., McCarthy, D.J., and Smyth, G.K. (2010). edgeR: a Bioconductor package for differential expression analysis of digital gene expression data. Bioinformatics *26*, 139–140.

Robinson, J.T., Thorvaldsdóttir, H., Winckler, W., Guttman, M., Lander, E.S., Getz, G., and Mesirov, J.P. (2011). Integrative genomics viewer. Nat. Biotechnol. *29*, 24–26.

Ruvinsky, I., and Meyuhas, O. (2006). Ribosomal protein S6 phosphorylation: from protein synthesis to cell size. Trends Biochem. Sci. *31*, 342–348.

Saltzman, A.L., Kim, Y.K., Pan, Q., Fagnani, M.M., Maquat, L.E., and Blencowe, B.J. (2008). Regulation of multiple core spliceosomal proteins by alternative splicing-coupled nonsense-mediated mRNA decay. Mol. Cell. Biol. *28*, 4320–4330.

Saulière, J., Murigneux, V., Wang, Z., Marquenet, E., Barbosa, I., Le Tonquèze, O., Audic, Y., Paillard, L., Roest Crollius, H., and Le Hir, H. (2012). CLIP-seq of eIF4AIII reveals transcriptome-wide mapping of the human exon junction complex. Nat. Struct. Mol. Biol. *19*, 1124–1131.

Schwanhäusser, B., Busse, D., Li, N., Dittmar, G., Schuchhardt, J., Wolf, J., Chen, W., and Selbach, M. (2011). Global quantification of mammalian gene expression control. Nature *473*, 337–342.

Shen, L., Shao, N., Liu, X., and Nestler, E. (2014). ngs.plot: Quick mining and visualization of next-generation sequencing data by integrating genomic databases. BMC Genomics *15*, 284.

Shibuya, T., Tange, T.O., Sonenberg, N., and Moore, M.J. (2004). eIF4AIII binds spliced mRNA in the exon junction complex and is essential for nonsense-mediated decay. Nat. Struct. Mol. Biol. *11*, 346–351.

Shum, E.Y., Jones, S.H., Shao, A., Dumdie, J., Krause, M.D., Chan, W.K., Lou, C.H., Espinoza, J.L., Song, H.W., Phan, M.H., et al. (2016). The antagonistic gene paralogs Upf3a and Upf3b govern nonsense-mediated RNA decay. Cell *165*, 382–395.

Sims, D., Sudbery, I., Ilott, N.E., Heger, A., and Ponting, C.P. (2014). Sequencing depth and coverage: key considerations in genomic analyses. Nat. Rev. Genet. *15*, 121–132.

Singh, G., Kucukural, A., Cenik, C., Leszyk, J.D., Shaffer, S.A., Weng, Z., and Moore, M.J. (2012). The cellular EJC interactome reveals higher-order mRNP structure and an EJC-SR protein nexus. Cell *151*, 750–764.

Spellman, R., and Smith, C.W. (2006). Novel modes of splicing repression by PTB. Trends Biochem. Sci. *31*, 73–76.

Sugimoto, Y., König, J., Hussain, S., Zupan, B., Curk, T., Frye, M., and Ule, J. (2012). Analysis of CLIP and iCLIP methods for nucleotide-resolution studies of protein-RNA interactions. Genome Biol. *13*, R67.

Sureau, A., Gattoni, R., Dooghe, Y., Stévenin, J., and Soret, J. (2001). SC35 autoregulates its expression by promoting splicing events that destabilize its mRNAs. EMBO J. *20*, 1785–1796.

Tange, T.O., Shibuya, T., Jurica, M.S., and Moore, M.J. (2005). Biochemical analysis of the EJC reveals two new factors and a stable tetrameric protein core. RNA *11*, 1869–1883.

Tani, H., Imamachi, N., Salam, K.A., Mizutani, R., Ijiri, K., Irie, T., Yada, T., Suzuki, Y., and Akimitsu, N. (2012). Identification of hundreds of novel UPF1 target transcripts by direct determination of whole transcriptome stability. RNA Biol. *9*, 1370–1379.

Thorvaldsdóttir, H., Robinson, J.T., and Mesirov, J.P. (2013). Integrative Genomics Viewer (IGV): high-performance genomics data visualization and exploration. Brief. Bioinform. *14*, 178–192.

Wang, Z., Murigneux, V., and Le Hir, H. (2014). Transcriptome-wide modulation of splicing by the exon junction complex. Genome Biol. *15*, 551.

Wittmann, J., Hol, E.M., and Jäck, H.M. (2006). hUPF2 silencing identifies physiologic substrates of mammalian nonsense-mediated mRNA decay. Mol. Cell. Biol. *26*, 1272–1287.

Xue, Y., Zhou, Y., Wu, T., Zhu, T., Ji, X., Kwon, Y.S., Zhang, C., Yeo, G., Black, D.L., Sun, H., et al. (2009). Genome-wide analysis of PTB-RNA interactions reveals a strategy used by the general splicing repressor to modulate exon inclusion or skipping. Mol. Cell *36*, 996–1006.

Yeo, G.W., Coufal, N.G., Liang, T.Y., Peng, G.E., Fu, X.D., and Gage, F.H. (2009). An RNA code for the FOX2 splicing regulator revealed by mapping RNA-protein interactions in stem cells. Nat. Struct. Mol. Biol. *16*, 130–137.

Yepiskoposyan, H., Aeschimann, F., Nilsson, D., Okoniewski, M., and Mühlemann, O. (2011). Autoregulation of the nonsense-mediated mRNA decay pathway in human cells. RNA *17*, 2108–2118.

Cell Reports, Volume *16*

Supplemental Information

Exon Junction Complexes Show a Distributional Bias toward Alternatively Spliced mRNAs and against mRNAs Coding for Ribosomal Proteins

Christian Hauer, Jana Sieber, Thomas Schwarzl, Ina Hollerer, Tomaz Curk, Anne-Marie Alleaume, Matthias W. Hentze, and Andreas E. Kulozik

Supplemental Figures

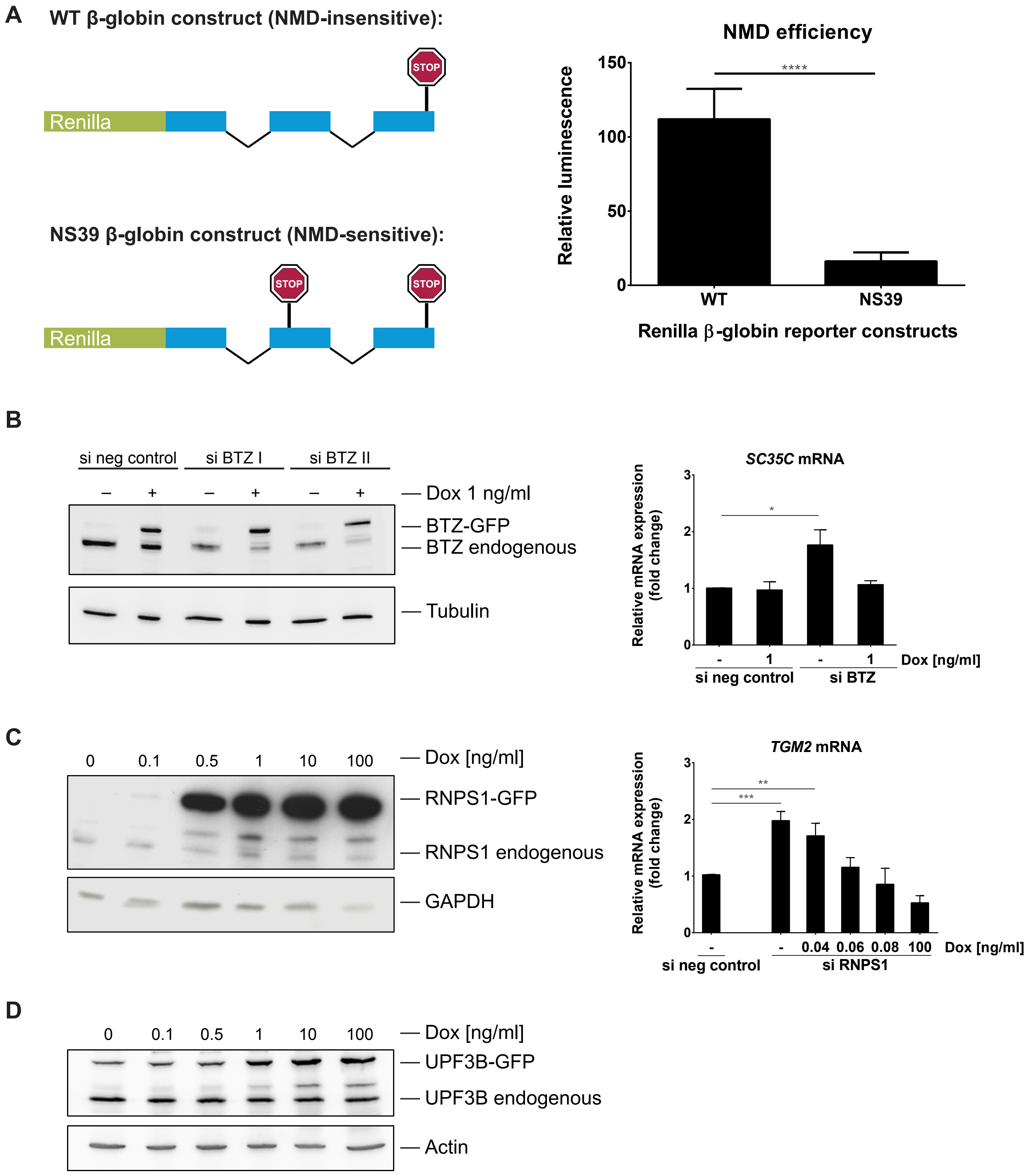

Figure S1. Related to Figure 1: Validation of HeLa cell lines. (a) Reporter constructs to measure the NMD efficiency of HeLa host cell line. The nonsense-mediated mRNA decay (NMD) pathway is fully functional in the HeLa cells used for iCLIP analyses. Error bars represent standard deviation and ****p-value < 0.0001 was calculated by two-tailed Student´s t-test, n = 3. (b-d) Validation of Hela cell lines with stably integrated fusion genes coding for proteins of (b) BTZGFP, (c) RNPS1-GFP, (d) UPF3B-GFP. The siRNA rescue experiments were only conducted for eIF4A3, BTZ and RNPS1. Error bars represent standard error of the mean and p-values were calculated by one-way ANOVA with Dunnett´s multiple comparison test; *p-value<0.05, **p-value<0.01, ***p-value<0.001 with n=3–5 independent biological experiments.Dox=doxycycline.

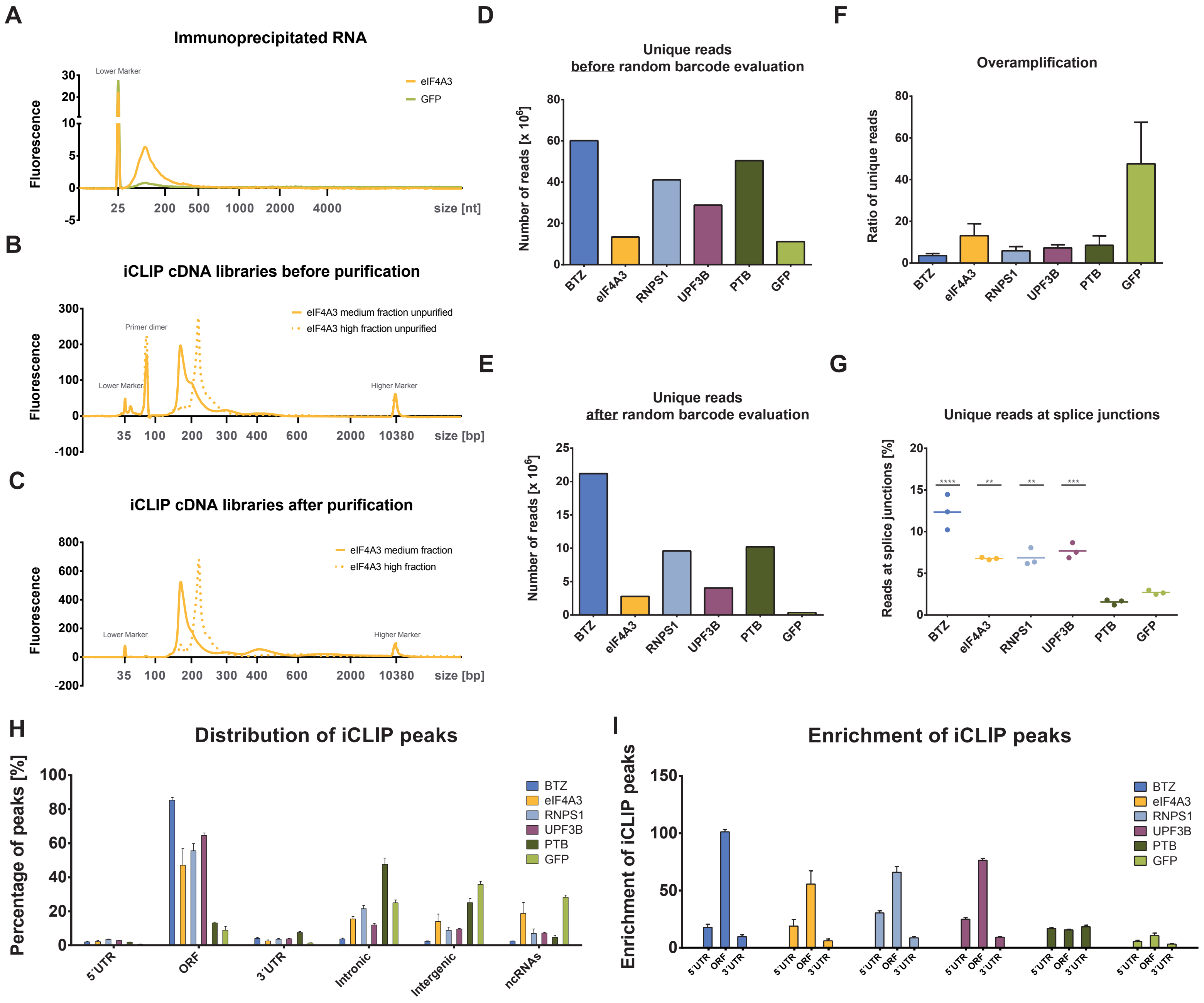

Figure S2. Related to Figure 2: Quality control of iCLIP libraries. (a-c) Size distributions during iCLIP. (a) Size distribution of co-immunoprecipitated RNA fragments after pull-down of eIF4A3-GFP and, as a control, of GFP. (b) After reverse transcription a cDNA size range between 150–300 bp was achieved and (c) primer dimers were successfully removed prior to sequencing. (d-f) iCLIP libraries of RPBs show low overamplification rates. The number of uniquely mapped reads was calculated as the sum of three biologically independent experiments before (d) and (e) after random barcode evaluation. (f) The overamplification rate was determined by the ratio of the uniquely mapped reads before and after random barcode evaluation for each replicate. The low number of uniquely mapped reads after random barcode evaluation and the high overamplification rate for GFP libraries indicated a low complexity of these libraries and validated GFP as an appropriate background control. (e) The four EJC proteins, but not PTB, were significantly enriched at the exon-exon boundaries in comparison to the GFP background control. Horizontal lines represent the mean of n = 3 independent biological experiments and the p-values compared to GFP were calculated by one-way ANOVA with Dunnett´s multiple comparison test; **p-value < 0.01, ***p-value < 0.001, ****p-value < 0.0001.
h) Distribution of peaksof all iCLIP datasets in % confirmed that EJC components bind predominantly in the ORF. This graph does not reflect the different library sizes and thus, in particular, the low GFP counts have to be taken into account. The sum of GFP reads after random barcode evaluation from the three replicates was 10-100 times lower compared to EJC and PTB reads (see panel E and Table S1). (f) Enrichment of iCLIP peaks as in Figure 2 with GFP included. Error bars represent the standard error of the mean.

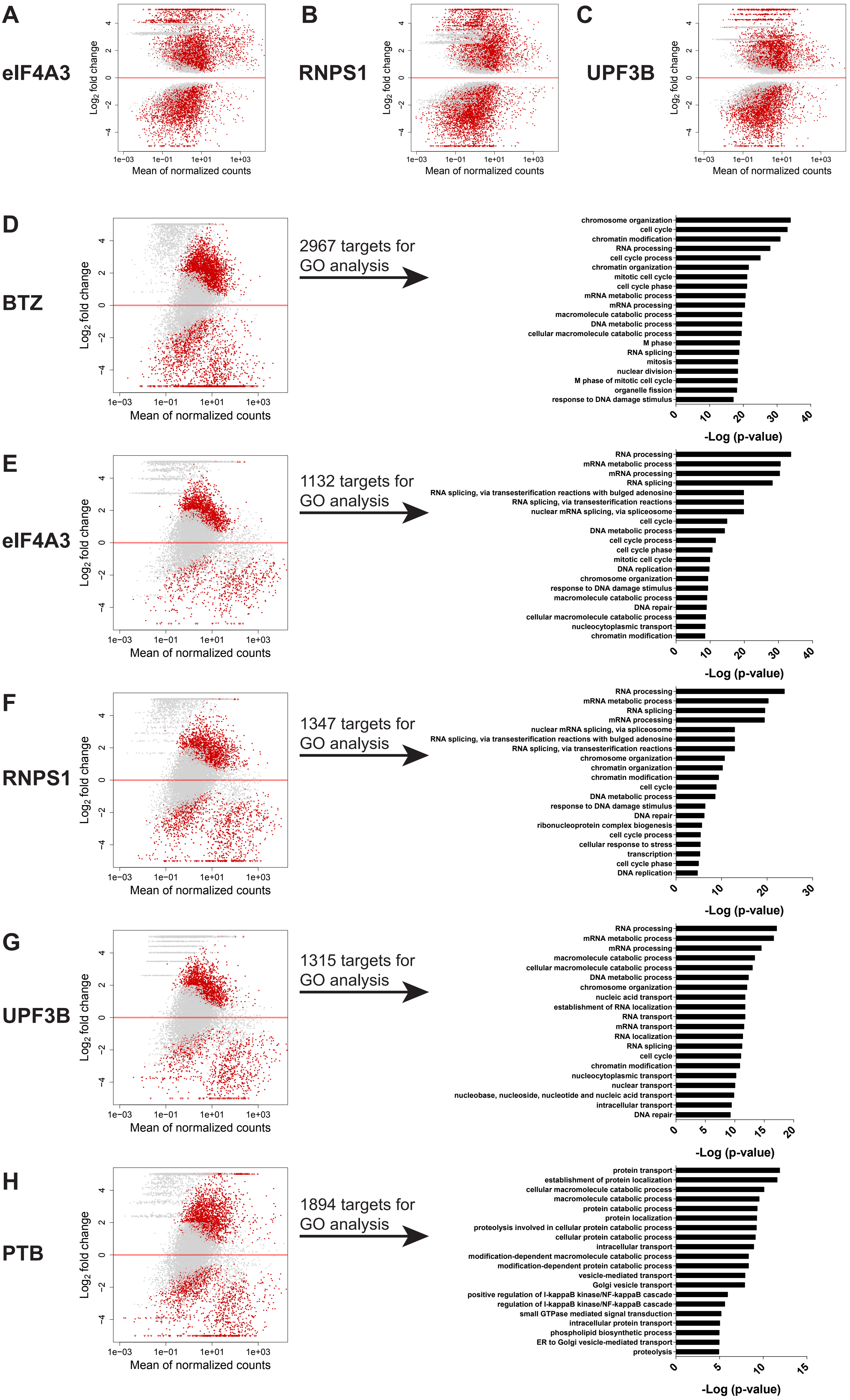

A
eIF4A3
B
RNPS1
C
UPF3B
Log2 fold change
Mean of normalized counts
1e−03
1e−01
1e+01
1e+03
D
BTZ
2967 targets for GO analysis
chromosome organization
cell cycle
chromatin modification
RNA processing
cell cycle process
chromatin organization
mitotic cell cycle
cell cycle phase
mRNA metabolic process
mRNA processing
macromolecule catabolic process
DNA metabolic process
cellular macromolecule catabolic process
M phase
RNA splicing
mitosis
nuclear division
M phase of mitotic cell cycle
organelle fission
response to DNA damage stimulus
-Log (p-value)
E
eIF4A3
1132 targets for GO analysis
RNA processing
mRNA metabolic process
mRNA processing
RNA splicing
RNA splicing, via transesterification reactions with bulged adenosine
RNA splicing, via transesterification reactions
nuclear mRNA splicing, via spliceosome
cell cycle
DNA metabolic process
cell cycle process
cell cycle phase
mitotic cell cycle
DNA replication
chromosome organization
response to DNA damage stimulus
macromolecule catabolic process
DNA repair
cellular macromolecule catabolic process
nucleocytoplasmic transport
chromatin modification
-Log (p-value)
F
RNPS1
1347 targets for GO analysis
RNA processing
mRNA metabolic process
RNA splicing
mRNA processing
nuclear mRNA splicing, via spliceosome
RNA splicing, via transesterification reactions with bulged adenosine
RNA splicing, via transesterification reactions
chromosome organization
chromatin organization
chromatin modification
cell cycle
DNA metabolic process
response to DNA damage stimulus
DNA repair
ribonucleoprotein complex biogenesis
cell cycle process
cellular response to stress
transcription
cell cycle phase
DNA replication
-Log (p-value)
G
UPF3B
1315 targets for GO analysis
RNA processing
mRNA metabolic process
mRNA processing
macromolecule catabolic process
cellular macromolecule catabolic process
DNA metabolic process
chromosome organization
nucleic acid transport
establishment of RNA localization
RNA transport
mRNA transport
RNA localization
RNA splicing
cell cycle
chromatin modification
nucleocytoplasmic transport
nuclear transport
nucleobase, nucleoside, nucleotide and nucleic acid transport
intracellular transport
DNA repair
-Log (p-value)
H
PTB
1894 targets for GO analysis
protein transport
establishment of protein localization
cellular macromolecule catabolic process
macromolecule catabolic process
protein catabolic process
protein localization
proteolysis involved in cellular protein catabolic process
cellular protein catabolic process
intracellular transport
modification-dependent macromolecule catabolic process
modification-dependent protein catabolic process
vesicle-mediated transport
Golgi vesicle transport
positive regulation of I-kappaB kinase/NF-kappaB cascade
regulation of I-kappaB kinase/NF-kappaB cascade
small GTPase mediated signal transduction
intracellular protein transport
phospholipid biosynthetic process
ER to Golgi vesicle-mediated transport
proteolysis
-Log (p-value)

Figure S3. Related to Figure 3: Differential analysis of EJC data compared to PTB and GFP iCLIP data. The red dots in the plots show signigicant mRNA targets either up- or downregulated after differential analysis using edgeR controlled by Benjamini-Hochberg procedure with a FDR < 0.05. (a-c) Analog to Figure 4A for (a) eIF4A3, (b) RNPS1 and (c) UPF3B. (d-h) Targets that are above the red line have a higher signal for the EJC (or PTB) component compared to GFP. These targets are analyzed for gene ontology (GO) enrichment for (d) BTZ, (e) eIF4A3, (f) RNPS1, (g) UPF3B and (h) PTB.

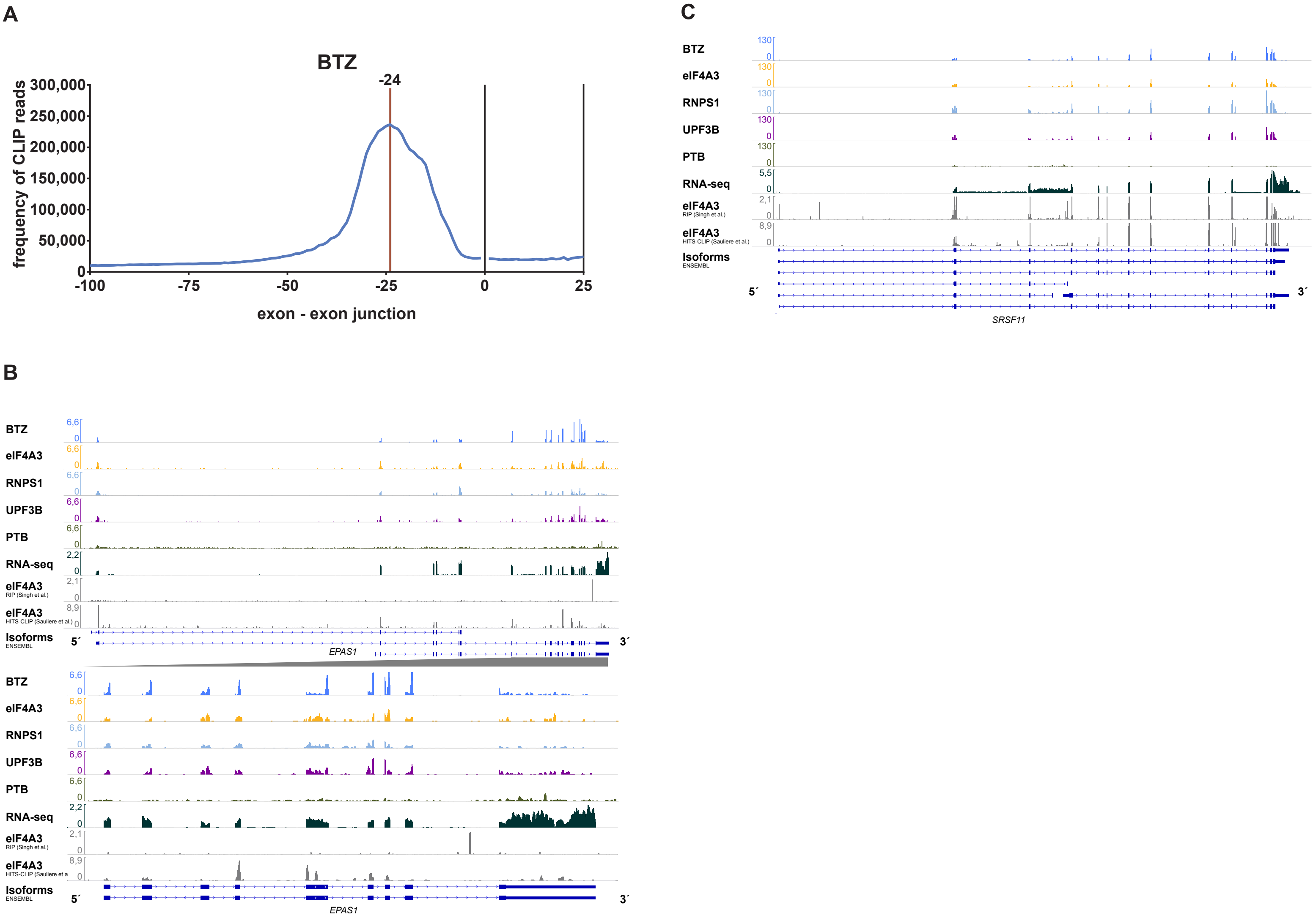

Figure S4. Related to Figures 4, 5, and 6: (a) Metablot of iCLIP reads of one BTZ replictate at exon-exon junctions. The vast majority of the signal clusters around the -24 nts position (red bar). (b-c) Genome browser view of mRNA targets belonging to alternative NMD pathways indicates homogenous EJC composition. (b) *EPAS1* mRNA is RNPS1 sensitive (Gehring et al., 2015), whereas (c) *SRSF11* mRNA is UPF3B sensitive (Chan et al., 2007). The track range displays count per million (CPM) and was adjusted to the highest iCLIP signal obtained in each genome browser view. The signals of the RNA-Seq and literature data were not adjusted. Literature data were obtained from RIP (Singh et al., 2012) and HITS-CLIP (Sauliere et al., 2012) of eIF4A3.

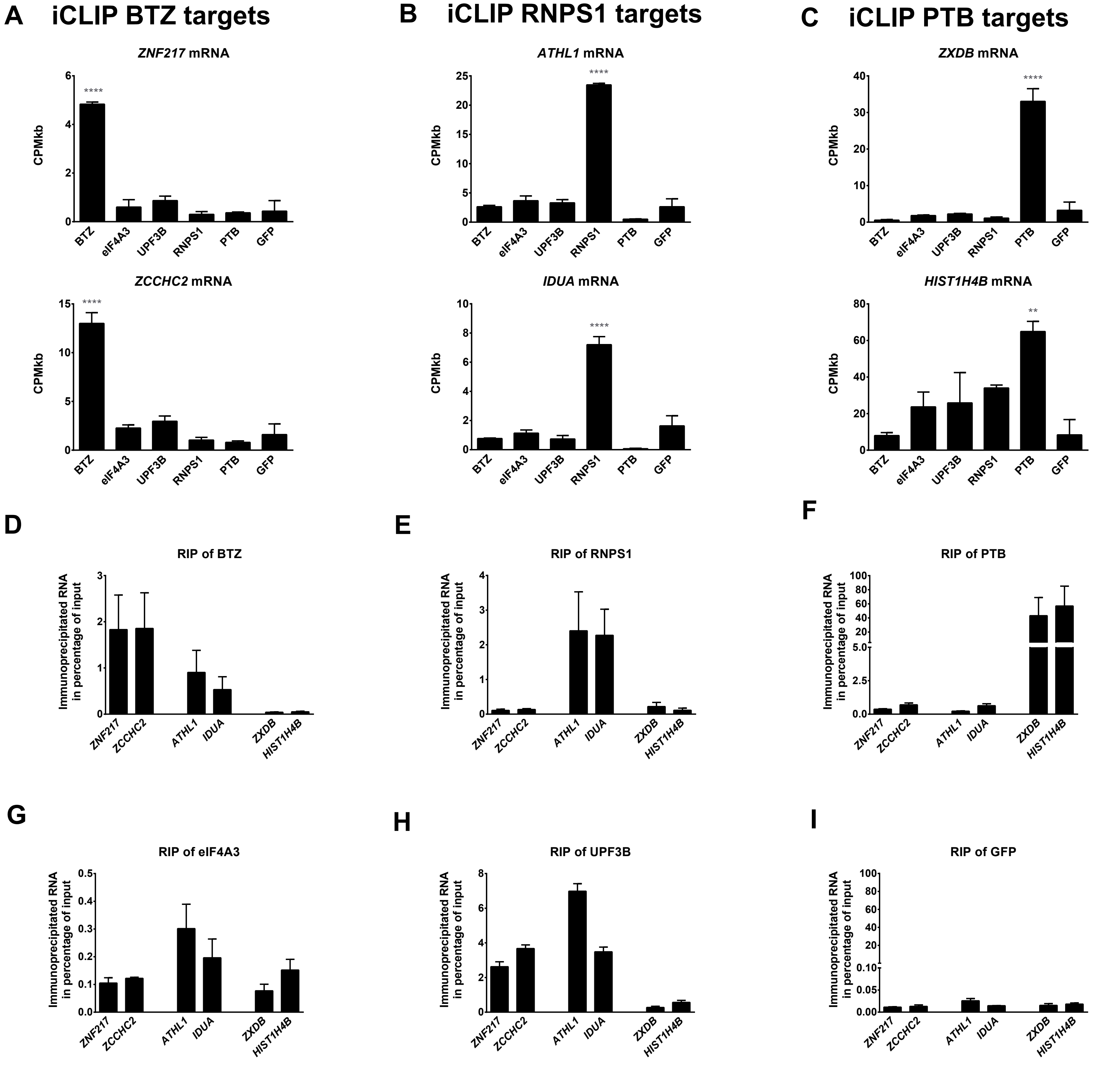

Figure S5 related to Figure 4: Selected mRNAs that were enriched for (a) BTZ, (b) RNPS1 and (c) PTB binding sites in iCLIP analysis. p-values compared to GFP were calculated by one-way ANOVA with Dunnett´s multiple comparison test; **p-value < 0.01, ****p-value <0.0001 with n = 3 independent biological experiments. CPMkb: counts per million mapped reads adjusted by gene length in kilobases. iCLIP identified targets of BTZ (*ZNF217*, *ZCCHC2*) and RNPS1 (*ATHL1*, *IDUA*) were quantified together with intronless transcripts enriched for PTB in the 3´UTR (*ZXDB*, *HIST1H4B*) using RNA immunoprecipitation (RIP) experiments (d-i). The RIP experiments of (d) BTZ, (e) RNPS1 and (f) PTB indicated distince specificities.
The RIP experiments of (g) eIF4A3 and (h) UPF3B exhibiteda similar pattern, but strong differences in the amount of co-immunoprecipitated RNA. The control immunoprecipitation of (i) GFP only resulted in background signals for all transcripts. Error bars represent standard error of the mean of n = 3 independentbiological experiments.

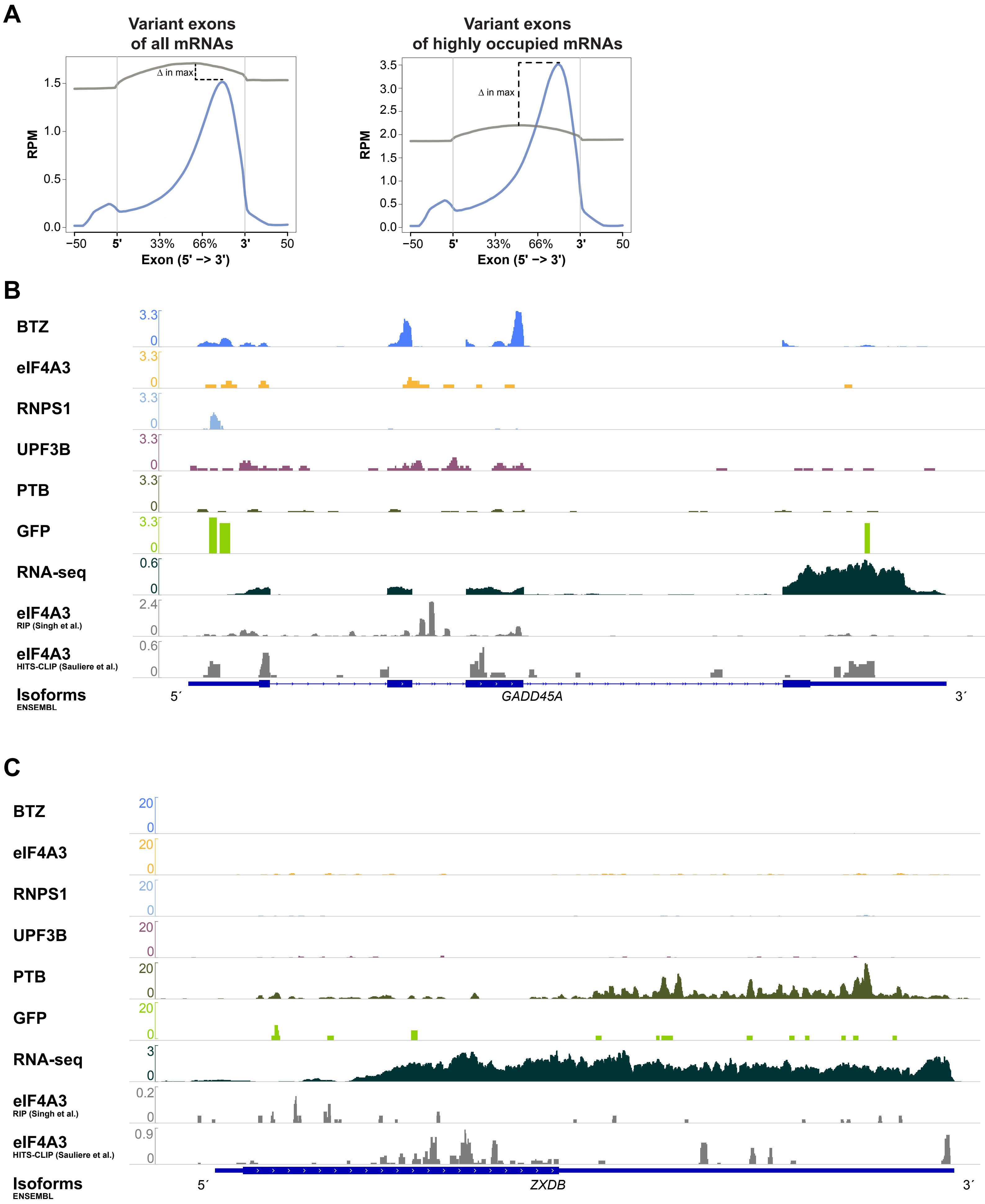

Figure S6. Related to Figure 5: Analysis of binding sites at specific exons and transcripts. (a) Scheme of how the data depicted in Figure 5A is generated. (b) Genome browser view of a low-abundance transcript, *GADD45A*, demonstrates the sensitive detection of EJC deposition sites. (c) Genome browser view on an intronless transcript, *ZXDB*, demonstrates the high degree of specificity of the iCLIP data, because only PTB and none of the EJC components were bound to the transcript. In particular, most of the PTB signal was located in the 3´UTR of this transcript. The track range displays count per million (CPM) and was adjusted to the highest iCLIP signal obtained in each genome browser view. The signals of the RNA-Seq and literature data were not adjusted. Literature data were obtained from RIP (Singh et al., 2012) and HITS-CLIP (Sauliere et al., 2012) of eIF4A3.

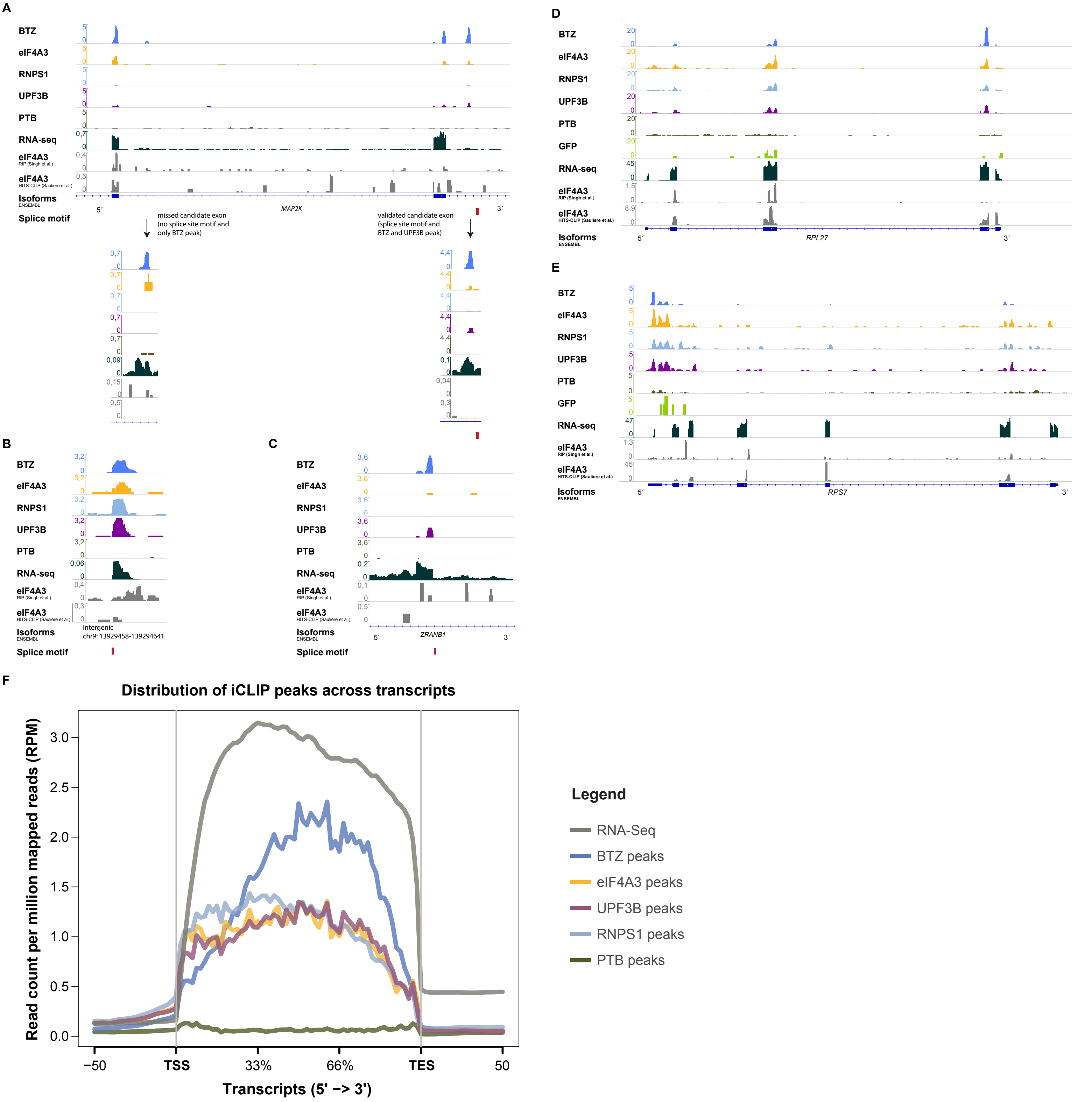

Figure S7. Related to Figure 4, 5 and 6: (a-c) Genome browser view of candidate exons. (a) *MAP2K4* mRNA with 4 BTZ peaks. Only two peaks overlap with annotated exons. The other two peaks map to intronic regions that are enriched in the RNA-seq compared to the surrounding (see zoom in). Our approach only detects the downstream region as a candidate exon due to the peak height and the splice site donor motif in the correct position. Further candidate exons were found in intergenic (b) and (c) intronic regions in the transcriptome (see also Table S5).

(d-e) Genome browser view of mRNAs coding for ribosomal proteins indicates differential binding of EJC components. (d) Internal exons of the mRNA coding for RPL27 possessed an EJC signal, whereas (b) internal exons of the mRNA coding for RBS7 did not exhibit a signal for EJC deposition.

The track range displays count per million (CPM) and was adjusted to the highest iCLIP signal obtained in each genome browser view. The signals of the RNA-Seq and literature data were not adjusted. Literature data were obtained from RIP (Singh et al., 2012) and HITS-CLIP (Sauliere et al., 2012) of eIF4A3.

(f) The RNA-seq and the EJC iCLIP datasets cover the entire gene body and thus EJC binding is not biased neither towards the 5´ nor the 3´ end of transcripts. Average profile of reads covering protein-coding mRNAs from the transcription start site (TSS) until the transcription end site (TES) are plotted as read count per million mapped reads (RPM) using ngsplot software (Shen et al., 2014).

Supplemental Tables

Table S1: Statistics for iCLIP libraries. Deletion and mismatch rates were calculated per base. Last column represents the final quality-controlled reads. Related to Figure 2.

	Rep.	Adapter removal		Mapped reads						Barcode
		Input reads	Output reads	Unique	Unique ratio	Mismatches	Deletions	Multiple loci	Too many loci	Evaluated reads
eIF4A3	1	17,583,508	16,523,573	7,143,889	43.23%	0.37%	0.08%	39.68%	14.70%	300,471
	2	29,781,052	28,329,506	9,190,552	32.44%	0.45%	0.07%	50.27%	7.22%	2,230,337
	3	9,832,432	9,349,933	2,950,133	31.55%	0.57%	0.04%	43.84%	16.51%	256,743
BTZ	1	35,130,380	33,715,899	19,720,084	58.49%	0.43%	0.04%	35.94%	2.49%	3,871,095
	2	39,043,789	37,127,273	23,722,280	63.89%	0.30%	0.05%	27.85%	2.55%	12,907,006
	3	30,111,493	28,639,999	16,662,127	58.18%	0.42%	0.03%	29.17%	5.26%	4,404,326
RNPS1	1	36,757,266	34,628,259	19,155,698	55.32%	0.50%	0.06%	33.15%	8.44%	3,582,604
	2	36,708,404	34,475,502	14,520,941	42.12%	0.45%	0.08%	36.83%	9.33%	5,246,574
	3	20,049,932	19,156,642	7,415,654	38.71%	0.60%	0.04%	32.87%	14.91%	776,597
UPF3B	1	31,038,246	29,478,768	11,898,252	40.36%	0.54%	0.06%	50.09%	6.35%	1,247,946
	2	28,272,349	26,188,344	10,547,640	40.28%	0.53%	0.07%	49.91%	6.26%	1,350,142
	3	24,446,683	23,362,640	6,414,348	27.46%	0.56%	0.06%	53.35%	4.79%	1,455,514
PTB	1	23,501,063	22,691,882	13,302,382	58.62%	0.47%	0.11%	30.33%	3.78%	6,933,311
	2	19,356,590	18,688,782	11,147,430	59.65%	0.51%	0.05%	30.25%	3.94%	1,777,489
	3	50,139,813	47,293,611	25,973,284	54.92%	0.33%	0.04%	32.87%	4.73%	1,501,573
GFP	1	21,484,620	17,976,060	3,489,070	19.41%	0.54%	0.08%	63.14%	8.23%	43,702
	2	9,925,921	9,539,105	2,389,971	25.05%	0.68%	0.04%	44.93%	15.59%	211,148
	3	25,181,318	23,847,567	5,280,032	22.14%	0.73%	0.03%	42.94%	8.09%	102,122

Table S2: The 20 most enriched 5mers for the iCLIP datasets performed in triplicate in descending order. The colored 5mers contain a GT-motif. Related to Figure 2.

	eIF4A3		BTZ		RNPS1		UPF3B		PTB		GFP	
	5mer	z-score	5mer	z-score	5mer	z-score	5mer	z-score	5mer	z-score	5mer	z-score
1	AGGTA	200	AGGTA	908	GAAGA	322	GAAGA	189	TCTTC	401	GGGGG	103
2	GGTAA	151	GGTAA	803	AGAAG	268	AGGTA	166	CTTCC	380	CCCCC	87
3	GTAAG	144	GTAAG	713	AAGAA	250	GGTAA	165	CTTCT	373	AAAAA	74
4	GTGAG	143	GTGAG	526	GAGGA	234	AGAAG	154	TCTCT	360	TTTTT	56
5	GGTGA	136	CAGGT	510	AGGAG	209	AAGAA	139	CTCTT	360	GGCGG	44
6	CGGCG	122	AAGGT	486	GCGGC	204	GTAAG	137	TCTCC	358	CGGCG	40
7	CAGGT	122	GGTGA	455	GGCGG	183	GTGAG	122	TCCTC	357	GCGGG	40
8	GCGGC	121	GAAGA	354	CGGCG	182	GGTGA	115	CTCTC	350	GCGGC	36
9	GCCCC	108	AGGTG	331	CCCCC	177	GAGGA	105	TCCCT	348	CGGGG	35
10	CCGGC	108	TAAGT	312	GGAGG	175	AAGGT	100	CTTTC	346	GGGCG	30
11	GGCGG	107	AGAAG	292	AGGTA	171	AGGTG	93	CCTTC	345	CAGGG	28
12	CCCGG	106	AAGAA	279	TCCCC	164	GCGGC	93	TTCTC	343	CTCTC	27
13	CGGCC	106	TGAGT	254	CCCCG	164	CAGGT	89	CCTCT	339	CCCCG	27
14	CCCCG	103	GGTAT	243	GCCCC	156	GGCGG	88	TTCCC	326	TCTCT	26
15	CCCCC	100	GAGGT	233	CCCGG	154	CGGCG	83	TCCTT	325	GGTCG	26
16	AAGGT	98	GTATG	212	AGCGG	152	GGAGG	74	TCCCC	323	TGATC	24
17	AGGTG	92	CAAGG	187	CCCTC	151	AGGAG	72	TTTCC	310	GGGGC	22
18	CGCCG	88	GGTAG	186	CCTCC	151	AAAAA	71	TTCCT	303	GGGAG	22
19	GCCGG	86	GGTAC	179	GGTAA	148	CCCCG	71	CCTTT	294	CCCTC	22
20	GCTGC	79	AAGTA	177	AGAGG	141	GCCCC	70	TTTCT	291	CGCCC	21

Table S3: Number of peaks in iCLIP libraries. Related to Figure 2.

	BTZ	eIF4A3	RNPS1	UPF3B	PTB	Overlap
**Total Peaks**	173,944	48,584	127,642	68,146	172,175	
**after GFP filtering**	164,627	35,150	115,223	60,374	145,797	
**restricted to one RBP**	89,046	2,613	44,646	6,963	116,969	
**with PTB**						346,396
**w/o PTB, at least one EJC protein**						200,599
**w/o PTB, at least two EJC proteins**						59,521
**w/o PTB, at least two EJC proteins, protein-coding**						54,645
**BTZ + eIF4A3**						2,007
**BTZ + RNPS1**						16,690
**BTZ + UPF3B**						8,694
**eIF4A3 + RNPS1**						1,538
**eIF4A3 + UPF3B**						251
**RNPS1 + UPF3B**						3,026
**BTZ + eIF4A3 + RNPS1**						3,083
**BTZ + eIF4A3 + UPF3B**						1,644
**BTZ + RNPS1 + UPF3B**						10,699
**eIF4A3 + RNPS1 + UPF3B**						894
**BTZ + eIF4A3 + RNPS1 + UPF3B**						10,995
**high-confidence peaks with BTZ**						53,812
**high-confidence peaks w/o BTZ**						5,709

Table S4: Maximal read count per million mapped reads (RPM) value of iCLIP and RNA-seq libraries (Max) including standard error of the mean (SEM). These values correspond to average profiles of different exon types depicted in Figure 4.

	First		Terminal		Canonical		Variant		ALT acceptor		ALT donor		ALT both	
	Max	SEM	Max	SEM	Max	SEM	Max	SEM	Max	SEM	Max	SEM	Max	SEM
**RNA-seq**	1.43	0.02	2.50	0.03	1.41	0.01	1.71	0.03	2.42	0.07	2.45	0.11	4.29	0.23
**BTZ peaks**	0.93	0.01	0.61	0.01	2.51	0.02	1.52	0.03	3.13	0.07	2.35	0.06	3.07	0.15
**eIF4A3 peaks**	1.05	0.02	0.47	0.01	0.92	0.02	0.86	0.03	1.94	0.06	1.57	0.06	3.03	0.16
**UPF3B peaks**	0.79	0.01	0.41	0.01	1.07	0.02	0.75	0.02	1.63	0.06	1.40	0.06	2.29	0.17
**RNPS1 peaks**	0.80	0.01	0.50	0.01	0.64	0.01	0.57	0.01	1.29	0.04	1.20	0.04	2.42	0.11
**PTB peaks**	0.24	0.01	0.16	0.01	0.07	0.00	0.13	0.01	0.14	0.01	0.13	0.01	0.26	0.03

Table S5: EJC deposition on exons of targets belonging to alternative NMD pathways. Related to Figure 4.

chromosome	start	end	gene_ID	gene_name	transcript_ID	exon_category	start_peaks	end_peak	peak_constitution	reference of depletion	siRNA
chr17	66873660	66873820	ENSG00000141338	ABCA8	ENST00000269080	altAcceptor	66873666	66873698	BU	Chan et al., 2007	UPF3B
chr17	66878004	66878121	ENSG00000141338	ABCA8	ENST00000269080	constitutive	66878016	66878042	BU	Chan et al., 2007	UPF3B
chr2	85991114	85991305	ENSG00000168874	ATOH8	ENST00000469442	constitutive	85991243	85991304	BER	Chan et al., 2009	UPF3A and UPF3B
chr7	43843275	43843446	ENSG00000106605	BLVRA	ENST00000265523	constitutive	43843408	43843437	BU	Chan et al., 2007	UPF3B
chr6	87795968	87796152	ENSG00000135346	CGA	ENST00000369582	constitutive	87795929	87796009	BE	Chan et al., 2007	UPF3B
chr2	46609114	46609228	ENSG00000116016	EPAS1	ENST00000263734	constitutive	46609172	46609222	BER	Gehring et al., 2005	RNPS1
chr2	46574012	46574202	ENSG00000116016	EPAS1	ENST00000263734	constitutive	46574168	46574209	BR	Gehring et al., 2005	RNPS1
chr2	46587777	46587895	ENSG00000116016	EPAS1	ENST00000263734	constitutive	46587860	46587895	BR	Gehring et al., 2005	RNPS1
chr2	46602829	46602976	ENSG00000116016	EPAS1	ENST00000263734	constitutive	46602923	46602970	BR	Gehring et al., 2005	RNPS1
chr2	46603678	46603892	ENSG00000116016	EPAS1	ENST00000263734	constitutive	46603827	46603884	BR	Gehring et al., 2005	RNPS1
chr2	46605033	46605226	ENSG00000116016	EPAS1	ENST00000263734	constitutive	46605077	46605106	BR	Gehring et al., 2005	RNPS1
chr2	46605796	46605906	ENSG00000116016	EPAS1	ENST00000263734	constitutive	46605860	46605900	BR	Gehring et al., 2005	RNPS1
chr2	46608735	46608861	ENSG00000116016	EPAS1	ENST00000263734	constitutive	46608725	46608781	BR	Gehring et al., 2005	RNPS1
chr2	46605033	46605226	ENSG00000116016	EPAS1	ENST00000263734	constitutive	46605163	46605218	BU	Gehring et al., 2005	RNPS1
chr2	46609564	46609737	ENSG00000116016	EPAS1	ENST00000263734	constitutive	46609676	46609737	BU	Gehring et al., 2005	RNPS1
chr2	46607366	46607856	ENSG00000116016	EPAS1	ENST00000263734	constitutive	46607800	46607855	BUR	Gehring et al., 2005	RNPS1
chr2	46608735	46608861	ENSG00000116016	EPAS1	ENST00000263734	constitutive	46608823	46608881	BUR	Gehring et al., 2005	RNPS1
chr2	120831676	120831754	ENSG00000115109	EPB41L5	ENST00000263713	constitutive	120831727	120831743	BU	Chan et al., 2009	UPF3A and UPF3B
chr2	120849131	120849237	ENSG00000115109	EPB41L5	ENST00000263713	constitutive	120849193	120849236	BUR	Chan et al., 2009	UPF3A and UPF3B
chrX	133981413	133981505	ENSG00000156500	FAM122C	ENST00000370785	variant	133981467	133981495	BE	Chan et al., 2009	UPF3A and UPF3B
chrX	133963256	133963312	ENSG00000156500	FAM122C	ENST00000370784	constitutive	133963255	133963308	BER	Chan et al., 2009	UPF3A and UPF3B
chrX	133948801	133948879	ENSG00000156500	FAM122C	ENST00000370784	constitutive	133948818	133948879	BEUR	Chan et al., 2009	UPF3A and UPF3B
chrX	133986830	133986926	ENSG00000156500	FAM122C	ENST00000370784	variant	133986889	133986918	BR	Chan et al., 2009	UPF3A and UPF3B
chr9	74828802	74828907	ENSG00000119125	GDA	ENST00000545168	constitutive	74828871	74828899	BER	Chan et al., 2007	UPF3B
chr9	74856068	74856214	ENSG00000119125	GDA	ENST00000545168	constitutive	74856172	74856211	BER	Chan et al., 2007	UPF3B
chr9	74863188	74863322	ENSG00000119125	GDA	ENST00000475764	altDonor	74863289	74863319	BEU	Chan et al., 2007	UPF3B
chr1	158985662	158985777	ENSG00000163565	IFI16	ENST00000368131	constitutive	158985672	158985701	BR	Chan et al., 2009	UPF3A and UPF3B
chr1	159021469	159023514	ENSG00000163565	IFI16	ENST00000493884	altAcceptor	159023368	159023399	BR	Chan et al., 2009	UPF3A and UPF3B
chr15	99442706	99442850	ENSG00000140443	IGF1R	ENST00000268035	constitutive	99442799	99442844	BER	Chan et al., 2007	UPF3B
chr15	99456273	99456511	ENSG00000140443	IGF1R	ENST00000268035	constitutive	99456469	99456510	BEUR	Chan et al., 2007	UPF3B
chr15	99459193	99459360	ENSG00000140443	IGF1R	ENST00000268035	altAcceptor	99459305	99459358	BR	Chan et al., 2007	UPF3B
chr15	99459243	99459360	ENSG00000140443	IGF1R	ENST00000560144	altAcceptor	99459305	99459358	BR	Chan et al., 2007	UPF3B
chr15	99459901	99460105	ENSG00000140443	IGF1R	ENST00000268035	constitutive	99460071	99460097	BR	Chan et al., 2007	UPF3B
chr15	99482430	99482589	ENSG00000140443	IGF1R	ENST00000268035	constitutive	99482548	99482584	BR	Chan et al., 2007	UPF3B
chr15	99486152	99486281	ENSG00000140443	IGF1R	ENST00000268035	constitutive	99486196	99486212	BR	Chan et al., 2007	UPF3B
chr15	99451914	99452128	ENSG00000140443	IGF1R	ENST00000268035	constitutive	99452085	99452127	BU	Chan et al., 2007	UPF3B
chr15	99467105	99467241	ENSG00000140443	IGF1R	ENST00000268035	constitutive	99467208	99467237	BU	Chan et al., 2007	UPF3B
chr15	99486152	99486281	ENSG00000140443	IGF1R	ENST00000268035	constitutive	99486236	99486281	BU	Chan et al., 2007	UPF3B
chr15	99473464	99473534	ENSG00000140443	IGF1R	ENST00000268035	constitutive	99473476	99473569	BUR	Chan et al., 2007	UPF3B
chr15	99478545	99478655	ENSG00000140443	IGF1R	ENST00000268035	constitutive	99478608	99478651	BUR	Chan et al., 2007	UPF3B
chr15	99491803	99491937	ENSG00000140443	IGF1R	ENST00000268035	constitutive	99491802	99491828	BUR	Chan et al., 2007	UPF3B
chr15	99434554	99434866	ENSG00000140443	IGF1R	ENST00000268035	constitutive	99434776	99434790	UR	Chan et al., 2007	UPF3B
chr15	99459901	99460105	ENSG00000140443	IGF1R	ENST00000268035	constitutive	99459957	99459995	UR	Chan et al., 2007	UPF3B
chr15	99465377	99465660	ENSG00000140443	IGF1R	ENST00000268035	constitutive	99465480	99465498	UR	Chan et al., 2007	UPF3B
chr15	99478053	99478282	ENSG00000140443	IGF1R	ENST00000268035	variant	99478102	99478158	UR	Chan et al., 2007	UPF3B
chr4	57898592	57898718	ENSG00000163453	IGFBP7	ENST00000295666	constitutive	57898592	57898629	BUR	Chan et al., 2007	UPF3B
chr2	208030191	208030430	ENSG00000118263	KLF7	ENST00000426163	variant	208030199	208030232	BR	Chan et al., 2007	UPF3B
chr3	122170823	122170911	ENSG00000114030	KPNA1	ENST00000344337	constitutive	122170829	122170861	BEU	Wang et al., 2014	eIF4A3 and BTZ
chr3	122182789	122182888	ENSG00000114030	KPNA1	ENST00000344337	constitutive	122182788	122182843	BEUR	Wang et al., 2014	eIF4A3 and BTZ
chr3	122186169	122186276	ENSG00000114030	KPNA1	ENST00000344337	constitutive	122186169	122186263	BEUR	Wang et al., 2014	eIF4A3 and BTZ
chr3	122215284	122215417	ENSG00000114030	KPNA1	ENST00000344337	constitutive	122215287	122215353	BEUR	Wang et al., 2014	eIF4A3 and BTZ
chr3	122160885	122160963	ENSG00000114030	KPNA1	ENST00000344337	constitutive	122160886	122160940	BR	Wang et al., 2014	eIF4A3 and BTZ
chr3	122168421	122168584	ENSG00000114030	KPNA1	ENST00000344337	constitutive	122168428	122168463	BR	Wang et al., 2014	eIF4A3 and BTZ
chr3	122180071	122180165	ENSG00000114030	KPNA1	ENST00000344337	constitutive	122180081	122180158	BR	Wang et al., 2014	eIF4A3 and BTZ
chr9	126777377	126777804	ENSG00000106689	LHX2	ENST00000446480	altAcceptor	126777763	126777801	BU	Chan et al., 2009	UPF3A and UPF3B
chr9	126777401	126777804	ENSG00000106689	LHX2	ENST00000373615	altAcceptor	126777763	126777801	BU	Chan et al., 2009	UPF3A and UPF3B
chr18	2937690	2938035	ENSG00000101577	LPIN2	ENST00000261596	constitutive	2937703	2937731	BR	Chan et al., 2007	UPF3B
chr18	2926721	2926803	ENSG00000101577	LPIN2	ENST00000261596	constitutive	2926724	2926764	BU	Chan et al., 2007	UPF3B
chr18	2939478	2939601	ENSG00000101577	LPIN2	ENST00000261596	constitutive	2939487	2939515	BU	Chan et al., 2007	UPF3B
chr15	94927251	94927353	ENSG00000140563	MCTP2	ENST00000456504	constitutive	94927306	94927344	BE	Chan et al., 2009	UPF3A and UPF3B
chr15	95019925	95020022	ENSG00000140563	MCTP2	ENST00000456504	constitutive	95019971	95020015	BE	Chan et al., 2009	UPF3A and UPF3B
chr15	94883428	94883504	ENSG00000140563	MCTP2	ENST00000456504	constitutive	94883466	94883498	BER	Chan et al., 2009	UPF3A and UPF3B
chr15	94928652	94928754	ENSG00000140563	MCTP2	ENST00000456504	constitutive	94928708	94928753	BER	Chan et al., 2009	UPF3A and UPF3B
chr15	94983405	94983527	ENSG00000140563	MCTP2	ENST00000456504	variant	94983446	94983527	BEU	Chan et al., 2009	UPF3A and UPF3B
chr15	94841430	94841959	ENSG00000140563	MCTP2	ENST00000456504	constitutive	94841919	94841957	BEUR	Chan et al., 2009	UPF3A and UPF3B
chr15	94882519	94882661	ENSG00000140563	MCTP2	ENST00000456504	constitutive	94882622	94882659	BEUR	Chan et al., 2009	UPF3A and UPF3B
chr15	94943150	94943224	ENSG00000140563	MCTP2	ENST00000456504	constitutive	94943174	94943218	BEUR	Chan et al., 2009	UPF3A and UPF3B
chr15	94899366	94899530	ENSG00000140563	MCTP2	ENST00000456504	constitutive	94899479	94899512	BR	Chan et al., 2009	UPF3A and UPF3B
chr15	94901711	94901841	ENSG00000140563	MCTP2	ENST00000456504	altAcceptor	94901801	94901833	BR	Chan et al., 2009	UPF3A and UPF3B
chr15	94901776	94901841	ENSG00000140563	MCTP2	ENST00000557742	altAcceptor	94901801	94901833	BR	Chan et al., 2009	UPF3A and UPF3B
chr15	94986148	94986189	ENSG00000140563	MCTP2	ENST00000456504	variant	94986150	94986182	BR	Chan et al., 2009	UPF3A and UPF3B
chr15	94945129	94945248	ENSG00000140563	MCTP2	ENST00000456504	constitutive	94945200	94945243	BU	Chan et al., 2009	UPF3A and UPF3B
chr15	94884042	94884153	ENSG00000140563	MCTP2	ENST00000456504	constitutive	94884092	94884154	BUR	Chan et al., 2009	UPF3A and UPF3B
chr15	94913316	94913409	ENSG00000140563	MCTP2	ENST00000456504	constitutive	94913362	94913408	BUR	Chan et al., 2009	UPF3A and UPF3B
chr15	94841430	94841959	ENSG00000140563	MCTP2	ENST00000456504	constitutive	94841799	94841874	EUR	Chan et al., 2009	UPF3A and UPF3B
chr3	131217023	131217121	ENSG00000114686	MRPL3	ENST00000264995	variant	131217033	131217067	BER	Wang et al., 2014	eIF4A3 and BTZ
chr3	131219274	131219365	ENSG00000114686	MRPL3	ENST00000264995	constitutive	131219284	131219306	BER	Wang et al., 2014	eIF4A3 and BTZ
chr3	131206524	131206584	ENSG00000114686	MRPL3	ENST00000264995	constitutive	131206541	131206575	BEUR	Wang et al., 2014	eIF4A3 and BTZ
chr2	183066415	183066516	ENSG00000115252	PDE1A	ENST00000435564	constitutive	183066421	183066465	BE	Chan et al., 2007	UPF3B
chr2	183129028	183129141	ENSG00000115252	PDE1A	ENST00000435564	constitutive	183129044	183129086	BR	Chan et al., 2007	UPF3B
chr2	183070667	183070792	ENSG00000115252	PDE1A	ENST00000435564	constitutive	183070669	183070714	BU	Chan et al., 2007	UPF3B
chr2	183088591	183088691	ENSG00000115252	PDE1A	ENST00000435564	constitutive	183088603	183088624	BU	Chan et al., 2007	UPF3B
chr2	183094733	183094873	ENSG00000115252	PDE1A	ENST00000435564	constitutive	183094748	183094769	BU	Chan et al., 2007	UPF3B
chr4	57876527	57876670	ENSG00000047315	POLR2B	ENST00000381227	constitutive	57876632	57876664	BEUR	Chan et al., 2007	UPF3B
chr4	57883209	57883407	ENSG00000047315	POLR2B	ENST00000381227	constitutive	57883362	57883407	BEUR	Chan et al., 2007	UPF3B
chr4	57861417	57861575	ENSG00000047315	POLR2B	ENST00000381227	constitutive	57861533	57861573	BR	Chan et al., 2007	UPF3B
chr4	57883726	57883894	ENSG00000047315	POLR2B	ENST00000381227	constitutive	57883851	57883892	BU	Chan et al., 2007	UPF3B
chr4	57888318	57888412	ENSG00000047315	POLR2B	ENST00000381227	constitutive	57888373	57888409	BU	Chan et al., 2007	UPF3B
chr4	57891052	57891165	ENSG00000047315	POLR2B	ENST00000381227	constitutive	57891124	57891165	BU	Chan et al., 2007	UPF3B
chr4	57891543	57891703	ENSG00000047315	POLR2B	ENST00000381227	constitutive	57891652	57891703	BU	Chan et al., 2007	UPF3B
chr4	57896370	57896565	ENSG00000047315	POLR2B	ENST00000381227	constitutive	57896510	57896565	BU	Chan et al., 2007	UPF3B
chr4	57872982	57873168	ENSG00000047315	POLR2B	ENST00000381227	constitutive	57873118	57873191	BUR	Chan et al., 2007	UPF3B
chr1	186645030	186645845	ENSG00000073756	PTGS2	ENST00000490885	altDonor	186645602	186645655	BUR	Gehring et al., 2005	RNPS1
chr1	186645599	186645845	ENSG00000073756	PTGS2	ENST00000367468	altDonor	186645602	186645655	BUR	Gehring et al., 2005	RNPS1
chr5	98109657	98109910	ENSG00000174136	RGMB	ENST00000308234	altAcceptor	98109773	98109830	BR	Chan et al., 2007	UPF3B
chr5	98109660	98109910	ENSG00000174136	RGMB	ENST00000504776	altAcceptor	98109773	98109830	BR	Chan et al., 2007	UPF3B
chr5	98109660	98109910	ENSG00000174136	RGMB	ENST00000504776	altAcceptor	98109861	98109903	BR	Chan et al., 2007	UPF3B
chr17	38787844	38788619	ENSG00000073584	SMARCE1	ENST00000469334	altDonor	38788456	38788495	BER	Chan et al., 2007	UPF3B
chr17	38792647	38792778	ENSG00000073584	SMARCE1	ENST00000348513	constitutive	38792646	38792685	BEUR	Chan et al., 2007	UPF3B
chr17	38798707	38798811	ENSG00000073584	SMARCE1	ENST00000348513	variant	38798742	38798774	BR	Chan et al., 2007	UPF3B
chr17	38793744	38793824	ENSG00000073584	SMARCE1	ENST00000348513	constitutive	38793791	38793819	BUR	Chan et al., 2007	UPF3B
chr1	70701184	70701233	ENSG00000116754	SRSF11	ENST00000370950	constitutive	70701191	70701222	BE	Chan et al., 2007	UPF3B
chr1	70710367	70710498	ENSG00000116754	SRSF11	ENST00000370950	constitutive	70710377	70710382	ER	Chan et al., 2007	UPF3B
chr17	74732236	74732546	ENSG00000161547	SRSF2	ENST00000359995	altBoth	74732532	74732556	BER	Gehring et al., 2005	BTZ
chr17	74731854	74731957	ENSG00000161547	SRSF2	ENST00000582449	variant	74731855	74731899	BEU	Gehring et al., 2005	BTZ

Table S6: Candidate exons. Related to Figure 4.

chromosome	peak_start	peak_end	peak_constitution	peak_strand	motif	RNASeq_max	validated
chr1	31408390	31408421	BU	-	AGGTAAG	1.92454392	yes
chr1	70759193	70759249	BUR	-	AGGTAAG	1.567413308	yes
chr1	109489973	109490002	BU	-	AGGTGAG	1.738607591	yes
chr1	117533144	117533161	BU	+	AGGTGAG	0.017108138	yes
chr1	220943849	220943889	BUR	+	AGGTAAG	0.627329422	yes
chr1	232709928	232709963	BR	-	AGGTAAG	0.167478378	yes
chr2	28633019	28633134	BUR	+	AGGTGAG	2.437001638	yes
chr2	101924302	101924333	BU	-	AGGTGAG	1.644242086	yes
chr3	127295012	127295084	BEUR	-	AGGTGAG	2.673589249	yes
chr5	60820926	60820966	BU	+	AGGTAAG	0.740998643	yes
chr6	2773253	2773295	BU	+	AGGTGAG	0.748281238	yes
chr6	32084101	32084254	BEUR	-	AGGTGAG	0.76875387	yes
chr6	170876536	170876595	BEUR	+	AGGTAAG	1.038479446	yes
chr8	144885747	144885755	BU	-	AGGTGAG	0.37534245	no
chr9	100780727	100780771	BR	+	AGGTGAG	0.139927314	yes
chr9	139294585	139294641	BUR	-	AGGTGAG	0.077491378	yes
chr10	126650932	126650974	BU	+	AGGTAAG	0.98799819	yes
chr11	123351535	123351602	BUR	+	AGGTAAG	0.731953671	yes
chr12	133220306	133220421	BR	-	AGGTGAG	2.340115173	no
chr16	590272	590410	BR	+	AGGTAAG	0.300513055	yes
chr16	56939303	56939353	BE	+	AGGTAAG	1.420646329	yes
chr17	12032843	12032886	BU	+	AGGTGAG	0.600925841	yes
chr19	3784892	3784921	BR	-	AGGTGAG	1.11493953	no
chr19	10505588	10505696	BR	-	AGGTAAG	9.804446049	no
chr19	19229840	19229883	BER	-	AGGTGAG	0.165178692	yes
chr20	62514181	62514239	BR	+	AGGTGAG	4.811648049	yes
chr21	46627254	46627299	BUR	+	AGGTGAG	1.263387152	yes
chr22	44727378	44727444	BR	-	AGGTGAG	0.009703228	yes
chr22	50875345	50875414	BEUR	+	AGGTGAG	1.33428187	yes
chr22	50879503	50879632	BEUR	+	AGGTGAG	0.501333486	yes
chrX	18910367	18910405	BR	+	AGGTGAG	2.105646862	yes

Table S7: siRNAs, primers and antibodies used in this study. Related to Figure 1.

	siRNA sequence (5' to 3')	ID
**eIF4A3**	GGAAGTATTTAGATCCAGA	SI05098044
	CCCATAAACTCTATACTTCTA	SI00107842
**BTZ**	CTGCTGAGGGCCAATACCCTA	SI05014849
	CTCCCTATCCATGGCACTAAA	SI05014856
**RNPS1**	GCCGTTCATGGTGGTCTTTCA	SI05027743
	AAACCCAAGATCGGAATGCAA	SI05027750
**UPF3B**	AAGTGAGGACTTCATCACATA	SI05096406
	CGGCAAGGACATGCATTGCAA	SI05096413
**UPF3A**	TTGGGTTTCATTCGTTTGTAA	SI03246411

	Primer sequence (5´ to 3´)	used in final iCLIP experiment replicate (rep) number
**L3 linker**	/5rApp/AGATCGGAAGAGCGGTTCAG/3ddC	
**RT primer 1**	/5Phos/NNAACCNNNAGATCGGAAGAGCGTCGTGGATCCTGAACCGC	UPF3B rep 2, RNPS1 rep 1
**RT primer 2**	/5Phos/NNACAANNNAGATCGGAAGAGCGTCGTGGATCCTGAACCGC	not used
**RT primer 3**	/5Phos/NNATTGNNNAGATCGGAAGAGCGTCGTGGATCCTGAACCGC	BTZ rep 1, GFP rep 1, eIF4A3 rep 2, UPF3B rep 2, PTB rep 2
**RT primer 4**	/5Phos/NNAGGTNNNAGATCGGAAGAGCGTCGTGGATCCTGAACCGC	not used
**RT primer 5**	/5Phos/NNCGCCNNNAGATCGGAAGAGCGTCGTGGATCCTGAACCGC	PTB rep 1, GFP rep 2
**RT primer 6**	/5Phos/NNCCGGNNNAGATCGGAAGAGCGTCGTGGATCCTGAACCGC	not used
**RT primer 7**	/5Phos/NNCTAANNNAGATCGGAAGAGCGTCGTGGATCCTGAACCGC	not used
**RT primer 8**	/5Phos/NNCATTNNNAGATCGGAAGAGCGTCGTGGATCCTGAACCGC	not used
**RT primer 9**	/5Phos/NNGCCANNNAGATCGGAAGAGCGTCGTGGATCCTGAACCGC	not used
**RT primer 10**	/5Phos/NNGACCNNNAGATCGGAAGAGCGTCGTGGATCCTGAACCGC	eIF4A3 rep 2, PTB rep 2, GFP rep 3
**RT primer 11**	/5Phos/NNGGTTNNNAGATCGGAAGAGCGTCGTGGATCCTGAACCGC	not used
**RT primer 12**	/5Phos/NNGTGGNNNAGATCGGAAGAGCGTCGTGGATCCTGAACCGC	not used
**RT primer 13**	/5Phos/NNTCCGNNNAGATCGGAAGAGCGTCGTGGATCCTGAACCGC	not used
**RT primer 14**	/5Phos/NNTGCCNNNAGATCGGAAGAGCGTCGTGGATCCTGAACCGC	not used
**RT primer 15**	/5Phos/NNTATTNNNAGATCGGAAGAGCGTCGTGGATCCTGAACCGC	eIF4A3 rep 1, RNPS1 rep 1, BTZ rep 2
**RT primer 16**	/5Phos/NNTTAANNNAGATCGGAAGAGCGTCGTGGATCCTGAACCGC	UPF3B rep 1, RNPS1 rep 2, BTZ rep 3
**Cut Oligo**	GTTCAGGATCCACGACGCTCTTCAAAA	
**Solexa 5´**	AATGATACGGCGACCACCGAGATCTACACTCTTTCCCTACACGACGCTCTTCCGATCT	
**Solexa 3´**	CAAGCAGAAGACGGCATACGAGATCGGTCTCGGCATTCCTGCTGAACCGCTCTTCCGATCT	

	Forward primer (5' to 3')	Location	Reverse primer (5' to 3')	Location
**eIF4A3 endo**	GATGCCGATGAACGTTGCTG	Exon 11–12	TTCAAAGGAAGGGAGACGGC	3´UTR
**eIF4A3**	TCATCGCACAGTCTCAGTCC	Exon 2–3	TTCCTGATGTCCTCGCCAAC	Exon 5
**BTZ endo**	AGGAGTCGGAGTGTGAGAGT	Exon 1–2	AGTGCTTTTGGTGTCAGGCT	Exon 4
**BTZ**	AGCACATGGATGGAGGACAA	Exon 7–8	CAGGCACACAGCATCCAAAC	3´UTR
**RNPS1 endo**	CGCTGAAGCACATGGATGGA	Exon 7	GGAGCGGGACCTTCTCCT	Exon 8–9
**RNPS1**	ACCAAAGAGACCTGAAGATGAGA	Exon 9–10	ACAGTCAGGACACCTAAGGC	3´UTR
**UPF3B endo**	CAATAAAGGTCAGGAATATCCCGC	Exon 3–4	AGGAAGCTCAAAAGTGGGGT	Exon 6
**UPF3B**	GCGACTGGCAAACAAGGACC	Exon 9–10	CTCCTTCCCAGTCACACTCG	3´UTR
**UPF3A endo**	GCCCCAGGAGACGTCACACA	Exon 7–8	CACTCCTTCTGGAAAGCCGT	Exon 8
**SC35C**	GGCGTGTATTGGAGCAGATGTA	3´UTR	CTGCTACACAACTGCGCCTTTT	3´UTR
**SC35D**	CGGTGTCCTCTTAAGAAAATGATGTA	3´UTR	CTGCTACACAACTGCGCCTTTT	3´UTR
**SC35WT**	CGTGCCTGAAACTGAAACCA	3´UTR	TTGCCAACTGAGGCAAAGC	3´UTR
**TGM2**	GGGGTGAGAGAGGAAAGACC	Exon 13	AGTGCTTTTGGTGTCAGGCT	Exon 13
**IDUA**	GGCCATGGTGGTGAAGGTCAT	Exon 7–8	GAAGGCATTGTCGTTGCTCAG	Exon 8
**ATHL1**	TGGGACCAGGACCTCTGGATG	Exon 4–5	AGTCTGCACTCTCCCAGGCAA	Exon 6
**ZNF217**	ACTGTTACCGCAGGACTGTGT	Exon 3	TTTCCTTCTAACGTCGAGCTG	Exon 3–4
**ZCCHC2**	TTCTACTGAAGCTTCCAAAGG	Exon 4–5	GGAGTCTGATGATATGGACTG	Exon 6
**GAPDH**	TGAGCTTGACAAAGTGGTCG	Exon 8	GGCTCTCCAGAACATCATCC	Exon 8
**ZXDB**	TGTGTGACCCTGAGCAAGTC	3´UTR	GCCACATCACTGAAAAACGTCC	3´UTR
**HIST1H4B**	CTAGGCGTGGTGGGGTTAAG	Exon 1	CGTACAGAGTGCGTCCTTGA	Exon 1

	Concentration	Secondary antibody	Company	ID
**α-eIF4A3**	1:1,000	Rabbit	Genscript	custom-made*
**α-BTZ**	1:1,000	Rabbit	Abcam	ab90651
**α-RNPS1**	1:1,000	Rabbit	Gift from Dr. Akila Mayeda	
**α-UPF3B**	1:1,500	Rabbit	Sigma-Aldrich	HPA001800
**α-Y14**	1:1,000	Mouse	ImmuQuest	IQ220
**α-hnRNPA 1**	1:1,000	Mouse	Santa Cruz	sc-32301
**α-Actin**	1:1,000	Mouse	Sigma-Aldrich	A1978
**α-Tubulin**	1:1,000	Mouse	Life Technologies	480011
**α-GAPDH**	1:1,000	Goat	GeneTex	GTX89409
**α-GFP**	1:1,000	Rabbit	Abcam	ab290
**α-Mouse**	1:10,000	–	Sigma-Aldrich	A9044
**α-Rabbit**	1:10,000	–	Sigma-Aldrich	A0545
**α-Goat**	1:10,000	–	Sigma-Aldrich	A9452

*DDX48 custom made against ATSGSARKRLLKEEDC peptide

Supplemental Experimental Procedures

Stable HeLa cell lines

A HeLa host cell line containing a Flp recombination target (FRT) site combined with an antibiotic (Zeocin™) resistance gene and stably expressing a tetracycline repressor was used to generate cell lines expressing the fusion proteins. The gene of interest was cloned into a pcDNA5 vector containing a FRT and a tetracycline-on (TO) system, pcDNA5/FRT/TO, and integrated into the host cell line by the pOG44 Flp recombinase expressing plasmid. The FRT site located upstream of another antibiotic (hygromycin) resistance gene in the pcDNA5/FRT/TO vector was used for the integration of the fusion protein into the HeLa host cell line. In addition, the hygromycin resistance gene enabled the specific selection of colonies containing the fusion gene. The human *cytomegalovirus* (CMV) promoter together with the TO system allowed the expression of fusion proteins close to the level of the endogenous counterpart.

For each stable cell line, 1,000,000 cells were seeded in a 10 cm dish. On day 2, the medium was renewed 2 h before the transfection of the plasmids using the calcium phosphate method. For the transfection, pOG44 Flp recombinase plasmid was mixed with the pcDNA5/FRT/TO/TEV/Protein-GFP plasmids in a ratio of 1:9 and H2O was added to a final volume of 450 µl. The plasmids were then mixed with 50 µl of 2.5 M $CaCl_2$ and 500 µl of 2 x borate buffered saline (BBS). After incubation for 20 min at room temperature, the mixture was added to the cells in 10 ml Dulbecco's Modified Eagle Medium (DMEM) and incubated for 20 h at 37 °C and 5% CO2. On day 3, the cells were split into three 10 cm dishes using different ratios of cells (5:10, 3:10 and 2:10). On day 4, DMEM was exchanged by fresh DMEM containing the selection antibiotics blasticidin (final concentration 5 µg/ml) and hygromycin (final concentration 200 µg/ml). The cells were monitored for approximately two weeks. During this time, the medium was exchanged when appropriate. Single colonies were picked with 10 µl trypsin and transferred to a 12-well plate containing 1 ml DMEM with selection antibiotics (blasticidin and hygromycin) per well. On week 4, colonies were detached with 70 µl trypsin and transferred into two wells of a 6-well plate, each containing 2 ml DMEM with selection antibiotics. The expression of a GFP-fusion construct was controlled by fluorescence microscopy in one well by the addition of 1 µg/ml doxycycline, which belongs to the tetracycline antibiotics class and thus can be used in Tet-on systems to replace tetracycline. In case the fusion protein was expressed, the non-induced well was passaged to a T175 flask and, after the cells reached 80% confluence, frozen in two aliquots.

The stable HeLa cells were frozen in liquid nitrogen in batches to ensure that the experiments will be performed on freshly thawed cells from the same passage. The first experiments were conducted from passage 2 onwards and the cells were discarded at passage 10.

siRNA knockdown and complementation assay

For the siRNA rescue experiments, 100,000 cells in 2 ml DMEM GlutaMax-I medium (supplemented with 10% (v/v) FCS and 1% (v/v) penicillin/streptomycin) were seeded per well of a 6-well plate. The cells were transfected with siRNAs 6–8 h after seeding. For each sample, 1 µl siRNA (siRNA stocks 10 pmol/µl) was diluted in 100 µl pre-warmed reduced serum medium (Opti-MEM®) without antibiotics and 12 µl transfection reagent (HiPerfect) were added. The mixture was vortexed and incubated for 10 min at room temperature. In the meantime, the cells were washed twice with Opti-MEM®. The siRNA mixture was added drop-wise to 887 µl Opti-MEM per 6-well. Thus, the cells were incubated overnight with a siRNA concentration of 10 nM. On the following day, Opti-MEM® was replaced by 2 ml DMEM containing appropriate doxycycline concentration for the induction of the fusion proteins (for RNPS1 0.1 ng/ml and for the other EJC proteins and controls 1 ng/ml doxycycline). The cell samples were split for RNA and protein analysis. The RNA was isolated by 750 µl of TRIzol® and the cDNA synthesis was performed with oligo-(dT) primer. The protein sample was centrifuged at 1100 g for 10 min at 4 °C. After removal of the supernatant, the cells were lysed in 50 µl RIPA buffer (150 mM NaCl, 0.5% (w/v) sodium deoxycholate, 50 mM Tris-Base, 1% (w/v) nonyl phenoxypolyethoxylethanol (NP-40), 0.1% (w/v) SDS and 1 x protease inhibitor) and incubated for 10 min on ice. The protein samples were stored at −80 °C prior to immunoblot analysis. The siRNA and primer sequences are listed in Table S7.

Immunoblots

For immunoblots, proteins were separated using SDS-PAGE. The protein samples were supplemented with 6 x loading buffer and heated for 5 min at 95 °C. Protein size was determined using 10 µl of the protein ladder (PageRuler™ Plus). After electrophoresis at 20–60 mA, protein transfer was carried out at 1mA/cm2 of the membrane (Westran-S) for 1 h. To slow down this protein transfer for small proteins, like Y14 (20 kDa), the anode and cathode buffers were supplemented with 20% methanol. The membrane was incubated for 1 h in 5% skim milk in Tris-buffered saline plus Tween (TBS-T) to block unspecific binding sites prior to the antibody labeling. The primary antibody was incubated under gentle shaking overnight at 4 °C (see Table S7 for antibodies). Unbound antibody was removed by three washing steps for 10 min with TBS-T at room temperature. The secondary antibody was incubated for 1 h at room temperature. Subsequently, three washing

steps, each of 10 min at room temperature with TBS-T, were carried out to remove unbound secondary antibody prior to the protein detection. The proteins were labeled with a chemiluminescence kit and detected with a multi-imaging system (Fusion Fx).

iCLIP

Day 1 and 2 – Preparation of stable cells

For each iCLIP replicate, 3,000,000 stable HeLa cells were seeded in two 15 cm dishes, each containing 30 ml DMEM supplemented with 10% (v/v) FCS and 1% (v/v) penicillin/streptomycin. The cells were incubated at 37 °C and 5% CO2 for two days. The GFP-fusion constructs were induced directly after seeding with a final doxycycline concentration of 1 ng/ml (0.1 ng/ml for RNPS1-GFP) to adjust the expression level of the recombinant protein close to that of the endogenous counterpart.

Day 3 – UV cross-linking

To stop protein translation, the cells were treated with a final concentration of 100 µg/ml cycloheximide. A cross-link between the RBP and the bound RNA was mediated by an irradiation with 150 mJ/cm^2 and a wavelength of 254 nm.

Day 4 – Immunoprecipitation

After harvesting the cells, the cells were lysed (all buffers as in (Konig et al., 2011)) and protein concentration was adjusted to approximately 2–3 mg/ml and a final volume of 1400 µl. To increase the salt concentration, 350 µl of 5 x high salt buffer were added to the sample. Meanwhile, 30 µl of agarose beads coupled to a GFP antibody (GFP-Trap®_A) were washed three times with 1 ml lysis buffer and spinning at 1000 g at 4 °C for 1 min. The beads were resuspended in 60 µl lysis buffer and transferred to the sample. To resuspend the beads thoroughly, the pipette tip had to be cut using a sharp blade. The sample was incubated at 4 °C and 10 rpm in a rotator mixer (RM Multi-1) for 2 h.
The proteins bound to antibody-coupled beads were pelleted at 1000 g at 4 °C for 30 s and the supernatant was stored as flow-through. All subsequent washing steps were performed with 1 ml washing buffer and a 2 min incubation at 10 rpm in a rotator mixer followed by centrifugation at 1000 g for 1 min. To remove unbound proteins, the beads were washed twice with high salt buffer at room temperature to avoid precipitation of SDS and protease inhibitors. The samples were further purified by two washing steps with medium salt buffer and one washing step with low salt buffer containing only 0.5 x of RNase inhibitors (RNasin). For the partial RNA digestion, the sample was resuspended in 1 ml low salt buffer without RNasin and 10 µl of 1:50 dilution of RNase I (final concentration 1:5000, 0.02 U/µl) and 2 µl of Turbo DNase. The RNA was partially digested by incubation for exactly 3 min at 37 °C at 1100 rpm in a heat block (Thermomixer compact). Subsequently, the beads were washed with 1 ml of high salt buffer containing protease and RNase inhibitors at room temperature, followed by two washing steps with low salt buffer.
To dephosphorylate the 3´end of the RNA fragments, the beads were resuspended in 20 µl PNK mix (15 µl H2O, 4 µl 5 x PNK buffer pH6.5, 0.5 µl PNK and 0.5 µl RNasin) and incubated at 37 °C for 20 min at 1100 rpm in a heat block. The enzyme was inactivated by two high salt and two low salt buffer washing steps. For ligation of a L3 linker to the RNA fragments – being essential for the reverse transcription – the supernatant was removed and the sample incubated in 20 µl ligation mix (9 µl H2O, 4 µl 4 x ligation buffer, 1 µl RNA ligase, 0.5 µl RNasin, 1.5 µl 20 µM pre-adenylated linker L3 and 4 µl polyethylene glycol (PEG) 400) at 16 °C using 1100 rpm overnight in a heat block.

Day 5 – RNA isolation

Unligated L3 linker was removed by three washing steps with high salt buffer (containing protease and RNase inhibitors). After the second washing step, the samples were transferred into new low DNA binding tubes to remove potentially non-ligated L3 linker bound to the plastic surface. Subsequently, the samples were washed twice with low salt buffer containing RNase inhibitors (but no protease inhibitors) to prepare the samples for protein digestion. In order to digest any RNases in the proteinase K (PK) buffer, the buffer was treated with proteinase K (10 µl) at 37 °C for 20 min. Afterwards, 200 µl of this mixture were transferred to each sample and the tubes were incubated at 37 °C. After 20 min, 200 µl PK-Urea buffer were added to each sample followed by an incubation for another 20 min at 37 °C to denature the proteins.
The RNA was isolated using phase lock gel (PLG™) heavy tubes. To facilitate the RNA precipitation, the samples were mixed with 0.5 µl glycogen (GlycoBlue™) and 40 µl 3M sodium acetate pH 5.2. The precipitation was performed with 1 ml 100% ethanol at −20 °C overnight.

Day 6 – cDNA synthesis

The precipitated RNA was pelleted at 16000 g at 4 °C for 20 min. After removal of the supernatant, the pellet was washed with 80% (v/v) ethanol and centrifuged for another 2 min. Ethanol was completely removed and the pellets were air-dried for 2 min at room temperature. A volume of 6.25 µl was further transferred to a new 200 µl tube to continue the iCLIP experiment. The remaining 1.75 µl were used to measure the size and concentration of the isolated RNA with a Bioanalyzer2100 instrument and RNA Pico chips. The size distribution of the RNA should have a peak within 25–300 nts.
For iCLIP, 0.5 µl RT primer (0.5 pmol/µl) and 0.5 µl dNTP (10 mM each) were mixed with the sample and incubated for 5 min at 70 °C in a PCR machine. For each replicate and experiment performed in one batch, a different RT primer was used. Table S7 lists all possible RT primer sequences including the barcodes. The RT primers contained a sample and a random barcode. The sample barcode allows multiplexing of samples and the random barcode enables the detection and elimination of PCR duplicates. The RT primers were checked for their efficiency in reverse transcription reactions by subcloning, using TA cloning and sequencing before final use in iCLIP experiments. In brief, for each iCLIP library with different RT primers at least 5 colonies were picked and the plasmids isolated. The plasmid sequences were evaluated regarding meaningful inserts, i.e. fragments upstream of exon-exon junctions for the EJC iCLIP experiments and, even more importantly, if the introduced sample barcode was present. Only RT primers that fulfilled these criteria were used for the subsequent iCLIP experiments. More specifically, the barcodes used were swapped to other RBPs and to the GFP control in further biological replicates in order to rule out any bias that was introduced by a single RT primer.
For iCLIP experiments, the RT primers were annealed to the L3 linker by slowly cooling down (−0.1 °C/s) the sample from 70 °C to 25 °C. For the reverse transcription, 2.75 µl reverse transcriptase mix (2 µl 5 x reverse transcriptase buffer, 0.5 µl 0.1M DTT and 0.25 µl Superscript III) were added. The second cDNA synthesis step was performed at 50 °C instead of the initial 42 °C in order to improve the denaturing of RNA secondary structures. The mixture was diluted with 90 µl Tris-EDTA (TE) buffer and transferred to a new low DNA binding tube containing 1 µl glycogen and 10 µl 3M sodium acetate pH 5.2. After vortexing, the cDNA was precipitated with 250 µl 100% ethanol at −20 °C overnight.

Day 7 – cDNA size selection

The precipitated cDNA was pelleted, washed as described at day 6 and resuspended in 6 µl H2O. For the gel purification of the cDNA, the same volume of 2 x Tris-borate EDTA urea (TBE-urea) loading buffer was added. For one gel, 3 µl of low molecular weight DNA ladder were mixed with the same volume of 2 x TBE-urea loading buffer. The samples and the marker were heated for 3 min at 80 °C. The samples plus the marker were loaded on the gel and exposed to a voltage of 180V for approximately 40 min. Afterwards, slices of low, medium and high molecular weight cDNAs were cut from the gel according to a size range of 70–85, 85–120 and 120–200 nts. Each gel slice was transferred to a 1.5 ml low DNA binding tube and 400 µl TE buffer were added. The gel slice was crushed into small pieces using a pestle and incubated for 2 h at 37 °C under agitation at 1100 rpm. Meanwhile, two glass microfiber filters were placed into a centrifuge tube filter column using clean tweezers. Each sample was transferred to one of these columns and centrifuged for 1 min at 16,000 g at room temperature. The flow-through was transferred to a low DNA binding tube containing 0.5 µl glycogen and 40 µl 3M sodium acetate pH 5.2. The cDNA was precipitated with 1 ml 100% ethanol at −20 °C overnight.

Day 8 – cDNA circularization

The size-selected and precipitated cDNA was pelleted, washed as described at day 6, resuspended in 8 µl ligation mix (6.5 µl H2O, 0.8 µl 10 x CircLigase buffer II, 0.4 µl 50 mM MnCl2 and 0.3 µl CircLigase II) and transferred to a PCR tube. For circularization of the cDNA, the samples were incubated for 1 h at 60 °C in a pre-heated PCR machine to avoid evaporation of the sample into the tube lid. Afterwards, 30 µl of oligonucleotide annealing mix (26 µl H2O, 3 µl SmartCut buffer and 1 µl 10 µM Cut Oligo, see Table S7) were added. The sample was incubated at 95 °C for 1 min and then the temperature was decreased by 1 °C/10 s to a final temperature of 25 °C. The annealed oligonucleotide was cut by 2 µl of the restriction enzyme BamHI-HF for 30 min at 37 °C. The sample was mixed with 50 µl TE buffer and transferred to a low DNA binding tube containing 1 µl glycogen and 10 µl 3M sodium acetate pH 5.2. The cDNA was precipitated with 250 µl 100% ethanol at −20 °C overnight.

Day 9 – cDNA library preparation

The precipitated cDNA was pelleted, washed as described at day 6, resuspended in 19 µl H2O and transferred to a PCR tube. For the PCR amplification (25 cycles), 1 µl of 10 µM 5´ and 3´Solexa primer and 20 µl DNA polymerase (Accuprime Supermix 1) were added to the cDNA.
Afterwards, primer dimers and fragments smaller than 130 nts were removed from the cDNA library by magnetic beads (AMPure® XP) coated on their surface with carboxyl molecules, which can bind DNA molecules. The beads were vortexed and equilibrated for 30 min at room temperature. To each library, beads in a

volume corresponding to the 1.8 volume of the cDNA sample were added. In practice, 35 µl of cDNA library were diluted to a total volume of 50 µl with H2O and 90 µl of beads were added. After vortexing, the sample was incubated for 15 min at room temperature. Then, the tube was put to a magnetic particle concentrator (DynaMagTM-2) for 5 min to capture the cDNAs that were longer than 130 nts. The tube was maintained at the magnet until the elution step. The supernatant was discarded and 200 µl of freshly prepared 80% (v/v) ethanol was added for 30 s to wash the beads without disturbing the bead pellet. This step was repeated and then the ethanol was completely removed by incubating the tubes with open lid for 5 min at room temperature. To elute the captured cDNAs, the pellet was resuspended in 15 µl H2O and incubated for 2 min at room temperature. The tube was placed back to the magnetic stand for 5 min to remove the beads. The supernatant, containing the purified cDNA iCLIP library, was transferred to a low DNA binding tube and the purity was analyzed with a high sensitivity DNA chip. Therefore, 1 µl of the samples was diluted 1:10 with H2O and the analysis was performed according to the user manual [109]. The iCLIP library should only contain one peak corresponding to the selected cDNA size plus the added primer length. In general, the iCLIP library should be free from any primer dimers around 120 nts.
For the final sequencing, only the medium and high molecular weight cDNA libraries were submitted, since the low molecular weight cDNA libraries showed low complexity for the iCLIP reads in the test runs. Prior to multiplexing, the cDNA-concentrations of the libraries were measured with a Qubit™ dsDNA high sensitivity assay. Purified libraries with a fragment size in a range of 150–300 nts were pooled in equimolar ratios to a final cDNA concentration of 2 µg/µl and a volume of at least 30 µl. The cDNA libraries were sequenced with 50 bp single-end on an Illumina HiSeq2000.

RIP

For RNA extraction, the beads (GFP-Trap®_A) were washed four times with low salt buffer supplemented with 0.1% RNasin. To dissociate the complex from the bound RNA, 40 µl clear sample buffer (100 mM Tris-HCl pH 6.8, 4% SDS, 10 mM EDTA and 100 mM DTT) were added and the protein-bead samples were centrifuged for 5 min at 1000 g at room temperature. The input and the precipitated samples were diluted in 200 µl PBS and 750 µl TRIzol® to prepare them for the RNA isolation. The isolated RNA was reverse transcribed into cDNA and the samples were spiked with 2 µl TATAA universal RNA, which served as a quality control throughout the entire experimental workflow. For each RBP, three independent biological experiments were performed. To minimize the error in the q-RT-PCR every target was measured in triplicate and reported in C_t (threshold cycle) values for each independent experiment. The arithmetic mean of the technical replicates ($C_{t\ mean\text{-}triplicate}$) had to be adjusted by a dilution factor since the same RNA quantity for cDNA synthesis was used for each RBP. This dilution factor ($C_{t\ \mathrm{dilution\text{-}factor}}$) was calculated by the negative logarithm of the ratio between the RNA quantity that was used for the cDNA synthesis ($RNA_{cDNA\text{-}synthesis}$) and the RNA quantity retained after RIP ($RNA_{after\ RIP}$).

$$C_{t\ \mathrm{dilution-factor}} = -\log 2\ \mathrm{RNA}_{cDNA\text{-}synthesis} / \mathrm{RNA}_{afterRIP}$$

To calculate the relative RNA concentration of the target ($C_{t\ target\text{-}adjusted}$) this dilution factor has to be subtracted from the $C_{t\ mean\text{-}triplicate}$.

$$C_{t\ target-adjusted} = C_{t\ mean-triplicate} - C_{t\ dilution-factor}$$

This equation was applied to every RBP for both immunoprecipitation and input samples and the target enrichment ($C_{t\ enrichment}$) was calculated by subtracting the immunoprecipitation from the input values.

$$C_{t\ enrichment} = Input\ C_{t\ target-adjusted} - IP\ C_{t\ target-adjusted}$$

Afterwards, the target enrichment was adjusted (C*t enrichment-adjusted*) for the loss of RNA during the entire workflow by subtracting the spike-in Ct enrichment from the Ct enrichment target values.

$$C_{t\ enrichment-adjusted} = Target\ C_{t\ enrichment} - Spike{-}in\ C_{t\ enrichment}$$

Finally, for every target the percentage of the immunoprecipitated RNA compared to the input RNA (*Percentage of IP Input*) was calculated to determine which target RNA binds best to the RBPs after RIP.

$$Percentageof\ IP\ Input = 100 \times 2^{Ct\ enrichment-adjusted}$$

Peak analysis

Because the replicates had very similar distribution patterns across the different genomic regions, the peak analysis was repeated after merging the read-files of the three replicates. Consequently, the detection of peaks became more powerful with the increased number of reads. The detected peaks per RBP were filtered for high-confidence targets by applying a filter based on the GFP control. The number of peaks before and after the GFP filtering is listed in Table S3.

The number of false-positive hits was lowered by filtering for high GFP counts. The mean overamplification rate for the three biological replicates of GFP was divided by the mean overamplification rate of the other five proteins. This overamplification-factor was multiplied to the iCLIP read signal. We then applied a two step filtering approach. First, all peaks were removed that have a higher overamplification-factor adjusted CPM value in the GFP control than in the other libraries. Second, we scanned the individual replicates of an iCLIP experiment and kept only the peaks that have a higher summed CPM value compared to the GFP control. The overlap of the detected peaks was calculated with the software R (version 3.1.2) applying the Bioconductor package GenomicRanges (version 1.18.4) (Lawrence et al., 2013).

2 out of 4 approach

In order to analyze whether different proteins bind to the same genomic location, binding sites of different RBPs which overlapped with at least one nucleotide were merged into one contig. As a result, the number of detected binding sites was reduced from 521,171 to 346,396. The RNA binding to specific sites was controlled and confirmed by analysis of the PTB iCLIP dataset which possessed over 80% of unique binding sites that were not shared with any of the EJC components (see restricted peaks to one RBP in Table S3). By contrast, eIF4A3 and UPF3B shared 93% and 88% of the binding sites (showing only 7% and 12% unique binding sites), respectively. Similarly, BTZ and RNPS1 (with comparable or even larger libraries than PTB) accounted for less unique peaks (54% and 39%) compared to PTB. This indicated that all four EJC components share common sites where they bind directly to the RNA in close proximity and thus form EJCs with a homogeneous composition.

To further study the co-deposition of different EJC proteins as stringently as possible, all PTB binding sites were excluded from the analysis. This filtering resulted in approximately 200,000 sites at which at least one EJC protein, but not PTB, was deposited (Table S3).

This analysis was further specified to the EJC function by analyzing sites that were occupied by at least two out of 4 EJC proteins (Table S3 bottom part). These binding sites were termed high-confidence binding sites.

Supplemental References

Konig, J., Zarnack, K., Rot, G., Curk, T., Kayikci, M., Zupan, B., Turner, D.J., Luscombe, N.M., and Ule, J. (2011). iCLIP--transcriptome-wide mapping of protein-RNA interactions with individual nucleotide resolution. Journal of visualized experiments : JoVE.

Lawrence, M., Huber, W., Pages, H., Aboyoun, P., Carlson, M., Gentleman, R., Morgan, M.T., and Carey, V.J. (2013). Software for computing and annotating genomic ranges. PLoS computational biology *9*, e1003118.
